# Interfacial W–O–Zr ensembles in tungstated zirconia catalysts enable efficient hydrogen-free recycling of polypropylene waste

**DOI:** 10.1038/s41467-026-73420-6

**Published:** 2026-05-22

**Authors:** Sibei Zou, Yuzhen Ge, Patrik O. Willi, Javier Fernández-González, Robert N. Grass, Wendelin J. Stark, Gonzalo Guillén-Gosálbez, Antonio J. Martín, Javier Pérez-Ramírez

**Affiliations:** 1 Institute of Chemical and Bioengineering, Department of Chemistry and Applied Biosciences, ETH Zurich, Zurich, Switzerland; 2 NCCR Catalysis, Zurich, Switzerland

**Keywords:** Heterogeneous catalysis, Chemical engineering, Sustainability

## Abstract

Polyolefins are essential plastics to modern life yet create mounting sustainability challenges. Chemical recycling technologies are key but often require harsh conditions, costly hydrogen, noble metals, or complex catalysts with challenging design. We report hydrogen- and solvent-free depolymerization of polypropylene consumer goods at 240 °C, achieving >80% yield into gasoline hydrocarbons with stable performance. A library of tungstated zirconia catalysts synthesized by flame spray pyrolysis with controlled tungsten speciation enables correlating performance with the density of W–O–Zr ensembles prevalent in sub-nanoclusters, with W:Zr = 1:9 emerging as optimal. These acid sites formed during reaction mediate internal hydrogen transfer and selective backbone C–C scission, as deduced from *operando* spectroscopy. Life cycle and technoeconomic studies, benchmarked against hydrotreatment-based recycling in a harmonized framework, indicate competitive environmental and economic advantages. Together, this work establishes this route’s promise and underscores the value of precision catalyst synthesis in polyolefin valorization.

## Abstract (web summary)

Chemical recycling of plastics often requires harsh conditions, costly hydrogen, noble metals, or complex catalysts with challenging design. Here, the authors report hydrogen- and solvent-free depolymerization of polypropylene consumer goods at mild temperatures, achieving >80% yield into gasoline hydrocarbons with stable performance.

## Introduction

Polyolefins are plastics at the core of modern life with no foreseeable replacement in sight, making chemical recycling technologies play a central role in advancing a sustainable and circular society^1–5^. Alternative strategies to the largely studied and commercially available pyrolysis^6–8^, such as hydrotreatments have gained momentum in research laboratories worldwide due to their milder conditions and lighter downstream processing^9–12^. Their practical deployment, however, relies on enhancing catalytic performance and resolving uncertainty related to future green hydrogen supply^13–17^.

Hydrogen-free depolymerization at mild conditions has been recently demonstrated and so far focused on polyethylene^18^. Reports include alkane/polyolefin tandem cracking^19^, polyolefin dehydrocyclization and aromatization^20–22^, and internal hydride transfer through Lewis acid site, LAS-Brønsted acid site, BAS synergy^23,24^. For instance, aluminum chloride in ionic liquids with Lewis acidity enable tandem cleavage with isobutane or isopentane at temperatures as low as 100 °C, yielding C_4_–C_36_ liquid alkanes^19^. Pt/Al_2_O_3_ promotes dehydrocyclization and aromatization offering incipient but encouraging performance, with halogenated supports accelerating tandem reactions by increasing acidity^20–22^. More recently, WZr-KIT-6 (W- and Zr-incorporated KIT-6 silica materials) with HZSM-5 in physical mixtures were claimed to yielding C_4_–C_12_ hydrocarbons from polyethylene^23,24^. In these systems, the WZr-KIT-6 component is thought to pre-crack polymer chains on LAS and BAS, while C–C bond scission and product selective shaping occurs predominantly within the micropores of HZSM-5. This study suggests the challenge of integrating efficient acid functions within a single catalytic surface ^24^.

These systems exhibit a degree of structural complexity hindering unambiguous structure-performance correlations. As an example, acidity is frequently invoked as a key catalytic factor^21–24^; yet correlations between total acidity or simple Brønsted/Lewis ratios and performance have not been identified. Whether this reflects the complexity of the systems or a more fundamental aspect of the reaction chemistry remains unclear. The resulting uncertainty, compounded by a lack of *operando* studies, underscores the need for structurally well-defined catalysts that enable reliable structure-function assessment. A second critical knowledge gap lies in early feasibility evaluation, as few chemical recycling technologies for plastics have been benchmarked using harmonized environmental and economic metrics ^25–28^.

Here, we address these gaps by investigating the first efficient catalytic depolymerization of polypropylene (PP) in the absence of hydrogen or any other reductant or auxiliary means at mild temperatures^29–35^. We develop a library of materials showing that zirconia-supported sub-nano clusters of tungsten generated via flame spray pyrolysis achieve over 90% polypropylene conversion with ~90% selectivity to gasoline-range hydrocarbons and identify W–O–Zr as active sites through hydrogen transfer^36–38^ via *operando* characterization. Process simulations coupled to sustainability assessments including hydrotreatments show that hydrogen-free PP chemical recycling offers environmental and economic advantages. Taken together, this work suggests interfacial M–O–M’ (M = metal) linkages as a design principle for feasible pathways to polyolefin waste valorization.

## Results

### Catalyst synthesis strategy and polypropylene waste depolymerization

**Fig. 1:**
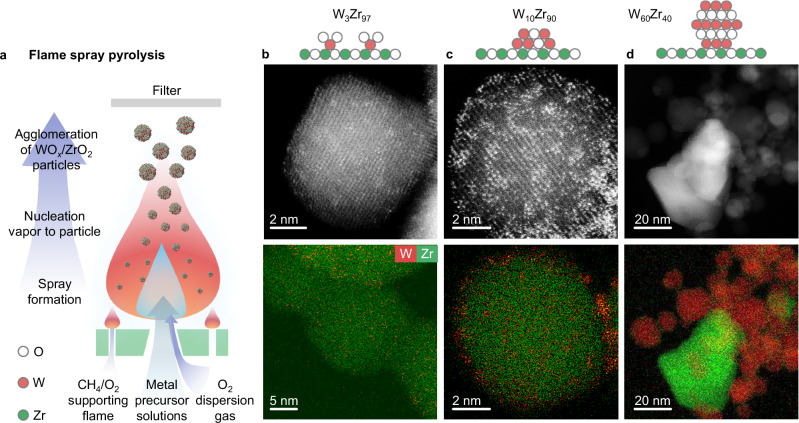
Synthesis route and nanostructure of W_a_Zr_b_ catalysts. **a** Schematic of the FSP synthesis process and representative STEM-HAADF images and elemental EDS mapping of as-prepared, **b** W_3_Zr_97_ showing isolated [WO_4_]^2−^ species, **c** W_10_Zr_90_ showing WO_*x*_ sub-nanoclusters, and **d** W_60_Zr_40_ showing WO_3_ nanoparticles on zirconia nanospheres.

Flame spray pyrolysis, FSP was selected to synthesize a series of tungstated zirconia catalysts with well-defined structural features (denoted W_a_Zr_b_, where a and b represent the mol% of tungsten and zirconium, respectively; Fig. 1a, Supplementary Table 1) in view of the versatility of this technique in producing materials with controlled architectures across wide compositional ranges^39–41^. STEM-HAADF imaging exploiting the high Z contrast between W and Zr revealed that all samples featured ca. 10 nm ZrO_2_ spheres (Supplementary Fig. 1), discarding support effects across the series, and decorated with distinct tungsten architectures. At low W contents (e.g., W_3_Zr_97_), uniformly dispersed isolated W atoms were prevalent with the presence of scattered sub-nanoclusters (Fig. 1b and Supplementary Figs. 2, 3). As the W content increased in W_10_Zr_90_, 0.3–0.8 nm WO_*x*_ sub-nanoclusters preferentially anchored at surface edge defects and grain boundaries of ZrO_2_ emerged as the predominant speciation compared to isolated W centers (Fig. 1c and Supplementary Fig. 2)^30,42^. At higher W contents, as in W_60_Zr_40_, irregular WO_3_ nanoparticles (5–40 nm) populated the ZrO_2_ surface accompanied by a minor fraction of sub-nanoclusters (Fig. 1d and Supplementary Fig. 3). X-ray diffraction, XRD analyses confirmed the gradual structural change characterizing the series (Supplementary Fig. 4).

**Fig. 2:**
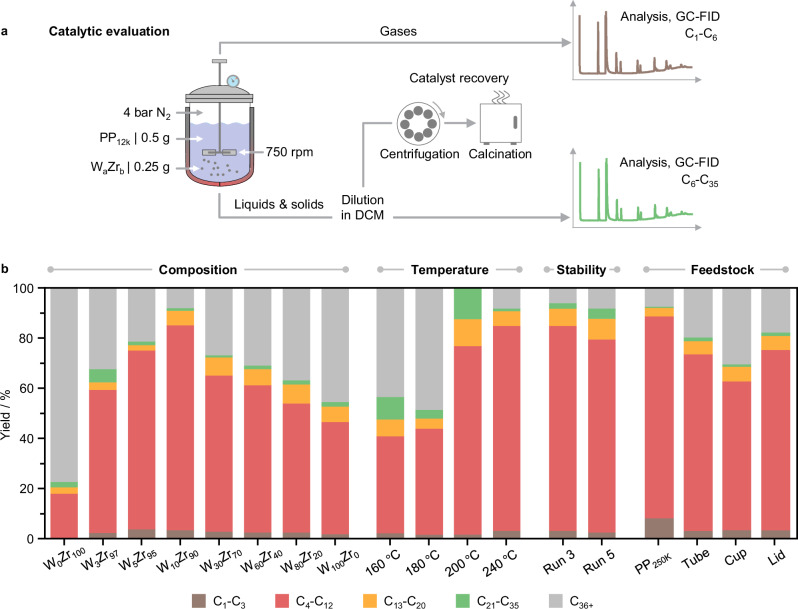
Experimental setup for polypropylene depolymerization and overview of catalytic performance over W_a_Zr_b_ catalysts. **a** Schematic of the catalyst evaluation process with indication of general conditions, product analysis, and catalyst recovery. **b** Performance for PP_12k_ depolymerization over catalysts with variable composition. W_10_Zr_90_ was selected to evaluate the influence of temperature (160–240 °C), stability (five consecutive runs), and PP-based feedstocks (PP_250k_, centrifuge tubes, yoghourt cups, and shampoo lids). Additional reaction conditions to those in (a): composition and stability: 240 °C, 4 h, temperature: 4 h, feedstock: 240 °C, 4–12 h. Detailed reaction conditions for each experiment are provided in Supplementary Tables 2,3,6,8. DCM stands for dichloromethane.

Virgin PP with a weight average molecular weight, *M*_w_ = 12 kDa, denoted as PP_12k_, was efficiently depolymerized over W_a_Zr_b_ catalysts under mild conditions similar to polyethylene reports (Fig. 2a)^20–24^. Product distribution analysis revealed a volcano-type dependence between performance and tungsten content, with W_10_Zr_90_ giving >90% conversion and ca. 90% selectivity to gasoline range (C_4_–C_12_) hydrocarbons (Fig. 2b, Supplementary Table 2, Supplementary Fig. 5)^23,24^. Notably, experiments at different reaction times over this catalyst disclosed its high activity. After 10 min, 75% conversion with 88% gasoline selectivity was achieved^43,44^, whereas prolonged durations of up to 24 h slowly shifted the product spectrum toward C_4_–C_5_ light gases (Supplementary Figs. 6, 7 and Supplementary Table 3). A more detailed analysis at short reaction times reveals a bimodal pattern centered at C_4_–C_10_ and C_25_–C_28_ (Supplementary Fig. 7). We propose two non-exclusive rationales requiring further investigations: (i) a carbenium-mediated *β*-scission pathway that favors near split-in-two cleavage of long chains^24,45^; and (ii) a site-spacing effect, whereby the separation between acid sites biases scission toward these characteristic segment lengths. Increasing N_2_ pressure resulted in a slight decrease of PP conversion with negligible changes in the selectivity pattern likely due to the hindered removal of volatile products from the melt (Supplementary Table 3, Supplementary Fig. 8).

Experiments at different reaction temperatures over W_10_Zr_90_ provided new mechanistic insights. At 160 °C, near the PP_12k_ melting point (157 °C), 60% conversion and 68% selectivity to gasoline-range hydrocarbons, accompanied by relatively high fractions of heavy hydrocarbons (C_13_–C_30_) characterized the product distribution (Fig. 2b, Supplementary Table 3). Increasing to 180 °C drove to mildly enhanced gasoline hydrocarbons yield, consistent with selective fragmentation of long chains. At 200 °C, full conversion was achieved with a still significant C_13_–C_30_ fraction, whereas 240 °C further enhanced gasoline selectivity to 89%, evidencing secondary cracking into shorter chains (Supplementary Fig.9). Increasing temperature thus progressively narrowed the product distribution into the C_4_–C_10_ range.

Proton nuclear magnetic resonance, ^1^H NMR analysis of the products obtained at 240 °C showed that the liquids consisted predominantly of alkanes with <3% aromatics (Supplementary Fig. 10, Supplementary Table 4). The quantification of gaseous products yielded ~30% olefins and ~60% iso-alkanes (Supplementary Fig. 10, Supplementary Table 5). The abundance of saturated hydrocarbons could be explained upon elemental analysis of the solid residue, displaying a gradual decrease of its H:C molar ratio from 2.1 for virgin PP_12k_ to 1.8 after 24 h (Supplementary Fig. 11), suggesting internal hydrogen transfer within partially unreacted fragments or a dehydrogenation mechanism more prevalent on long hydrocarbon fragments requiring further investigations, supported by the presence of small amounts of H_2_ in the products (0.2% yield, Supplementary Fig. 12). Evaluation at different catalyst-to-plastic ratios (1:2 and 2:1, w/w, Supplementary Fig. 13, Supplementary Table 3) confirmed C_4_–C_7_ as a lower scission limit that governs selective gasoline hydrocarbons formation. Mechanistically, this can be rationalized by the structure of PP under a carbenium ion-mediated scission, since tertiary carbenium ion centers formed at methine carbons are the most stable ones^22^. Since the smallest fragment capable of hosting a tertiary carbenium ion is iso-C_4_, secondary depolymerization may stall around C_4_–C_5_, with C_6_–C_7_ arising from neighboring scission events (and subsequent hydrogen transfer). This reasoning also supports the progressive increasing catalytic activity and narrowing of the product distribution around C_4_–C_6_ products as reaction temperature increases while keeping the same reaction time (Supplementary Table 3).

**Fig. 3:**
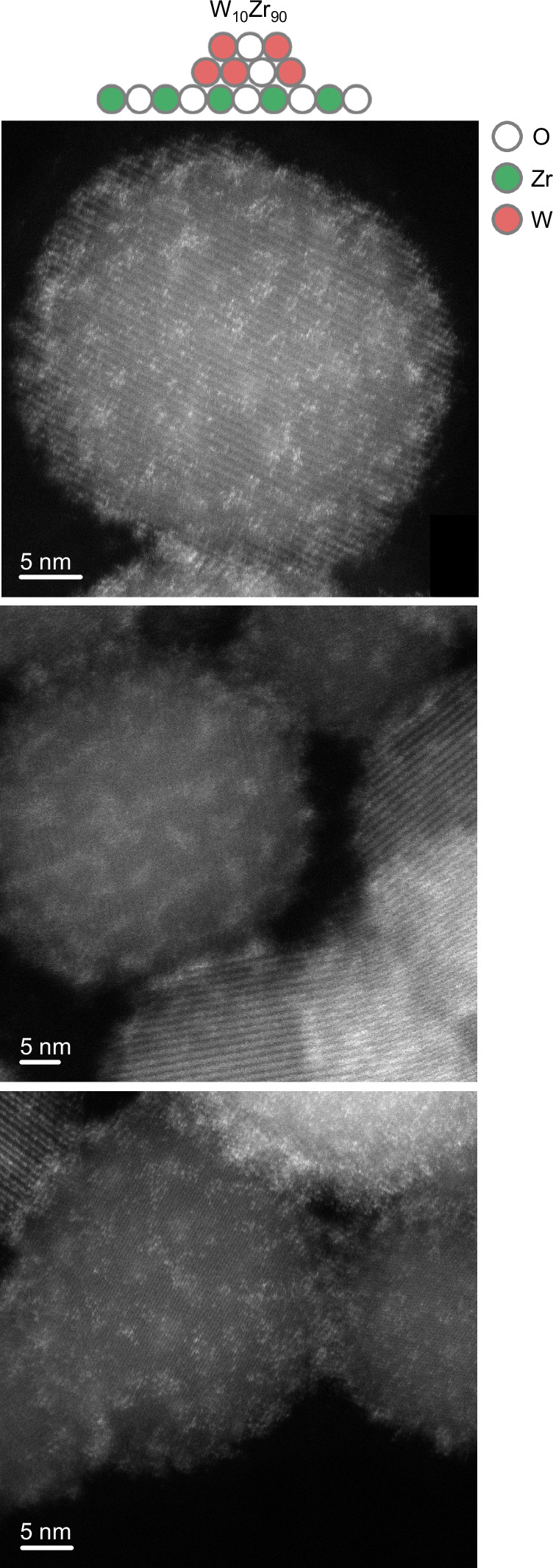
Structural stability of W_10_Zr_90_ after recyclability test. Representative STEM-HAADF images of W_10_Zr_90_ after five consecutive catalytic tests for the depolymerization of PP_12k_. Reaction conditions: 0.25 g catalyst, 0.5 g PP_12k_, 4 bar N_2_, 240 °C, 4 h, 750 rpm.

A recyclability test consisting on five consecutive PP_12k_ depolymerizations over W_10_Zr_90_ at 240 °C with intermediate catalyst regeneration (calcination at 500 °C) disclosed nearly unchanged conversion and gasoline-range selectivity across the series (Fig. 2b, Supplementary Fig. 14, Supplementary Table 6). Closely aligned, STEM-HAADF images confirmed that the architecture of W_10_Zr_90_ remained seemingly intact after the recyclability test (Fig. 3). Incrementing the amount of processed plastic from 0.5 g to 3 g showed a high conversion degree (72%) while keeping the same selectivity pattern (Supplementary Table 7). W_10_Zr_90_ was further applied to diverse PP-based materials, including high-molecular-weight PP_250k_ (*M*_w_ = 250 kDa), centrifuge tubes, yogurt cups, and shampoo bottle caps (Fig. 2b, Supplementary Fig. 13, Supplementary Table 8). Conversion continued high (70%–93%) in all cases, with consistent gasoline selectivity (85%–88%), underscoring robustness against additives commonly presented in consumer plastics^46^. The corresponding product carbon number distributions closely overlapped (Supplementary Fig. 15), suggesting a common scission mechanism at play.

Noticing that a recently reported catalyst for polyethylene depolymerization^21^ comprised of an oxidic W–Zr phase hosted by KIT6 silica and physically mixed with HZSM-5, we synthesized and evaluate its performance for PP_12k_ depolymerization under the standard conditions used in this work (Supplementary Table 9). This catalyst yielded 20% lower conversion than W_10_Zr_90_. This result highlights the high activity of W–O–Zr ensembles and suggests that efficient processing of PP may not need additional zeolite catalytic functions. Overall, these results highlighted the high performance, versatility, and stability of W_10_Zr_90_ and triggered characterization studies pursuing the nature of active sites and associated mechanisms.

### Identification of W–O–Zr linkages as active sites

**Fig. 4:**
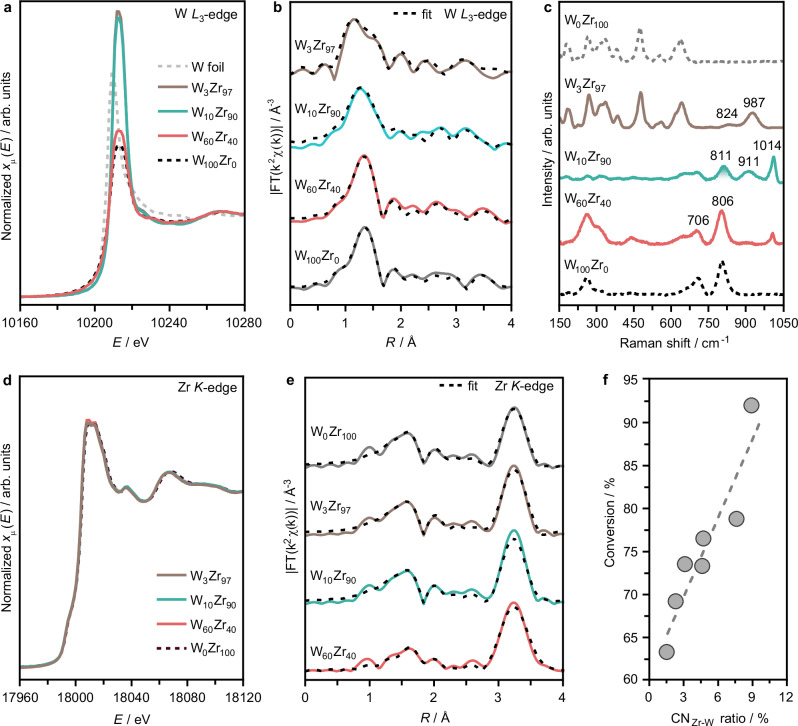
Ex-situ analysis of electronic structure and local chemical environment for representative W_a_Zr_b_ catalysts. **a** W *L*_3_-edge XANES, **b** W *L*_3_-edge EXAFS spectra and fittings in R space, **c** Raman spectra with peaks assignment as follows: 150–650 cm^−1^: ZrO_2_ vibrations, while the 700–1050 cm^−1^ region contained bands from W–O–W (504–806 cm^−1^), asymmetric (825–930 cm^−1^) and symmetric (950–1050 cm^−1^) W = O vibrations^75,76^. **d** Zr *K*-edge XANES spectra and **e** Zr *K*-edge EXAFS Fourier-transform spectra and fittings in R space. **f** Correlation between PP_12k_ conversion (defined as the C_1_–C_35_ yield) and the CN_Zr__–W_ ratio.

The distinctive architecture of the materials across the W_a_Zr_b_ family enabled clear structure-performance correlations. Starting with the analysis of electronic structure and local environments for tungsten, the W *L*_3_-edge X-ray absorption near-edge structures, XANES spectra identified fully oxidized W species in all cases^47^, consistent with the W^6+^ valence state of the W_100_Zr_0_ (WO_3_) standard sample (Fig. 4a). However, the sharper and more intense white lines in W_3_Zr_97_ and W_10_Zr_90_ indicated distinct coordination environments from distorted octahedral in WO_3_, with enhanced W = O terminal symmetry^48^. Together with STEM-HAADF (Fig. 1) and XRD (Supplementary Fig. 4) results, these data identified as predominant species isolated tungstates in W_3_Zr_97_ and three-dimensional polytungstate sub-nanoclusters in W_10_Zr_90_^42,49,50^. These sub-nanoclusters are likely bonded to ZrO_2_ defects creating a lattice slight expansion, as suggested by the shift to lower angles of the tetragonal ZrO_2_ (101) reflection as the tungsten content increased followed by reversion upon WO_3_ crystallization (Supplementary Fig. 4). Fourier-transformed *k*^2^-weighted extended X-ray absorption fine structure, EXAFS in W *L*_3_-edge, together with fitting analysis (Supplementary Figs. 16–18, Supplementary Note 3), showed that the second-shell region (3.0–3.5 Å) required both W–Zr and W–W scattering contributions (Fig. 4b, Supplementary Table 10, Supplementary Fig. 17), consistent with the coexistence of interfacial W–O–Zr bridges and W–O–W environments in the catalysts. As the W content increased, the fitted W–Zr coordination number decreased, whereas the corresponding one for W–W increased, with the local W–O paths evolving from a more tetrahedral tungstate-like environment toward a more WO_3_-like coordination, consistent with XANES results (Fig. 4c and Supplementary Note 1). Raman spectroscopy analyses of the W_a_Zr_b_ series (Fig. 4c and Supplementary Note 1) added that, for W_10_Zr_90_, ZrO_2_ signals (W_0_Zr_100_, 150–650 cm^−1^) nearly vanished, while the W = O modes red-shifted to 1014 cm^−1^ and 911 cm^−1^, consistent with aggregation into sub-nanoclusters^29^. A distinctive band at 811 cm^−1^ in W_10_Zr_90_ was attributed to W–O–Zr, consistent with the second shell W–Zr path fitted observed from W *L*_3_-edge EXAFS (Fig. 4b).

The abundance of W–O–Zr ensembles was evaluated via analysis of the Zr *K*-edge. XANES signals showed that the electronic structure and coordination environment of ZrO_2_ remained essentially unchanged across W_a_Zr_b_ catalysts, providing no evidence for the formation of zirconium tungstate or solid-solution mixed oxides (Fig. 4d). While the Zr–Zr (3.6–3.7 Å) coordination numbers, CN derived from EXAFS results (Fig. 4e, Supplementary Fig. 18, Supplementary Table 11) remained consistent with a local structure resembling bulk t-ZrO_2_ used as the fitting model (CN_Zr__–Zr1 _≅ 6.0–7.0 and CN_Zr__–Zr2 _≅ 3.5–4.0)^51,52^, the fitting Zr–W (around 3.5 Å) CN showed a volcano-like dependency with the tungsten content (Supplementary Fig. 19). Accordingly, we used the ratio CN_Zr–W_/∑CN as a semi-quantitative proxy for relative interface abundance, resulting in the sequence, W_10_Zr_90_ > W_5_Zr_95_ ≅ W_30_Zr_70_ > W_3_Zr_97_ > W_40_Zr_60_ > W_60_Zr_40_ > W_80_Zr_20_ > W_0_Zr_100_ (Supplementary Table 11), suggesting that W_10_Zr_90_ possessed the highest density of WO_*x*_–ZrO_2_ interfaces. CN_Zr–W_/∑CN correlated linearly with PP_12k_ conversion (Fig. 4f), supporting the assignment of interfacial W–O–Zr bridges as the most relevant catalytic ensembles across the catalyst series. In this context, the W content and WO_*x*_ domain size should be regarded as governing structural parameters that modulate the abundance of such interfacial sites, as shown by the mentioned volcano-type dependency of the CN_Zr–W_/∑CN ratio with tungsten content visible in the Supplementary Fig. 19.

Since W_a_Zr_b_ systems can be described as solid-acid catalysts, acidity-performance relations were explored. Temperature-programmed desorption of ammonia, NH_3_-TPD profiles and pyridine Fourier-transform infrared spectra, pyridine-FTIR analyses (Supplementary Figs. 20, 21) disclosed that, while low-W samples resemble the acidity profile of W_0_Zr_100_ and high-W samples resemble that of W_100_Zr_0_, respectively, W_10_Zr_90_ features fewer acid sites than W_0_Zr_100_ with a higher medium-strength Brønsted/Lewis ratio (Supplementary Fig. 20). Though it is not possible to draw direct correlations between their variation and performance trends^42,53^, first indications on the general type of acidity behind the excellent performance of W_10_Zr_90_ can be drawn. Pyridine FTIR results show that the reference W_10_Zr_90_ and W_0_Zr_100_ contain an almost equal distribution of Lewis and Brønsted sites, while showing stark differences in performance (Fig. 1). The only noticeable difference is the lower magnitude of a band associated to Lewis acidity at ca. 1470 cm^−1^ for W_10_Zr_90_ (Supplementary Fig. 21). The comparison with W_100_Zr_0_, exhibiting a much better performance than W_0_Zr_100_, suggests that two different Lewis acid sites (1600 cm^−1^, 1450 cm^−1^) might be related to the catalytic cycle, but the fact that these bands are also present in W_0_Zr_100_, makes this hypothesis unlikely. Regarding NH_3_-TPD profiles, it is possible to infer that strong acid sites, only present in W_0_Zr_100_, are detrimental. The reference catalyst W_10_Zr_90_ displays a broad beak covering from weak to medium strength sites. However, W_0_Zr_100_ also possesses abundant sites in this region which largely coincide with those in W_60_Zr_40_ and W_100_Zr_0_, showing all of them clearly different performance (Supplementary Fig. 20). The only relatively distinctive feature is thus the higher abundancy of sites creating the desorption peak at ca. 283°C in W_3_Zr_97_ and W_10_Zr_90_. In summary, detailed analysis of acidity suggests that effective depolymerization may require the absence of strong acid sites and that certain type of medium-strength sites more apparent on W_3_Zr_97_ and W_10_Zr_90_ (showing the largest populations of W–O–Zr ensembles) could be related to enhanced performance. However, the ex-situ character of results provided in this section recommended to investigate the dynamics of interfacial W–O–Zr structures under operation and their mechanistic role.

### Dynamics of W–O–Zr linkages under reaction conditions

**Fig. 5:**
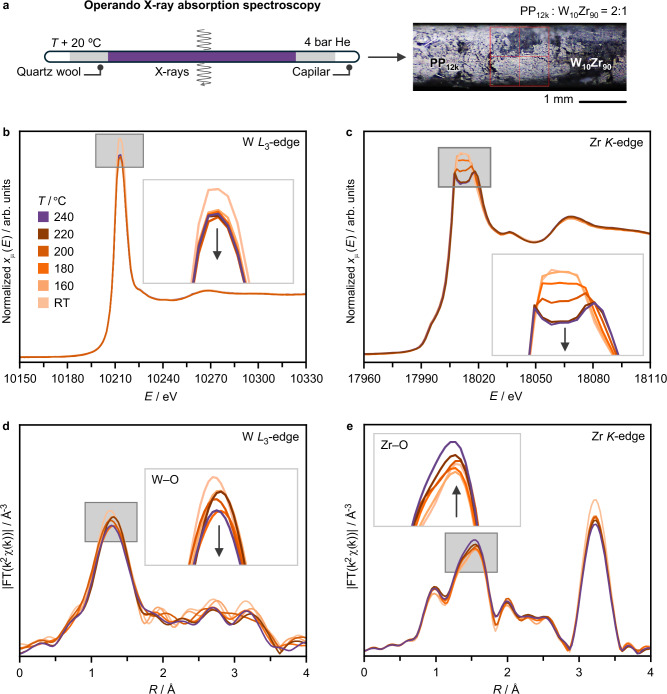
*Operando* analysis of electronic structure and local chemical environment for W_10_Zr_90_. **a** Schematic of the experimental setup for XAS analysis at different temperatures. **b** W *L*_3_-edge XANES, **c** Zr *K-*edge XANES, **d** W *L*_3_-edge EXAFS (with enlarged W–O coordination region), and **e** Zr *K*-edge EXAFS (with enlarged Zr–O coordination region). Reaction conditions: 0.25 g catalyst, 0.5 g PP_12k_, 4 bar He. RT stands for room temperature.

*Operando* XANES and EXAFS built on the relevance of W–O–Zr linkages deduced from ex-situ evidence. These techniques have been rarely applied under reaction conditions in chemical plastic recycling due to their analytical challenges related to viscous, multiphase media with strong scattering and complex background signals. In a typical experiment (Fig. 5a), PP_12k_ and the catalyst were mixed (2:1, w/w) and heated in He (4 bar) at increasing temperatures while monitoring W *L*_3_- and Zr *K*-edges (Supplementary Figs. 22, 23, Supplementary Note 3). Different from W_3_Zr_97_ (Supplementary Fig. 24) or W_60_Zr_40_ (Supplementary Figs. 25), the W *L*_3_-edge XANES spectra for W_10_Zr_90_ (Fig. 5b) showed a significant decrease in white-line intensity from room temperature to 160 °C before stabilizing, evidencing partial reduction of W species. This behavior may originate from the lower sensitivity of *operando* measurements, making that only catalysts containing abundant sub-nanocluster interfaces, such as W_10_Zr_90_, display detectable reduction degrees. These results indicate the incipient activity of the catalysts even during the temperature ramping stage and that reduction may be preferentially linked to the WO_*x*_-ZrO_2_ interface predominant in sub-nanoclusters, suggesting that W–O–Zr operates as hydrogen- or electron-accepting site.

The dynamics of these linkages could be better understood from the analysis of the Zr *K*-edge XANES of the W_a_Zr_b_ series. In contrast to W_3_Zr_97_ (Supplementary Fig. 24) and W_60_Zr_40_ (Supplementary Figs. 25), the white line intensity evolved into a distinct doublet peak (Fig. 5c) with increasing temperature, indicating substantial changes in local Zr coordination^54,55^. Complementary EXAFS analysis at the Zr *K*-edge of W_10_Zr_90_ (Figs. 5d, 5e) revealed changes in the first-shell features of both W and Zr sites. Zr *K*-edge EXAFS fitting showed that, although the total first-shell Zr–O coordination number remained nearly constant around eight, consistent with a tetragonal ZrO_2_ local structure, the shorter Zr–O_1_ subshell and the longer Zr–O_2_ subshell varied from 200 °C, suggesting interfacial oxygen reorganization (Supplementary Table 12). Additionally, the Zr–W coordination number increased above 180 °C (Supplementary Fig. 26, Supplementary Table 12). Together, these changes hint to a temperature-driven restructuring at the WO_*x*_–ZrO_2_ interface, involving interfacial oxygen redistribution and the formation of additional W–O–Zr ensembles that may originate the doublet in the Zr *K*-edge (Fig. 5c). Of note, the emergence of new W–O–Zr linkages above 180 °C in W_10_Zr_90_ parallelized the steep increase of catalytic performance at 180 °C (Fig. 2b). O*perando* evidence thus reinstated that in-situ generated W–O–Zr structures at WO_*x*_–ZrO_2_ interfaces operate as active sites.

### Main mechanistic features of polypropylene depolymerization

**Fig. 6:**
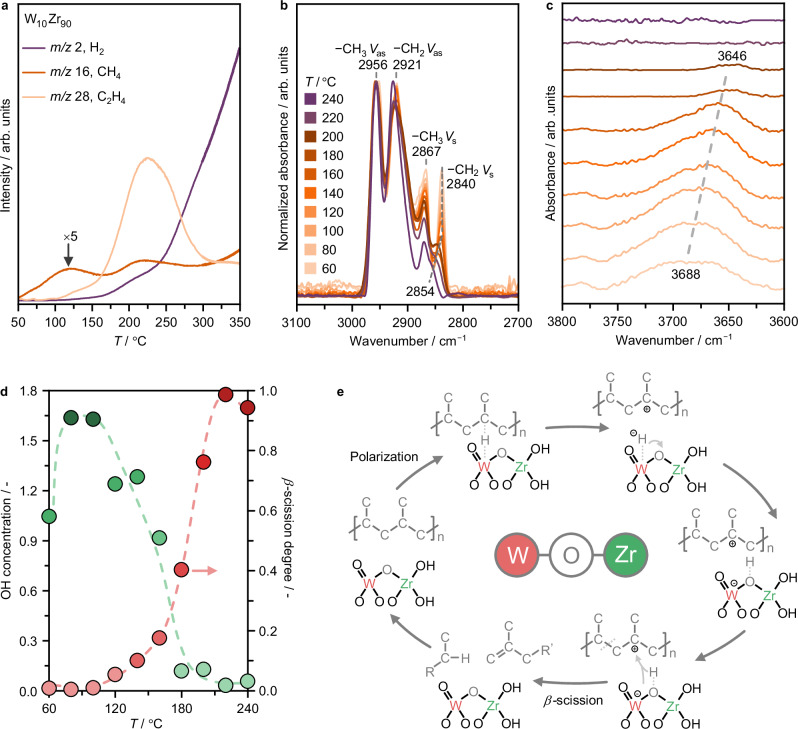
Identification of key mechanistic features for the depolymerization of PP over W_10_Zr_90_. **a** TPSR-MS profiles, *operando* DRIFTS evolution of **b** PP C–H stretching, and **c** OH stretching. **d** Correlation between OH concentration (from integrated DRIFTS band at 3600–3770 cm^−1^) and normalized C_2_H_4_ MS intensity (*m/z* 28) for the depolymerization of PP_12k_ over W_10_Zr_90_. **e** Proposed main reaction pathway.

An analysis based on electronegativity differences between W (1.7) and Zr (1.4) already suggests that in the W–O–Zr moiety a displacement of the electronic density toward the W center may exist. The bridging oxygen would thus carry a less negative average charge, leading to milder acidity than the corresponding site in homonuclear materials. This aligns with the prevalence of acid sites with medium strength in W_10_Zr_90_ observed in Supplementary Fig. 20 and hints to distinct interactions with the PP molecule than W–O–W and Zr–O–Zr equivalents. This reasoning complements initial insights on the operation of W–O–Zr ensembles from temperature-programmed surface reaction coupled with online mass spectrometry, TPSR-MS (Fig. 6a and Supplementary Figs. 27, 28) focused on *m/z* 16 (CH_4_, terminal demethylation), *m/z* 28 (C_2_H_4_, intrachain *β*-scission), and *m/z* 2 (H_2_, subsequent proton transfer). For W_10_Zr_90_, CH_4_ became detectable at ca. 60 °C and reached a first maximum at around 120 °C, well below the PP_12k_ melting point. Across the W_a_Zr_b_ series, W_0_Zr_100_ showed the strongest evolution of CH_4_ at low temperatures with a gradual decrease with increasing W content (Supplementary Figs. 27, 28). This suggests ZrO_2_ sites as the origin of low-temperature CH_4_ formation. The signal for C_2_H_4_ initiated at ca. 120 °C over W_10_Zr_90_ and rose sharply upon polymer melting, peaking at 220 °C–240 °C. H_2_ evolution started near the melting point and intensified after the C_2_H_4_ maximum, supporting a hydrogen-transfer mechanism, consistent with *operando* EXAFS. A two-step pathway could be thus deduced: (i) low-temperature chain-end activation by ZrO_2_ sites, followed by (ii) intrachain *β*-scission at adjacent W–O–Zr ensembles (Supplementary Fig. 29).

*Operando* diffuse reflectance infrared Fourier transform spectroscopy, DRIFTS analysis at different temperatures over W_10_Zr_90_ showed comparable intensities of methyl (–CH_3_) and methylene (–CH_2_–) vibrations (2600–3200 cm^−1^) at 60 °C, as expected from unreacted PP (Fig. 6b)^24,56,57^. With increasing temperature, –CH_2_– backbone bands decreased substantially faster than –CH_3_ side-chain/terminal bands, evidencing a predominant *β*-scission pathway. Close to the PP_12k_ melting point (157 °C), –CH_2_– bands exhibited a pronounced blue shift, while the OH stretching band of W_10_Zr_90_ simultaneously red shifted with increasing temperature (Fig. 6c). These evolutions suggest strong interactions between the polymer backbone and surface OH groups on W_10_Zr_90_^24^. The dynamics and lack of shift in parallel analyses for other W_a_Zr_b_ catalysts allowed to conclude that OH groups on W_10_Zr_90_ are highly active toward *β*-scission (Supplementary Figs. 30, 31, Supplementary Note 2). Furthermore, the OH peak area from *operando* DRIFTS could be correlated across temperatures with the normalized C_2_H_4_ signal intensity from TPSR-MS (Fig. 6d). In W_10_Zr_90_, the concentration of OH species generated in-situ prior to the onset of *β*-scission started decreasing at ca. 120 °C, yielding a solid negative exponential correlation (Supplementary Fig. 33) and supporting the promotion of *β*-scission by OH groups. In contrast, the low activity of W_0_Zr_100_ (Fig. 2a) with high concentration of BAS and OH groups (Supplementary Figs. 20,32) introduced the unique character of OH groups present in W_10_Zr_90_.

Furthermore, *operando* DRIFTS results are compatible with a W center first polarizing a C–H bond of the polymer chain, inducing the heterolytic cleavage^36,58,59^, in line with the decrease in the *operando* W *L*_3_-edge XANES white line (Fig. 5b). In this sequence, W may be partially reduced by accepting an electron, while the abstracted hydrogen atom is transferred to the neighboring bridging oxygen (spillover-like)^60,61^, concomitantly generating a carbenium ion intermediate (Fig. 6e). Another supporting observation is the blue color acquired by the catalyst after reaction, consistent with the formation of tungsten bronze-type species (H*ₓ*WO_3_/H*ₓ*WO*ₓ*, Supplementary Fig. 34). The decrease in OH intensity with temperature is most plausibly explained by faster hydrogen/electron reinsertion at the *β*-carbon, thereby completing the scission cycle and regenerating the active site (Fig. 6e). Accordingly, OH bands became barely detectable at higher temperatures (220 –240 °C) while the absence of inverted features indicated a balanced formation and consumption of OH under a rapid turnover. These W–O–Zr linkages may thus function as transient electron/hydrogen-accepting centers driving selective C–C bond scission. Of note, the same investigations applied to W_3_Zr_97_, W_60_Zr_40_, and W_100_Zr_0_ excluded terminal W = O and W–O–W ensembles as relevant hydrogen transfer centers (Supplementary Figs. 30–32). The returned hydrogen inserts at one end of the cleaved chain to form a new alkane, while the other end yields a new olefin, thus completing a *β*-scission event. Control experiments in pressurized H_2_ at identical conditions showed no change in either activity or product distribution, excluding molecular hydrogen participation and further confirming the internal hydrogen transfer mechanism (Supplementary Table 13). As suggested for non-catalytic pyrolysis, the high fraction of alkanes in the product distribution could be related to the reaction of olefins with molecular H_2_^34^. In this case, hydrogen may originate from dehydrogenation of long fragments, as suggested by the decreasing H:C ratio in the solid residue (Supplementary Fig. 11) and the detection of H_2_ in TPSR experiments (Fig. 6a) and catalytic evaluations (Supplementary Fig. 12).

Together, these investigations converge into a coherent description of the polypropylene scission mechanism on tungstated zirconia catalysts, identifying dynamically regenerated W–O–Zr ensembles as the dominant active sites. The main features (Fig. 6e) can be described as: (i) C–H bond polarization and heterolytic cleavage: Formation of a carbenium ion intermediate by the W center. W is partially reduced by electron transfer, while the hydrogen atom generates a OH species; (ii) *β*-scission with hydrogen/electron reinsertion: The abstracted hydrogen atom is transferred back from the W–O–Zr linkage to the carbenium fragment, quenching it to form a new alkane and an olefinic species. This concerted return reestablishes the W–O–Zr site and closes the catalytic cycle. Of note, additional experiments over W_10_Zr_90_ using HDPE as a feedstock yielded low conversion (24%, Supplementary Table 14). This result strongly suggests that the presence of tertiary and likely primary carbon atoms in PP is a relevant structural feature for the efficient operation of W–O–Zr ensembles and that catalyst design efforts may target specific polyolefins to attaining high efficiency.

### Environmental and economic comparison of technologies

**Fig. 7:**
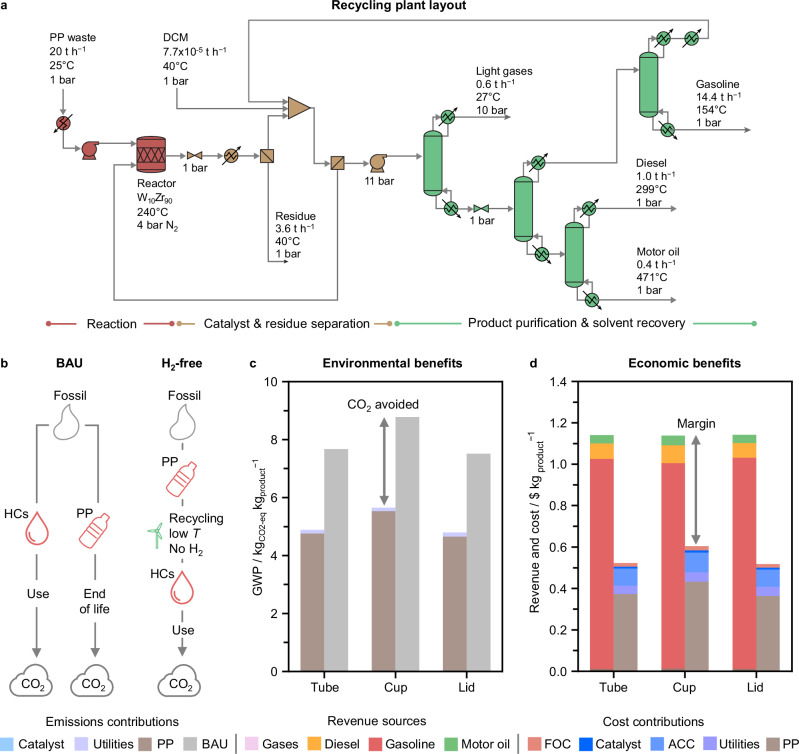
Analysis of environmental and economic performance for the depolymerization of PP-based consumer goods over W_10_Zr_90_. **a** Simplified process flowsheet for the chemical recycling process considering catalytic performance provided in Supplementary Table 8. The values in the figure correspond to the shampoo lids scenario. **b** General schemes of life cycles for the hydrogen-free depolymerization of polyolefins and for the business-as-usual, BAU production of polyolefins and liquid hydrocarbons, HCs. **c** Global warming potential versus equivalent business-as-usual fossil production and **d** production cost and revenues from product selling at market prices, with margin indication of their respective product portfolio. Detailed breakdowns and system boundaries are available in Supplementary Fig. 38 and Supplementary Fig. 39, respectively. DCM stands for dichloromethane, FOC stands for fixed operating costs and ACC for annualized capital cost.

Life cycle and technoeconomic analyses brought positive environmental and economic prospects for the hydrogen-free PP chemical recycling strategy over W_10_Zr_90_. We simulated an industrial plant processing 20 t h^−1^ of consumer goods (centrifuge tubes, yogurt cups, shampoo lids, catalytic performance in Fig. 2b) integrating the reaction stage with additional units for stream conditioning, catalyst regeneration, and product separation (Fig. 7a, detailed information of modeling in Supplementary Fig. 35 and Methods section). The environmental impact was assessed using the global warming potential, GWP indicator over the full life cycle.

The business-as-usual, BAU scenario reflects the current situation, where PP-based consumer goods are produced from fossil feedstocks and, at the end of life, managed through conventional processes (34% landfilling and 66% incineration), while fuels are also obtained from fossil resources in a refinery (Fig. 7b). The hydrogen-free recycling route leads to an average 35% reduction in GWP compared to the BAU scenario (Fig. 7c and Supplementary Figs. 36–38). Depolymerization of all consumer goods show similar performance, with combined propylene production, collection, sorting, and transport being the dominant GWP contributor (ca. 80%), in which the production stage via fossil-based energy-intensive processes, most notably the steam cracking of naphtha, contributing with the largest share (Supplementary Fig. 38). The economic analysis indicates a favorable outlook (Fig. 7d), with profit margins of 0.5–0.7 $ per kg of product when comparing production costs and expected product revenues at market prices of the product portfolio. This result is driven by high yields toward liquid hydrocarbons, mild reaction conditions, and the use of an inexpensive catalyst. The largest cost contribution stems from the acquisition of waste PP-based items (70%), which is traded as a commodity requiring collection and processing prior to sale. A sensitivity assessment predicted that, even under unfavorable raw materials and energy prices, market fluctuations, and considering life cycle inventory data uncertainty, the process exhibits consistent profits and CO_2_ savings across (Supplementary Fig. 38).

**Fig. 8:**
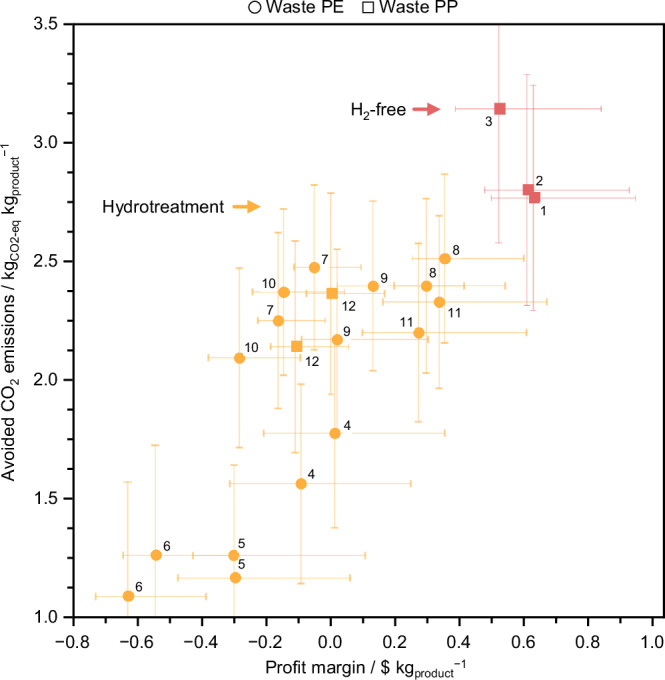
Environmental and economic performance map for polyolefin chemical recycling technologies at mild conditions. Hydrotreatment and hydrogen-free routes are compared in terms of profit margin ($ kg_product_^−1^) and net GWP reduction over the BAU system (kg_CO2-eq_ kg_product_^−1^). Number labels, performance, and catalytic materials for each report are provided in Supplementary Tables 22, 23. The uncertainty zone for each point (vertical and horizontal lines) is computed considering uncertainty in fuel and material prices as described in Supplementary Table 18 and a ±25% GWP impact change in the chemical recycling routes. The hydrotreatment routes are evaluated using green hydrogen produced with electricity from either solar PV or offshore wind.

This framework was extended to other polyolefin chemical recycling technologies requiring similar temperatures, inspired by recent works generalizing sustainability analysis of hydrotreatments^62^. The analysis included representative hydrotreatment reports including hydrogenolysis and hydrocracking (Supplementary Fig. 39) addressing consumer goods with detailed product chain length ranges (plant layout in Supplementary Fig. 40). This strategy enabled a harmonized comparison including uncertainty analysis depicted in Fig. 8 as a technology sustainability map. Overall, chemical recycling of polyolefins is found to be sustainable in both the environmental and economic dimensions with nuances even under less favorable scenarios. Hydrogen-free processing of PP-based consumer goods over W_10_Zr_90_ sits at the forefront zone. As for hydrotreatments, reports form a distinct and broader performance region due to their dependence on green hydrogen (sourced from wind and solar water splitting in our analysis), frequent use of noble-metal catalysts in hydrogenolysis (e.g., Ru, Pt), and still mild yields of highly valuable lubricant hydrocarbons. Uncertainty bars account for raw materials and energy prices, market fluctuations, and life cycle inventory data ranges, making apparent the large variability that sustainability performance is subject to and highlighting the relative comparison across technologies as the main result of this analysis. All in all, chemical recycling routes of polyolefin waste offer a marked reduction in greenhouse gas emissions compared to conventional end-of-life options, while generating product portfolios that, depending on the specific technology and its performance, may offer favorable economic prospects.

## Discussion

This work establishes a hydrogen- and solvent-free catalytic route for polypropylene depolymerization at mild temperatures. Flame-synthesized tungstated zirconia catalysts unify high activity and selectivity toward gasoline hydrocarbons (>80% yield) with well-defined structures, enabling identification of W–O–Zr ensembles as active sites. *Operando* spectroscopies reveal the formation of these interfacial linkages under reaction and their operation as transient electron–hydrogen accepting sites that activate C–H bonds and guide selective C–C scission through internal hydrogen transfer. Process modeling, life cycle assessment, and a harmonized comparison across chemical recycling technologies including hydrotreatments show that this pathway already sits at the forefront of environmental and economic performance. By linking molecular-level catalyst design to validated system-scale benefits, these findings suggest interfacial M–O–M′ ensembles (M = metal) as a design platform for advancing catalytic polyolefin waste management.

## Methods

### Catalyst synthesis

Tungstated zirconia catalysts with variable composition (denoted as W_a_Zr_b_, where a and b represent the mol% of tungsten and zirconium, respectively, listed in Supplementary Table 1) for the recycling of polypropylene was synthesized via flame spray pyrolysis (FSP) using 0.10 mol kg^−1^ stock solutions of tungsten hexacarbonyl (W(CO)_6_, Acros; 99%) and zirconium(IV) 2-ethylhexanoate (Zr(2-ethylhexanoate)_4_, abcr; 97%) in tetrahydrofuran (THF, Sigma-Aldrich, ≥99.9%) as precursors, by mixing the stock solutions in the desired ratios and sonicating for 30 min. The FSP setup has been described elsewhere in detail^63^. In brief, the liquid metal precursor was fed through a spray nozzle (0.4 mm) by a micro-annular gear pump (HNPM; mzr-2900) at a rate of 10.0 cm^3 ^min^−1^. The spray was dispersed by oxygen (5.0 dm^3^ min^−1^; 1.5 bar overpressure) and ignited by a premixed pilot flame of CH_4_ (1.2 dm^3^ min^−1^) and O_2_ (2.4 dm^3^ min^−1^) surrounding the nozzle reaching 2000 °C–3000 °C based on adiabatic calculations^63^. The gas flow through the reactor was maintained at 10–20 m^3^ h^−1^ by a vacuum pump (Mink MV 0040 D; Busch) using ambient air passing through a HEPA filter. The obtained catalyst particles were separated from the gas stream using glass fiber filters (Hahnemühle, GF6, diameter 257 mm).

The WZr-KIT6-HZSM-5 catalyst was synthesized following a previously reported method^24^. Briefly, 3.7 g of Pluronic P123 (Sigma Aldrich; *M*_n_ ~ 5800 Da), 6.8 g of hydrochloric acid solution (Sigma Aldrich; 37 wt%), and 3.7 g of *n*-butanol (VWR international AG; 99.5%) were dissolved in 130 g of deionized water at 35 °C. The mixture was stirred for 1 h to ensure complete dissolution. Subsequently, 8.0 g of tetraethyl orthosilicate (TEOS; Sigma Aldrich; 98%), 1.24 g of zirconyl chloride octahydrate (Sigma Aldrich; 98%), and 0.95 g of ammonium metatungstate (ABCR; 99.9%) were added. The resulting mixture was stirred at 35 °C for at least 24 h, after which it was transferred into four 50 mL stainless steel autoclaves and subjected to hydrothermal treatment at 100 °C for 24 h. The solid product was then recovered by filtration, dried at 100 °C, and finally calcined in air at 550 °C for 5 h. HZSM-5 was prepared from calcination of ammonium ZSM-5 (Zeolyst International) in air at 550 °C for 10 h and then physically mixed with WZr-KIT6 in a four to one mass ratio, respectively.

### Ex-situ catalyst characterization

X-ray fluorescence, XRF spectroscopy was performed using a Rigaku ZSX Primus IV spectrometer equipped with a 4 kW Rh source and LiF (200), Ge, PET, and RX26 analyzing crystal detectors.

Powder X-ray diffraction, XRD measurements were conducted in a Rigaku SmartLab diffractometer, using Cu K*α* radiation (*λ* = 1.541 Å). The data was recorded in the 2*θ* range of 10°–90° with an angular step size of 0.025° and a counting time of 1.5 s per step.

Temperature-programmed surface reaction, TPSR experiments were performed in a Micromeritics Autochem II 2920 unit equipped with a thermal conductivity detector, TCD and a Pfeiffer Vacuum Omni Star mass spectrometer, MS. The catalyst (90 mg) was thoroughly mixed with polypropylene with weight average molecular weight, *M*_w_ ~ 12 kDa, (10 mg, Sigma-Aldrich), denoted as PP_12k_, and loaded into a U-shaped quartz micro-reactor followed by drying in He (40 °C, 1 h). The sample was subsequently heated in flowing He (20 cm^3 ^min^−1^) to 600 °C at a rate of 5 °C min^−1^.

Temperature-programmed desorption of ammonia, NH_3_-TPD was performed in a Micromeritics Autochem II 2920 unit equipped with a thermal conductivity detector and online MS. Prior to NH_3_-TPD measurements, samples (100 mg) were loaded into a quartz tube, dried under flowing He at 150 °C for 1 h (20 cm^3 ^min^−1^, heating rate 10 °C min^−1^), and then cooled down to 40 °C (20 °C min^−1^). After 10 min, the sample was exposed to flowing NH_3_ in He (10 vol%, 20 cm^3 ^min^−1^) for 1 h. The initial desorption of weakly bound NH_3_ was conducted at 60 °C under flowing He (20 cm^3 ^min^−1^) for 20 min. Temperature-programmed desorption was then performed under flowing He (20 cm^3 ^min^−1^) in the 40 °C–800 °C range (10 °C min^−1^), with NH_3_ evolution monitored by a TCD and MS.

Raman spectroscopy was performed in a Horiba LabRAM HR Evolution UV–vis–NIR confocal Raman system using a Cobolt Samba Nd/YAG laser with a wavelength of 532 nm, a power of 5.7 mW and a 50 × Olympus LMPlanFLN objective. Spectra were collected with an acquisition time of 10 s and an accumulation number of 5.

Pyridine Fourier-transform infrared spectra were recorded using a Bruker Optics Vertex 70 spectrometer. For each measurement, the catalyst (200 mg) was pressed into a thin wafer and placed in a custom-made quartz reaction cell with KBr windows, connected to a molecular pump and capable of in situ heating. The sample was evacuated at 300 °C under vacuum for 120 min, cooled to 50 °C, and exposed to pyridine for 10 min. After evacuation for 30 min to remove physisorbed species, IR spectra were collected at 50 °C intervals in the 150 °C–350 °C range.

High-angle annular dark-field scanning transmission electron microscopy, HAADF-STEM and energy-dispersive X-ray spectroscopy, EDX data were acquired using an aberration-corrected JEM-ARM300F microscope (GrandARM, JEOL) operated at 300 kV or a Talos F200X (Thermo Fisher Scientific).

Ex-situ X-ray absorption spectroscopy, XAS was conducted at the Swiss Norwegian beamline, SNBL, BM31 of the European Synchrotron Radiation Facility (ESRF)^64^. The X-ray beam was collimated using a double-crystal liquid N_2_ cooled Si (111) monochromator. For the absolute energy calibration for XAS measurements, a W foil and a Zr foil were measured using N_2_-filled ionization chambers for optimal absorption levels. Continuous scanning was performed for both W-*L*_3_ and Zr-*K* edges at a step size of 0.5 eV. The beam size was set to 3 × 0.2 mm (horizontally×vertically). The resulting spectra were energy-calibrated, background-corrected and normalized using the Athena program from the Demeter software suite. *k*^2^-weighted extended X-ray absorption fine structure, EXAFS spectra were fitted in the optimal *k*- and *R*-windows using the Artemis program.

### *Operando* catalyst characterization

*Operando* diffuse reflectance infrared Fourier transform spectroscopy, DRIFTS was applied using a Bruker Invenio S spectrometer following this procedure: (i) the catalyst was mixed with PP_12k_ in a 1:2 mass ratio and loaded in the cell, (ii) the sample was flushed with He and pressurized to 4 bar, (iii) background data was collected once the pressure and signal had stabilized, and measurements were performed starting at 60 °C, increasing by 20 °C every 0.5 h until reaching 240 °C, which was then maintained for 2 h; (iv) the system was naturally cooled down to room temperature. Spectra were recorded at 0.5 min intervals with a resolution of 4 cm^−1^.

*Operando* XAS experiments were conducted at the same beamline of BM31, SNBL, ESRF. The X-ray beam was collimated using a double-crystal liquid N_2_ cooled Si (111) monochromator^64^. For the absolute energy calibration for XAS measurements, a W foil and a Zr foil were measured, using N_2_-filled ionization chambers for optimal absorption levels. Continuous scanning was performed for both W *L*_3_- and Zr *K*-edges at a step size of 0.5 eV. The beam size was set to 3 × 0.2 mm (horizontally × vertically). The catalyst and the plastic were placed between two plugs of quartz wool in a quartz capillary reactor cell (1.5 mm outer diameter, 0.01 mm wall thickness). The experiment procedure was as follows: (i) the catalyst was mixed with PP_12k_ in a 1:2 mass ratio and loaded in the cell; (ii) the sample was flushed with He and pressurized to 4 bar; (iii) data collection was initiated at room temperature, where consecutive spectra were acquired five times at both the W *L*_3_-edge and Zr *K*-edge; (iv) the temperature was then increased to 160 °C, followed by stepwise heating in 20 °C increments to 240 °C. At each target temperature, the system was stabilized for 30 min, and consecutive spectra were again collected five times at both the W *L*_3_-edge and Zr *K*-edge. The high-purity gases were introduced by a set of Bronkhorst digital mass flow controllers, and the outcome was monitored on-line via a Pfeiffer Vacuum Omni Star GSD 320O MS with QUADERA software. The resulting spectrum was energy-calibrated, background-corrected and normalized using the Athena program from the Demeter software suite. *k*^2^-weighted EXAFS spectra were fitted in the optimal *k*- and *R*-windows using the Artemis program.

### Catalyst evaluation

Catalytic depolymerization of polypropylene under hydrogen-free conditions was performed in a parallel pressurized batch reactor setup (BuchiGlasUster, Switzerland) consisting of three 50 cm^3^ (110 cm^3^ for gas volume) stainless steel reactors. Each reactor was equipped with an electrical heating jacket, active cooling unit (chilled water system), mechanical stirring, temperature/pressure control systems and gas sampling lines. Typically, plastics (0.5 g) including virgin polypropylene PP_12k_ (also used for mechanistic studies due to its lower molten viscosity amenable to FTIR and TPSR operation), PP_250k_ (*M*_w_ = 250 kDa, Sigma Aldrich), HDPE_100k_ (*M*_w_ = 100 kDa, Sigma Aldrich) and consumer goods based on PP (centrifuge tube, yogurt bottle, and shampoo lid; washed and cut into pieces before using with catalysts (0.25 g) were added to a glass inset and the combined weight was recorded, *m*_before_. The inset was then placed inside the reactor. Prior to starting the experiment, the reactor was flushed with N_2_ before being pressurized to the desired pressure. The reaction parameters and individual steps were programmed into the Systag Flexsys software before starting the experiment. Temperature, pressure and stirring torque were recorded during the reaction. The reaction mixture was heated and mechanically stirred for a set reaction time after having reached the set temperature (4 bar, 240 °C, 750 rpm and 4 h, respectively, unless otherwise specified). PID control parameters were chosen to avoid temperature overshooting. This implied slightly different temperature ramping processes across different conditions, averaging 3 °C min^–1^_._ After reaction, the reactor was cooled to ambient temperature with circulating chilled water.

### Product analysis

Gaseous products were collected from the headspace of the reactor by connecting a sampling cylinder and analyzed using a gas chromatographer Agilent 8860 GC System equipped with an Agilent J&W PoraPlot Q column (25 m × 0.53 mm × 20 μm) and a flame ionizing detector, FID. H_2_ was used as the carrier gas and a heating ramp (35 °C–300 °C, 5 °C min^−1^) was applied while the inlet and FID were held at fixed temperatures of 300 °C and 200 °C, respectively. GC FID calibration for C_1_ to C_6_ products was performed using a standard refinery mixture (Agilent P/N: 5080-8755, Agilent Technologies Inc.). The glass inset was removed from the reactor and weighed (*m*_after_) to determine the total amount of gas (*m*_gas_) formed with Eq. 1.

1 $${m}_{{{{\rm{gas}}}}}={m}_{{{{\rm{before}}}}}-{m}_{{{{\rm{after}}}}}$$

The liquid and solid products remaining inside the inset were dissolved in dichloromethane (10 cm^3^), sonicated (40 °C, 30 min) and filtered using a syringe. The weight of filter and syringe were measured before and after their use in completely dry conditions, with the difference in mass corresponding to the amount of solid residue, *m*_residue_. The amount of liquid products, *m*_liquid_ was calculated with Eq. 2 by subtracting the gas product and solid residue from the amount of substrate, *m*_feedstock_ used. This procedure operates on the assumption of no loss of material during operation and thus on a closed carbon balance.

2 $${m}_{{{{\rm{liquid}}}}}={m}_{{{{\rm{feedstock}}}}}-{m}_{{{{\rm{gas}}}}}-{m}_{{{{\rm{residue}}}}}$$

The filtered liquid samples were analyzed in an HP Agilent 6890 GC (Agilent Technologies, Inc., USA) equipped with an HP DB-5 HT column (15 m × 0.25 mm × 0.10 μm) to determine the distribution of products. A heating ramp was applied (40 °C–375 °C, 4 °C min^−1^), while the FID was held at a fixed temperature of 340 °C. Initial and final hold times were set at 2 and 10 min, respectively. GC FID calibration was performed using a mixture of self-prepared alkanes solution with carbon numbers of 6, 7, 8, 10, 12, 16, 20.

The yields expressed as percentage of gas phase, *Y*_gas_ and liquid, *Y*_liquid_ products were calculated with Eqs. 3 and 4:

3 $${Y}_{{{{\rm{gas}}}}}=\frac{{m}_{{{{\rm{gas}}}}}}{{m}_{{{{\rm{feedstock}}}}}}\cdot 100$$

4 $${Y}_{{{{\rm{liquid}}}}}=\frac{{m}_{{{{\rm{liquid}}}}}}{{m}_{{{{\rm{feedstock}}}}}}\cdot 100$$

The overall conversion, *X* was calculated according to Eq. 5:

5 $$X={Y}_{{{{\rm{gas}}}}}+{Y}_{{{{\rm{liquid}}}}}$$

The yield expressed in percentage for each carbon range, *Y*_*C*_ was calculated with Eq. 6:

6 $${Y}_{C}=\frac{{\sum }_{n}^{j}[{C}_{i}]\cdot Y}{{\sum }_{1}^{36}[{C}_{i}]}\cdot 100$$

where *Y*_*c*_ is the yield of hydrocarbons with a carbon-number range from *i* to *j*; [*C*_*i*_] is the concentration of the *i*^th^ hydrocarbon; *Y* is the overall yield towards gas or liquid products, respectively.

The gasoline range selectivity expressed in percentage was calculated with Eq. 7:

7 $${S}_{{{{\rm{gasoline}}}}}=\frac{{\sum }_{4}^{12}[{Y}_{c}]}{X}\cdot 100$$

where *S*_gasoline_ is the selectivity of gasoline with the carbon-number range from 4 to12.

For proton nuclear magnetic resonance, ^1^H NMR spectroscopy analysis of the liquid products, the sample was dried at room temperature using a rotary evaporator to remove the dichloromethane solvent and then dissolved in dichloromethane-d_2_ to a concentration of 100 mg mL^−1^. The ^1^H NMR spectra were recorded on a 500 MHz Bruker Ultrashield spectrometer.

The H and C contents of the solid residues were determined using a LECO TrueSpec Micro elemental analyzer. Samples were dried under vacuum at 80 °C overnight before measurement. Typically, the sample (~1 mg) was combusted in pure O_2_ at ~950 °C, and the evolved gases were quantified by infrared spectrometry (C and H) detection. The instrument was calibrated with acetanilide, and each value represents the average of at least three replicates.

### Process modeling of polypropylene chemical recycling

Polypropylene chemical recycling was assessed based on experimental conversion and selectivity obtained using the W_10_Zr_90_ catalyst. A process model was developed in Aspen Plus v15 using the Peng-Robinson property method, suitable for non- or mildly polar mixtures containing hydrocarbons and light gases. Waste PP was modeled as a polyolefin with a molecular weight of 250 kDa and an average input flow of 20 t h^−1^, following assumptions from similar works ^62,65^.

The process flowsheet, shown in Supplementary Fig. 35, considers the reactor stage as well as detailed downstream separation blocks. PP (25 °C, 1.013 bar) is fed to the process, heated (240 °C) in H1, and pumped (4 bar) in P1 to reaction conditions. The reactor (R1) was modeled as a RYield reactor block operating at 240 °C and 4 bar, with the tungstated zirconia catalyst enabling the conversion and selectivity described in Supplementary Table 8 for the same conditions. The outlet stream is first cooled to 40 °C (C1) and depressurized to 1.013 bar (V1) before passing through a decanter centrifuge that removes the solid residue. The liquid is then mixed with dichloromethane (DCM) and filtered, thereby regenerating the catalyst, which is recycled to reactor R1. It is assumed that total catalyst replacement occurs after 1000 h of operation. The resulting stream, containing the product mixture and solvent, is sent to the downstream separation section. The four main products (i.e., light gases, gasoline, diesel, and motor oil) are purified to high molar purity in four distillation columns (D1–D4) modeled with the RADFRAC rigorous model. The dichloromethane, DCM solvent is recovered in the condenser of column D3, recycled to mixer M1, and then returned to the catalyst regeneration unit. To compensate for process losses, particularly during product recovery, a DCM make-up stream is required. Finally, heat integration was performed in Aspen Energy Analyzer v15 to design a heat exchanger network that optimally integrates the hot and cold process streams to reduce utilities consumption.

### Technoeconomic and life cycle assessment

Mass and energy balance data were obtained from the corresponding simulation to calculate costs and build life cycle inventories, LCI^66^. The technoeconomic assessment, TEA determines the total annualized cost, TAC of producing the end-product portfolio and was calculated using the cost factors and correlations from Towler and Sinnott^67^. The TAC is calculated as a function of the variable operating cost, VOC, the fixed operating cost, FOC, and the annualized capital cost, ACC.

The ACC and FOC were also derived using cost correlations from Towler and Sinnott^67^. The general assumptions of the plant are described in Supplementary Table 15. The values used to estimate capital costs are provided in Supplementary Table 16 for each equipment type. The inside battery limits cost, ISBL is calculated based on the purchasing cost, C and typical installation factors for ‘fluids-solids’ processes^67^. The ISBL is scaled considering additional costs (offsite, engineering, contingencies) to obtain the total fixed capital cost, FCC. This term is then annualized to provide the annual capital charge ratio, accounting for accumulated interest over the plant’s lifetime.

The FOC is determined from the FCC and the cost of labor, CL. It comprises expenses linked to operating labor, supervision, salary overheads, and maintenance, among others^67^. The CL corresponds to five operators (three shift positions plus two operators for the catalyst regeneration and residue section) with an average yearly salary of USD 60'000 per person. All TEA values are expressed in USD 2024. Cost data were adjusted to the selected year using the Chemical Engineering Plant Cost Index, CEPCI when not directly available. The cost parameters used in the VOC are shown in Supplementary Table 17, while the market prices considered for the revenues are presented in Supplementary Table 18.

The LCA followed the methodology described in the ISO 14040/14044^68,69^ standards with the Ecoinvent 3.10 database^70^ as background system and the outputs of the process simulations as foreground data. The global warming potential, GWP of the different scenarios was calculated with the IPCC 2021 method for climate change. The calculations were carried out in Brightway v2.4.6^71^. The assessment aims to evaluate the cradle-to-grave life cycle impacts of obtaining one kilogram of end product portfolio (i.e., mix of light gases, gasoline, diesel, and motor-oil). For the chemical recycling scenario, the LCI considers: (i) the complete life cycle of PP, including the extraction of raw materials, the PP production steps, the use phase of PP, and final waste collection and sorting; and (ii) the extraction of raw materials and production of materials/energy vectors needed in the hydrogen-free chemical recycling. The business-as-usual, BAU scenario consists of an equivalent multi-product system which accounts for the fossil-based production of the respective hydrocarbon products, as well as the production and end-of-life treatment of the corresponding PP (assuming that 66% of the waste polymer is incinerated and 34% landfilled)^72^. The use of fuels is excluded from the assessment, as combustion-related emissions are identical in both scenarios. Supplementary Tables 17–20 contain the LCI data used in the LCA calculations. The contributions of processes along the chemical recycling supply chain for shampoo lids and BAU pathways are shown in Supplementary Figs 36, 37. The cradle-to-grave system boundaries considered for the circular waste plastic recycling and fossil-based BAU alternatives are displayed in Supplementary Fig. 39.

Additionally, an uncertainty assessment was performed for the chemical recycling and BAU scenarios. To this end, we applied Monte Carlo sampling with 500 iterations, based on the Ecoinvent pedigree matrix, which provides uncertainty levels for the life cycle inventory parameters. Uncertainties affecting economic performance were modelled using minimum and maximum values from the literature, as detailed in Supplementary Tables 17, 18. Results for all waste PP sources, including uncertainties in cost and GHG emissions, are shown in Supplementary Fig. 38.

### Comparison across polyolefin chemical recycling technologies

The technoeconomic and environmental framework was adapted to assess alternative chemical recycling processes. A process simulation capable of handling different experimental datasets was carried out for the hydrotreatment technologies applied to waste plastic valorization (Supplementary Fig. 40). The mass yields used were taken from representative reports (Supplementary Table 22). This analysis was performed to identify the environmental and economic outlook of recycling waste plastics using the hydrogen-free depolymerization of polypropylene over W_10_Zr_90_ and other catalytic approaches, thereby helping to determine the best strategies for different plastic waste streams.

The assessment follows the same standardized procedure described for the hydrogen-free pathway. Conversion data from each experiment were used in the process simulations to determine the mass and energy requirements for the TEA and LCA. The operating conditions from the experimental work (e.g., temperature, pressure, catalyst-to-plastic ratio) were considered for each simulation. The catalysts were modelled according to the descriptions provided in the original studies, as summarized in Supplementary Table 23. Due to scarcity of performance stability studies, a uniform lifetime of 1000 h of operation was assumed for all technologies and scenarios.

The TAC and GWP values were calculated following the established TEA and LCA methodology. The profit margin was then determined as the difference between the levelized TAC and the potential revenues from selling the products of the chemical recycling plant. The net GWP reduction is defined as the difference between the cradle-to-grave GWP of the chemical recycling route and its analogous fossil BAU system, corresponding to the conventional refinery-based production emissions (cradle-to-gate) of the same product portfolio plus the cradle-to-grave emissions from the production and end-of-life of the plastic.

## Supplementary information

Supplementary Information

Transparent Peer Review file

## Data Availability

Additional datasets generated in this study are provided in the Supplementary Information and at the Zenodo repository [https://zenodo.org/records/17840461]^73^. XAS data generated in this study have been deposited in the ESRF database under the Experiment session: A31-1-307 on beamline BM31 [10.15151/ESRF-DC-2420488713]^74^. Data are available from the corresponding authors upon request.

## Code availability

This study did not generate new code.
