# Supplementary material for: Interfacial W–O–Zr ensembles in tungstated zirconia catalysts enable efficient hydrogen-free recycling of polypropylene waste
Source: Nat Commun. 2026 May 22;17:6708. doi: 10.1038/s41467-026-73420-6 (PMC13385888; doi:10.1038/s41467-026-73420-6)
Supplement: Supplementary file 1 — Supplementary Information [file 41467_2026_73420_MOESM1_ESM.pdf]

Supplementary Information

Interfacial W–O–Zr ensembles in tungstated zirconia enable hydrogen-free recycling of polypropylene waste

Sibei Zou,[1,2‡] Yuzhen Ge,[1,2‡] Patrik O. Willi,[1,2] Javier Fernández-González,[1,2] Robert N. Grass,[1,2] Wendelin J. Stark,[1,2] Gonzalo Guillén-Gosálbez,[1,2] Antonio J. Martín,[1,2] and Javier Pérez-Ramírez[1,2*]

[1] Institute of Chemical and Bioengineering, Department of Chemistry and Applied Biosciences, ETH Zurich, Vladimir-Prelog-Weg 1, 8093 Zurich, Switzerland.

[2] NCCR Catalysis, 8093 Zurich, Switzerland.

[‡] Equal contribution.

[*] Corresponding author. E-mail: jpr@chem.ethz.ch.

Table of Contents

Supplementary Notes 2

Supplementary Tables 5

Supplementary Figures..32

Supplementary References 74

Supplementary Notes

Supplementary Note 1 | Raman spectroscopic analyses of W_aZr_b catalysts.

The Raman spectroscopy analysis (**Fig. 4c**) of the W_3Zr_{97} catalyst exhibited a symmetric W=O vibration (987 cm^{-1}) and a weak asymmetric vibration (824 cm^{-1}), characteristic of isolated tungstate $[WO_4]^{2-}$ without discernible bridging features.[1] This was consistent with a low density of W–O–Zr linkages detectable by EXAFS but below the Raman detection threshold. In the case of $W_{10}Zr_{90}$, ZrO_2 bands (W_0Zr_{100}, 150-650 cm^{-1} region) nearly vanished, while the W=O modes red-shifted to 1014 cm^{-1} and 911 cm^{-1}, consistent with aggregation into sub-nanoclusters.[2] A distinctive band at 811 cm^{-1} in $W_{10}Zr_{90}$ was attributed to W–O–Zr, consistent with the second shell W–Zr coordination observed from W L_3-edge EXAFS (**Fig. 4b**). For $W_{60}Zr_{40}$, crystalline WO_3 doublets (706/806 cm^{-1}) dominated, accompanied by residual W=O vibrations, in line with microscopy observations. Collectively, Raman studies confirmed W–O–Zr bridges stabilized in sub-nanoclusters as a distinctive feature of $W_{10}Zr_{90}$.

Supplementary Note 2 | *Operando* infrared spectroscopic analyses of W_aZr_b catalysts.

Operando diffuse reflectance infrared Fourier transform spectroscopy, DRIFTS was employed to track polymer vibrational modes and surface OH dynamics in the catalysts under reaction conditions.[3,4] For PP_{12k} mixed with $W_{10}Zr_{90}$, analysis of the C–H stretching region (2600-3200 cm^{-1}) revealed that at 60 °C the intensities of methyl ($–CH_3$) and methylene ($–CH_2–$) vibrations were comparable (**Fig. 6b**), in line with the expected PP signature.[5] With increasing temperature, the $–CH_2–$ backbone bands decreased substantially faster than the $–CH_3$ side-chain/terminal bands, evidencing a highly selective main-chain β-scission pathway. At the PP_{12k} melting point (ca. 160 °C), the $–CH_2–$ bands exhibited a pronounced blue shift, while the OH stretching band of $W_{10}Zr_{90}$ simultaneously red shifted with increasing temperature (**Fig. 6c**). The correlation between the $–CH_2–$ blue shift and OH red shift suggested strong interactions between the polymer backbone and surface OH groups on $W_{10}Zr_{90}$.[3] Comparison with other catalysts further underscored the unique character of OH groups in $W_{10}Zr_{90}$. $W_{100}Zr_0$ exhibited no OH stretching before or after reaction (**Supplementary Fig. 31)**, consistent with the absence of Brønsted acid sites observed in pyridine-FTIR results (**Supplementary Fig. 21**). $W_{60}Zr_{40}$ initially lacked OH stretching features but generated them during reaction (**Supplementary Fig. 32**). Although their population accumulated with increasing temperature, the absence of a red shift indicated they were not involved in PP depolymerization. In contrast, W_0Zr_{100} displayed OH stretching initially, which gradually decreased with increasing temperature but again without a red shift, consistent with the simple OH desorption as water and pointing to the inactivity of Brønsted acid sites in ZrO_2 (**Supplementary Fig. 35**). Thus, only the OH groups on $W_{10}Zr_{90}$ were directly involved in C–C bond β-scission.

Supplementary Note 3 | EXAFS fitting procedure.

The collected XAS spectra were energy-calibrated, background-corrected, and normalized using the Athena program within the Demeter software suite. The k^2-weighted EXAFS spectra were fitted in the optimal *k*- and *R*-windows using the Artemis program.

For the W L_3-edge analysis, $Zr(WO_4)_2$ and WO_3 were selected as physically reasonable local reference structures (**Supplementary Table 10**). For the first-shell W–O coordination, scattering paths derived from both $Zr(WO_4)_2$ and WO_3 were employed, depending on the geometry of the W species. $Zr(WO_4)_2$-derived paths were used to model second-shell W–Zr coordination, whereas WO_3-derived paths were applied for second-shell W–W coordination. The following parameters were used during the fitting process: S_0 = 0.9, k = 3.0−11.9 Å^{-1}, R = 1−4 Å. EXAFS spectra in *k*-space are shown in **Supplementary Fig. 16**, and fitting profiles in *R*-space with magnitude and imaginary parts are available in **Supplementary Fig. 17**.

For the Zr *K*-edge analysis, tetragonal ZrO_2 was used as the reference structure to fit the first-shell Zr–O and second shell Zr–Zr scattering paths (**Supplementary Table 11, 12**). An additional second shell Zr–W scattering path derived from $Zr(WO_4)_2$ was included to account for interfacial WO_x–ZrO_2 interactions. The following parameters were used during the fitting process: S_0 = 0.9, k = 3.0−14.2 Å^{-1}, R = 1−4 Å. EXAFS spectra for ex-situ samples in *k*-space can be found in **Supplementary Fig. 16**, and fitting profiles in *R*-space with magnitude and imaginary parts in **Supplementary Fig. 18**. EXAFS spectra for *Operando* sample in *k*-space are shown in **Supplementary Fig. 22**, and fitting profiles in *R*-space with magnitude and imaginary parts were in **Supplementary Fig. 23**.

Supplementary Tables

Supplementary Table 1 | Chemical composition of W_aZr_b catalysts measured by XRF.

Catalyst	Content / mol%	
	W	Zr
W_0Zr_{100}	0.0	100.0
W_1Zr_{99}	1.3	98.7
W_3Zr_{97}	3.7	96.3
W_5Zr_{95}	6.5	93.5
$W_{10}Zr_{90}$	11.9	88.1
$W_{20}Zr_{80}$	21.5	78.5
$W_{30}Zr_{70}$	30.7	69.3
$W_{40}Zr_{60}$	41.3	58.7
$W_{50}Zr_{50}$	50.4	49.6
$W_{60}Zr_{40}$	59.5	40.5
$W_{70}Zr_{30}$	69.7	30.3
$W_{80}Zr_{20}$	79.5	20.5
$W_{90}Zr_{10}$	88.6	11.4
$W_{100}Zr_0$	100.0	0.0

Supplementary Table 2 | Product distributions from polypropylene depolymerization over W_aZr_b catalysts. Reaction conditions: 0.25 g catalyst, 0.5 g PP_{12k}, 4 bar N_2, 240 °C, 4 h, 750 rpm.

Catalyst	Conversion / %	Yield / %					Gasoline selectivity / %
		C_1-C_3	C_4-C_{12}	C_{13}-C_{16}	C_{17}-C_{35}	C_{36+}	
W_0Zr_{100}	22	0	18	3	2	77	78
W_1Zr_{99}	33	1	27	3	2	67	83
W_3Zr_{97}	67	2	56	5	4	33	84
W_5Zr_{95}	78	4	71	2	1	21	91
$W_{10}Zr_{90}$	92	3	82	6	1	8	89.
$W_{20}Zr_{80}$	78	5	60	6	8	22	77
$W_{30}Zr_{70}$	73	3	62	7	1	27	85
$W_{40}Zr_{60}$	73	3	66	3	2	27	89
$W_{50}Zr_{50}$	77	3	65	8	2	23	83
$W_{60}Zr_{40}$	69	3	59	6	1	31	85
$W_{70}Zr_{30}$	71	3	57	8	2	29	81
$W_{80}Zr_{20}$	63	3	51	8	2	37	82
$W_{90}Zr_{10}$	72	6	59	5	3	28	81
$W_{100}Zr_0$	54	2	45	6	2	46	82

Supplementary Table 3 | Product distributions from polypropylene depolymerization over W_aZr_b catalysts at different reacting conditions.

Time / h	*T* / ºC	Catalyst-to-PP mass ratio	*P* / bar	Conversion / %	Yield / %					Gasoline selectivity / %
					C_1-C_3	C_4-C_{12}	C_{13}-C_{16}	C_{17}-C_{35}	C_{36+}	
0.2	240	1:2[a]	4	74	2	65	6	2	26	88
1	240	1:2	4	77	3	65	7	3	23	84
4	160	1:2	4	57	2	39	7	9	43	68
4	180	1:2	4	52	1	42	4	4	48	82
4	200	1:2	4	100	2	75	11	13	0	75
4	240	1:2	4	92	3	82	6	1	8	89
4	240	2:1[b]	4	100	7	90	3	1	0	90
12	180	1:2	4	70	2	51	12	6	30	74
12	240	1:2	4	91	5	78	5	3	9	86
24	240	1:2	4	98	5	83	6	4	2	85
4	240	1:2	10	91	3	79	5	4	9	88
4	240	1:2	20	78	2	67	6	4	22	85
4	240	1:2	30	71	2	60	6	4	29	83

[a] 0.25 g catalyst, 0.5 g PP_{12k}, 750 rpm. [b] 0.5 g catalyst, 0.25 g PP_{12k}, 750 rpm.

Supplementary Table 4 | ^{1}H NMR chemical shift assignment.

Chemical shift / ppm	Assignment
0.7-1.0	Alkanes, R–CH_3
1.2-1.5	Alkanes, R–CH_2–R
1.4-1.8	Alkanes, R–CH–R
4.5-6.5	Olefinic, C=CH
2.2-2.7	α to aromatic ring (Ar), Ar–CH_2 / Ar–CH_2–
6.5-8.5	Aromatic, Ar–H

Supplementary Table 5 | Distribution of C_1-C_5 products from polypropylene depolymerization over $W_{10}Zr_{90}$ at different reaction times. Reaction conditions: 0.25 g catalyst, 0.5 g PP_{12k}, 4 bar N_2, 240 °C, 750 rpm.

Time / h	Selectivity / %			
	n-Alkanes	*i*-Alkanes	Alkenes	Cycloalkanes
0.2	2	67	26	5
1	2	60	31	8
4	2	61	33	4
12	4	59	29	8
24	5	57	33	6

Supplementary Table 6 | Product distributions from polypropylene depolymerization over $W_{10}Zr_{90}$ in consecutive runs. Reaction conditions: 0.25 g catalyst, 0.5 g PP_{12k}, 4 bar N_2, 240 °C, 4 h, 750 rpm.

Run	Conversion / %	Yield / %					Gasoline selectivity / %
		C_1-C_3	C_4-C_{12}	C_{13}-C_{16}	C_{17}-C_{35}	C_{36+}	
1	92	3	82	6	1	8	89
2	95	4	85	5	1	5	89
3	94	4	82	6	1	6	88
4	100	5	87	7	1	0	88
5	92	4	75	9	4	8	81

Supplementary Table 7 | Product distributions from polypropylene depolymerization over $W_{10}Zr_{90}$ for different amounts of PP_{12}. Reaction conditions: catalyst:PP_{12k} = 1:2 (w/w), 4 bar, 240 °C, 4 h, 750 rpm.

PP_{12k} / g	Conversion / %	Yield / %					Gasoline selectivity / %
		C_1-C_3	C_4-C_{12}	C_{13}-C_{16}	C_{17}-C_{35}	C_{36+}	
0.5	92	3	82	6	1	8	89
3.0	72	2	64	4	2	28	88

Supplementary Table 8 | Product distributions from polypropylene depolymerization over $W_{10}Zr_{90}$ for different virgin polymers and consumer goods. Reaction conditions: 0.25 g catalyst, 0.5 g plastics, 4 bar N_2, 750 rpm.

Feedstock	Conversion / %	Yield / %					Gasoline selectivity / %
		C_1-C_3	C_4-C_{12}	C_{13}-C_{16}	C_{17}-C_{35}	C_{36+}	
PP_{12k}[a]	92	3	82	6	1	8	89
PP_{250K}[b]	93	8	81	3	1	7	87
Centrifuge tube[c]	80	3	70	5	2	20	88
Yogurt cup[c]	69	3	59	6	1	31	85
Shampoo lid[c]	82	3	72	6	1	18	88

[a] 240 °C, 4 h. [b] 260 °C, 12 h. [c] 240 °C, 12 h.

Supplementary Table 9 | Product distributions from polypropylene depolymerization over $W_{10}Zr_{90}$ and the WZr-KIT6+HZSM-5 catalyst.[3] Reaction conditions: 0.25 g catalyst, 0.5 g plastic, 4 bar N_2, 240 °C, 4 h, 750 rpm.

Catalyst	Conversion / %	Yield / %					Gasoline selectivity / %
		C_1-C_3	C_4-C_{12}	C_{13}-C_{16}	C_{17}-C_{35}	C_{36+}	
$W_{10}Zr_{90}$	92	3	82	6	1	8	89
WZr-KIT6+HZSM-5	74	2	65	4	3	26	85

Supplementary Table 10 | W L_3-edge EXAFS fitting results for representative W_aZr_b catalysts.[a]

Catalyst	Path	*R* / Å	CN[b] / −	ΔE[0] / eV	σ^2 / Å[2]	*R* factor	Model[b]
W_3Zr_{97}	W–O_1	1.65 ± 0.02	1.7 ± 0.4	−4.8	0.001 ± 0.001	0.03	$Zr(WO_4)_2$
	W–O_2	1.81 ± 0.03	2.1 ± 0.1		0.001 ± 0.001		$Zr(WO_4)_2$
	W–O_3	2.48 ± 0.05	1.2 ± 0.1		0.001 ± 0.001		$Zr(WO_4)_2$
	W–Zr	3.83 ± 0.06	2.9 ± 0.1		0.006 ± 0.005		$Zr(WO_4)_2$
	W–W	3.48 ± 0.08	2.1 ± 0.1		0.006 ± 0.001		WO_3
$W_{10}Zr_{90}$	W–O_1	1.74 ± 0.02	3.3 ± 0.1	-2.9	0.008 ± 0.003	0.03	$Zr(WO_4)_2$
	W–O_2	1.89 ± 0.09	0.8 ± 0.8		0.008 ± 0.001		$Zr(WO_4)_2$
	W–O_3	2.51 ± 0.01	0.9 ± 0.1		0.008 ± 0.001		$Zr(WO_4)_2$
	W–Zr	3.89 ± 0.03	2.6 ± 0.1		0.001 ± 0.003		$Zr(WO_4)_2$
	W–W	3.51 ± 0.04	2.4 ± 0.1		0.003 ± 0.003		WO_3
$W_{60}Zr_{40}$	W–O_1	1.78 ± 0.01	3.7 ± 0.1	2.7	0.006 ± 0.001	0.01	WO_3
	W–O_2	2.06 ± 0.01	2.3 ± 0.1		0.006 ± 0.001		WO_3
	W–Zr	3.93 ± 0.09	1.8 ± 0.1		0.001 ± 0.004		$Zr(WO_4)_2$
	W–W	3.48 ± 0.03	4.2 ± 0.5		0.003 ± 0.003		WO_3
$W_{100}Zr_0$	W–O_1	1.75 ± 0.01	3.2 ± 0.3	4.9	0.004 ± 0.001	0.01	WO_3
	W–O_2	2.16 ± 0.04	2.8 ± 0.1		0.004 ± 0.001		WO_3
	W–W	3.49 ± 0.04	3.9 ± 0.1		0.001 ± 0.003		WO_3

[a] S_0 was fixed as 0.9 during the fitting process in three samples. k = 3.0−11.9 Å^{-1}, R = 1−4 Å.

[b] $Zr(WO_4)_2$ and WO_3 were used as fitting models for the W L_3-edge EXAFS analysis, with $Zr(WO_4)_2$ representing the interfacial coordination environment of WO_x/ZrO_2 and WO_3 representing the coordination environments of three-dimensional WO_x domains and WO_3.

Supplementary Table 11 | Zr *k*-edge EXAFS fitting results for representative W_aZr_b catalysts.[a]

Catalyst	Path[b]	R / Å	CN[c] / −	ΔE^0 / eV	σ^2 / Å^2	R factor	$CN_{Zr–W}$ ratio[d] / %
W_0Zr_{100}	Zr–O$_1$	2.11 ± 0.01	5.2 ± 0.3	−3.0	0.005 ± 0.001	0.01	0
	Zr–O$_2$	2.34 ± 0.01	2.8 ± 0.0		0.003 ± 0.002		
	Zr–Zr$_1$	3.61 ± 0.02	7.2 ± 0.1		0.003 ± 0.001		
	Zr–Zr$_2$	3.71 ± 0.01	4.8 ± 0.9		0.003 ± 0.001		
W_3Zr_{97}	Zr–O$_1$	2.10 ± 0.01	5.0 ± 0.4	−3.0	0.005 ± 0.001	0.03	5.0
	Zr–O$_2$	2.34 ± 0.02	2.9 ± 0.2		0.005 ± 0.001		
	Zr–W	3.50 ± 0.01	0.6 ± 0.1		0.007 ± 0.002		
	Zr–Zr$_1$	3.65 ± 0.01	7.4 ± 0.1		0.001 ± 0.001		
	Zr–Zr$_2$	3.68 ± 0.02	3.7 ± 0.1		0.005 ± 0.002		
W_5Zr_{95}	Zr–O$_1$	2.11 ± 0.01	5.4 ± 0.5	−3.0	0.005 ± 0.002	0.03	10.0
	Zr–O$_2$	2.30 ± 0.03	2.6 ± 0.1		0.005 ± 0.001		
	Zr–W	3.47 ± 0.03	1.2 ± 0.3		0.002 ± 0.002		
	Zr–Zr$_1$	3.63 ± 0.01	6.8 ± 0.1		0.002 ± 0.002		
	Zr–Zr$_2$	3.64 ± 0.03	4.0 ± 0.1		0.002 ± 0.001		
$W_{10}Zr_{90}$	Zr–O$_1$	2.10 ± 0.01	5.1 ± 0.2	−4.0	0.005 ± 0.003	0.01	15.8
	Zr–O$_2$	2.34 ± 0.03	2.9 ± 0.1		0.006 ± 0.006		
	Zr–W	3.59 ± 0.01	1.9 ± 0.0		0.005 ± 0.003		
	Zr–Zr$_1$	3.62 ± 0.09	6.1 ± 0.1		0.002 ± 0.001		
	Zr–Zr$_2$	3.71 ± 0.08	4.0 ± 0.1		0.002 ± 0.001		

(continued from the previous page)

$W_{30}Zr_{70}$	Zr–O_1	2.11 ± 0.01	5.2 ± 0.1	0.0	0.005 ± 0.002	0.03	9.2
	Zr–O_2	2.30 ± 0.02	2.8 ± 0.6		0.005 ± 0.000		
	Zr–W	3.45 ± 0.03	1.1 ± 0.1		0.002 ± 0.002		
	Zr–Zr_1	3.62 ± 0.01	6.9 ± 0.1		0.003 ± 0.001		
	Zr–Zr_2	3.64 ± 0.02	4.0 ± 0.1		0.002 ± 0.001		
$W_{40}Zr_{60}$	Zr–O_1	2.12 ± 0.01	5.4 ± 0.4	−3.0	0.005 ± 0.001	0.03	4.2
	Zr–O_2	2.31 ± 0.02	2.6 ± 0.1		0.002± 0.005		
	Zr–W	3.45 ± 0.03	0.5 ± 0.1		0.001 ± 0.005		
	Zr–Zr_1	3.61± 0.02	7.5 ± 0.1		0.005 ± 0.001		
	Zr–Zr_2	3.63± 0.04	4.0 ± 0.1		0.001 ± 0.001		
$W_{60}Zr_{40}$	Zr–O_1	2.11 ± 0.01	5.1 ± 0.3	−3.0	0.005 ± 0.001	0.03	2.5
	Zr–O_2	2.35 ± 0.02	2.9 ± 0.2		0.002 ± 0.002		
	Zr–W	3.50 ± 0.08	0.3 ± 0.0		0.005 ± 0.005		
	Zr–Zr_1	3.58 ± 0.02	7.7 ± 0.1		0.003 ± 0.003		
	Zr–Zr_2	3.64 ± 0.08	3.9 ± 0.0		0.001 ± 0.002		
$W_{80}Zr_{20}$	Zr–O_1	2.08 ± 0.01	2.6 ± 0.3	−3.0	0.001 ± 0.001	0.05	1.9
	Zr–O_2	2.23 ± 0.01	2.4 ± 0.1		0.001 ± 0.001		
	Zr–W	3.47 ± 0.06	0.2 ± 0.1		0.001 ± 0.005		
	Zr–Zr_1	3.64 ± 0.02	6.2 ± 0.1		0.004 ± 0.002		
	Zr–Zr_2	3.69 ± 0.05	4.0 ± 0.1		0.001 ± 0.001		

[a] S_0 was fixed as 0.9 during the fitting process in three samples. k = 3.0−14.2 $Å^{-1}$, R = 1−3 Å. [b] Tetragonal ZrO_2 was used as the fitting model.
[c] CN: coordination number. [d] $CN_{Zr\text{-}W}$ ratio = $CN_{Zr–W}/\sum CN$.

Supplementary Table 12 | *Operando* Zr *k*-edge EXAFS fitting results for $W_{10}Zr_{90}$. Reaction conditions: catalyst:PP_{12k} = 2:1 (w/w), 4 bar He.[a]

T / °C	Path[b]	R / Å	CN[c] / −	ΔE^0 / eV	σ^2 / Å^2	R factor	$CN_{Zr–W}$ ratio[d] / %
RT	Zr–O_1	2.11 ± 0.01	5.3 ± 0.4	-4.5	0.004 ± 0.001	0.03	10
	Zr–O_2	2.31 ± 0.02	2.7 ± 0.0		0.004 ± 0.001		
	Zr–W	3.57 ± 0.00	1.9 ± 0.1		0.002 ± 0.001		
	Zr–Zr_1	3.60 ± 0.01	6.0 ± 0.1		0.002 ± 0.001		
	Zr–Zr_2	3.71 ± 0.01	4.1 ± 0.1		0.002 ± 0.002		
160	Zr–O_1	2.11 ± 0.01	5.3 ± 0.1	-4.0	0.004 ± 0.001	0.03	10
	Zr–O_2	2.30 ± 0.02	2.7 ± 0.5		0.004 ± 0.001		
	Zr–W	3.57 ± 0.01	1.9 ± 0.1		0.003 ± 0.001		
	Zr–Zr_1	3.61 ± 0.01	6.3 ± 0.1		0.003 ± 0.001		
	Zr–Zr_2	3.71 ± 0.02	3.8 ± 0.1		0.003 ± 0.002		
180	Zr–O_1	2.11 ± 0.01	5.3 ± 0.1	-4.0	0.004 ± 0.001	0.03	19
	Zr–O_2	2.30 ± 0.02	2.7 ± 0.5		0.004 ± 0.001		
	Zr–W	3.56 ± 0.01	2.5 ± 0.0		0.003 ± 0.001		
	Zr–Zr_1	3.61 ± 0.01	6.1 ± 0.1		0.003 ± 0.001		
	Zr–Zr_2	3.72 ± 0.03	3.4 ± 0.0		0.003 ± 0.001		
200	Zr–O_1	2.11 ± 0.01	5.3 ± 0.1	-4.0	0.004 ± 0.001	0.04	25
	Zr–O_2	2.30 ± 0.03	2.7 ± 0.5		0.004 ± 0.001		
	Zr–W	3.56 ± 0.01	2.9 ± 0.1		0.002 ± 0.001		
	Zr–Zr_1	3.60 ± 0.01	5.3 ± 0.1		0.002 ± 0.001		
	Zr–Zr_2	3.71 ± 0.01	3.9 ± 0.1		0.002 ± 0.001		

(continued from the previous page)

220	$Zr–O_1$	2.10 ± 0.02	5.1 ± 0.1	-3.5	0.004 ± 0.002	0.04	25
	$Zr–O_2$	2.28 ± 0.03	2.9 ± 0.7		0.004 ± 0.001		
	Zr–W	3.52 ± 0.01	2.9 ± 0.1		0.001 ± 0.001		
	$Zr–Zr_1$	3.59 ± 0.01	4.2 ± 0.1		0.001 ± 0.002		
	$Zr–Zr_2$	3.71 ± 0.01	4.9 ± 0.1		0.002 ± 0.002		
240	$Zr–O_1$	2.10 ± 0.01	5.5 ± 0.5	-4.5	0.005 ± 0.001	0.04	25
	$Zr–O_2$	2.28 ± 0.03	2.5 ± 0.3		0.005 ± 0.001		
	Zr–W	3.57 ± 0.01	3.0 ± 0.1		0.003 ± 0.001		
	$Zr–Zr_1$	3.60 ± 0.01	5.5 ± 0.1		0.003 ± 0.001		
	$Zr–Zr_2$	3.71 ± 0.01	3.5 ± 0.1		0.003 ± 0.001		

[a] S_0 was fixed as 0.9 during the fitting process in three samples. k = 3.0−14.2 $Å^{-1}$, R = 1−3 Å. [b] Tetragonal ZrO_2 was used as the fitting model.
[c] CN: coordination number. [d] CN_{Zr-W} ratio = $CN_{Zr–W}/\sum CN$.

Supplementary Table 13 | Product distributions from polypropylene depolymerization over $W_{10}Zr_{90}$ under different atmospheres. Reaction conditions: 0.25 g catalyst, 0.5 g PP_{12k}, 4 bar, 240 °C, 4 h, 750 rpm.

Atmosphere	Conversion / %	Yield / %					Gasoline selectivity / %
		C_1-C_3	C_4-C_{12}	C_{13}-C_{16}	C_{17}-C_{35}	C_{36+}	
N_2	92	3	82	6	1	8	89
H_2	94	4	85	5	1	6	90

Supplementary Table 14 | Product distributions from depolymerization of different polyolefins over $W_{10}Zr_{90}$. Reaction conditions: 0.25 g catalyst, 0.5 g plastic, 4 bar N_2, 240 °C, 4 h, 750 rpm.

Plastic	Conversion / %	Yield / %					Gasoline selectivity / %
		C_1-C_3	C_4-C_{12}	C_{13}-C_{16}	C_{17}-C_{35}	C_{36+}	
PP_{12k}	92	3	82	6	1	8	89
$HDPE_{100k}$	24	0	20	3	2	76	81

Supplementary Table 15 | Variables used for the annualized capital cost, ACC calculation. The Chemical Engineering Plant Cost Index, CEPCI is used to account for inflation over the years and adequately adjust the capital expenditures calculated with cost correlations from Towler & Sinnott[6] that provide costs in USD 2010.

Variable	Value	Unit
Operation hours per year	8000	h y^{-1}
Plant lifetime	25	y
Interest rate	10	%
CEPCI 2010	533	USD
CEPCI 2024	799	USD

Supplementary Table 16 | Parameters used to calculate the purchase equipment cost, C per type of equipment ($C = a + bS^n$). Values for base a and b in USD 2010. a: fixed cost term; b: variable cost term; n: scaling factor; S: characteristic size parameter.

Equipment type	a	b	n	S	Reference
Plate and frame filter	128000	89000	0.50	Volume / m^3	Towler & Sinnot[6]
Decanter centrifuge	0	48000	0.70	Flow / $m^3\ h^{-1}$	Olmstead[7]
Single-stage centrifugal pump	8000	240	0.90	Flow / $L\ s^{-1}$	Towler & Sinnot[6]
Pressure vessel	11600	34	0.85	Mass / kg	Towler & Sinnot[6]
Sieve trays (1 tray)	130	440	1.80	Length /m	Towler & Sinnot[6]
U-tube shell and tube heat exchanger	28000	54	1.20	Area / m^2	Towler & Sinnot[6]
U-tube Kettle reboiler	29000	400	0.90	Area / m^2	Towler & Sinnot[6]

Supplementary Table 17 | Cost parameters considered for the operation expenditure calculations in the technoeconomic assessment. They include raw materials, electricity, heating, and cooling utilities used in the chemical recycling process. All costs are presented in USD per unit, with the unit matching the units of the life cycle inventories (**Supplementary Tables 19, 20**).

Item	Cost low / USD unit^{-1}	Cost avg. / USD unit^{-1}	Cost high / USD unit^{-1}	Reference
Waste PP	0.227[a]	0.297	0.527[a]	Salah[8]
DCM solvent	-	0.546	-	ChemAnalyst[9]
Residue disposal	0.005	0.059	0.107	CEWEP[10]
Catalyst	-	14.98	-	Metal prices[b]
Cooling water at 20-35 °C	-	$3.2 \cdot 10^{-4}$	-	Ioannou[11]
Cryogenic cooling at -25 °C	-	0.016	-	Ioannou[11]
Heating utility, natural gas	$2.31 \cdot 10^{-3}$	0.012	0.042	World Bank[12,c]
Electricity	0.011	0.19	0.465	GlobalPetrolPrices[13]

[a] Low and high values for waste polypropylene acquisition estimated from Eurostat.[14]

[b] The catalyst price was calculated based on the market prices for ZrO_2 (6.2 USD kg^{-1})[15] and WO_3 (57 USD kg^{-1}).[16]

[c] The heating utility cost was derived from the global average cost of natural gas in 2024.[17]

Supplementary Table 18 | Average, low, and high market prices of end products in 2024 used in the technoeconomic assessment.

Material / Energy flow	Price low / USD kg^{-1}	Price avg. / USD kg^{-1}	Price high / USD kg^{-1}	Reference
Light gases, C_1-C_3	0.12	0.32	0.70	Trading Economics[18]
Gasoline, C_4-C_{12}	1.04	1.16	1.46	Business Analytiq[19]
Diesel, C_{13}-C_{20}	1.08	1.21	1.62	Business Analytiq[20]
Motor oil, C_{21}-C_{35}	1.05	1.50	2.08	Business Analytiq[21]

Supplementary Table 19 | Life cycle inventory, LCI of the chemical recycling process shown in **Supplementary Fig. 36**. Mass and energy flows were obtained from the process flowsheet and normalized per kilogram of product generated.

Material / Energy flow	PP_{12K}	PP_{250K}	Centrifuge tube	Yogurt cup	Shampoo lid	Unit	Reference LCI
Light gases, C_1-C_3	0.032	0.098	0.038	0.044	0.037	kg	Ecoinvent 3.10[22]
Gasoline, C_4-C_{12}	0.818	0.865	0.875	0.855	0.878	kg	Ecoinvent 3.10[22]
Diesel, C_{13}-C_{20}	0.118	0.032	0.062	0.072	0.061	kg	Ecoinvent 3.10[22]
Motor oil, C_{21}-C_{35}	0.032	0.005	0.025	0.029	0.025	kg	Ecoinvent 3.10[22]
Waste PP	1.063	1.081	1.248	1.449	1.219	kg	Salah[8]
Cooling water at 20-35 °C	1.522	1.606	1.770	1.908	1.695	MJ	Ioannou[11]
Cryogenic cooling at -25 °C	0.015	0.047	0.000	0.021	0.017	MJ	Luyben[23]
Heating utility, natural gas	1.971	2.164	1.742	1.206	2.252	MJ	Ecoinvent 3.10[22]
Electricity	$6.2 \cdot 10^{-4}$	$8.6 \cdot 10^{-3}$	$6.5 \cdot 10^{-4}$	$6.9 \cdot 10^{-4}$	$6.4 \cdot 10^{-4}$	kWh	Ecoinvent 3.10[22]
Catalyst	$2.1 \cdot 10^{-3}$	$2.1 \cdot 10^{-3}$	$2.5 \cdot 10^{-3}$	$2.9 \cdot 10^{-3}$	$2.4 \cdot 10^{-3}$	kg	**Supplementary Table 18**
Residue disposal	0.063	0.081	0.248	0.449	0.220	kg	Ecoinvent 3.10[22]
DCM solvent	$8.1 \cdot 10^{-4}$	$8.3 \cdot 10^{-4}$	$9.5 \cdot 10^{-4}$	$1.1 \cdot 10^{-3}$	$9.3 \cdot 10^{-4}$	kg	Ecoinvent 3.10[22]

Supplementary Table 20 | Life cycle inventory of the components considered to model one kilogram of the $W_{10}Zr_{90}$ catalyst.

Material / Energy flow	Amount	Unit	Reference LCI
Zirconium oxide	0.827	kg	Ecoinvent 3.10[22]
Tungsten oxide	0.173	kg	Ecoinvent 3.10[22]

Supplementary Table 21| Reference Ecoinvent 3.10 background data used as input flows to life cycle inventories described in **Supplementary Tables 19, 20**.

Material / Energy flow	Reference product	Activity name	Location
Light gases, C_1-C_3	propane	natural gas liquids fractionation	GLO
Gasoline, C_4-C_{12}	light fuel oil	light fuel oil production, petroleum refinery operation	RoW
Diesel, C_{13}-C_{20}	diesel	diesel production, petroleum refinery operation	RoW
Motor oil, C_{21}-C_{35}	heavy fuel oil	heavy fuel oil production, petroleum refinery operation	RoW
PP primary production	polypropylene, granulate	polypropylene production, granulate	GLO
Waste PP after sorting	waste polypropylene, for recycling, sorted	market for waste polypropylene, recovered, sorted	RoW
PP incineration	waste polypropylene	treatment of waste polypropylene, municipal incineration	RoW
PP landfilling	waste polypropylene	treatment of waste polypropylene, sanitary landfill	RoW
Heating utility, natural gas	heat, district or industrial, natural gas	market group for heat, district or industrial, natural gas	GLO
Electricity	electricity, high voltage	market group for electricity, high voltage	GLO
DCM solvent	dichloromethane	market for dichloromethane	RoW
Residue disposal	waste polypropylene	treatment of waste polypropylene, sanitary landfill	RoW
Zirconium oxide	zirconium oxide	market for zirconium oxide	GLO
Tungsten oxide	tungsten concentrate	market for tungsten concentrate	GLO

Supplementary Table 22 | Summary of studies used for the comparison of the chemical recycling routes. Works using real waste plastic feedstocks providing detailed product distribution per carbon chain length ranges were considered.

Number	Route	Item	Conditions	Catalyst	Yield / %						Reference
					C_1-C_5	$C_{2=}$-$C_{5=}$	C_6-C_{12}	C_{13}-C_{20}	C_{21}-C_{35}	C_{36+}	
1	H_2-free	PP lid	4 bar<br>240 °C	Zr_2W_3	3	0	70	5	2	6	This work
2	H_2-free	PP tube	4 bar<br>240 °C	Zr_2W_3	3	0	59	5	2	6	This work
3	H_2-free	PP cup	4 bar<br>240 °C	Zr_2W_3	3	0	72	5	2	6	This work
4	Hydrotreatment	PE cap	20 bar<br>250 °C	RuNi	24	0	6	13	23	34	N.-Langa.[24]
5	Hydrotreatment	PE strip	60 bar<br>220 °C	Ru-C	9	0	41	31	19	0	Jia[25]
6	Hydrotreatment	PE strip	30 bar<br>220 °C	Ru-C	26	0	60	14	0	0	Jia[25]
7	Hydrotreatment	PE wrap	25 bar<br>250 °C	MoS_x-Hbeta	56	0	31	7	1	5	Qiu[26]
8	Hydrotreatment	PE bottle	30 bar<br>250 °C	PtWZr	16	0	64	1	1	18	Liu[27]
9	Hydrotreatment	PE bottle	30 bar<br>300 °C	NiSi	22	0	20	31	1	26	Vance[28]
10	Hydrotreatment	PE jug	30 bar<br>275 °C	CoSi	57	0	5	21	9	9	Borkar[29]

(continued from the previous page)

11	Hydrotreatment	PE bottle	50 bar<br>250 °C	RuWZr	20	0	23	27	21	10	Wang[30]
12	Hydrotreatment	PP nonwoven	25 bar<br>250 °C	MoS_x-Hbeta	30	0	18	5	1	45	Qiu[26]
13	Hydrotreatment	PP tube	50 bar<br>225 °C	Ru-C	15	0	20	20	1	44	Rorrer[31]

Supplementary Table 23 | Literature performance data for the assessment of technologies. Life cycle inventory for the catalysts is modelled based on the reported mass content of each element. Works using real waste plastic feedstocks providing detailed product distribution per carbon chain length ranges were considered. A technosphere exchange from the Ecoinvent database is associated with each material flow to build the process inventory for each catalyst.

Number	Route	Catalyst	Catalyst-to-plastic ratio / -	Reaction time / h	Composition / wt%	Activity name	Reference
1-3	H_2-free	Zr_2W_3	0.5	4	83% Zr	market for zirconium oxide	This work
					17% W	market for tungsten concentrate	
4	Hydrotreatment	RuNi	0.1	6	2.5% Ru	market for platinum group metal concentrate[a]	N.-Langa[24]
					5.0% Ni	market for nickel, class 1	
					92.5% Ti	market for titanium	
5, 6, 13	Hydrotreatment	Ru-C	0.5	1	5% Ru	market for platinum group metal concentrate[a]	Jia[25]
					95% C	market for graphite	
7, 12	Hydrotreatment	MoS_x-Hbeta	0.1	6	80% zeolite	market for zeolite, powder	Qiu[26]
					10% S	market for sulfur	
					10% Mo	market for molybdenum trioxide	
8	Hydrotreatment	PtWZr	0.1	2	50% zeolite	market for zeolite, powder	Liu[27]
					0.25% Pt	market for platinum group metal concentrate	
					42.25% Zr	market for zirconium oxide	
					7.50% W	market for tungsten concentrate	
9	Hydrotreatment	NiSi	0.1	12	15% Ni	market for nickel, class 1	Vance[28]
					85% Si	market for silicon, metallurgical grade	

(continued from the previous page)

10	Hydrotreatment	CoSi	0.1	8	5% Co	market for cobalt oxide	Borkar[29]
					95% Si	market for silicon, metallurgical grade	
11	Hydrotreatment	RuWZr	0.025	2	5% Ru	market for platinum group metal concentrate[a]	Wang[30]
					15% W	market for tungsten concentrate	
					80% Zr	market for zirconium oxide	
12	Hydrotreatment	Ru-C	0.07	24	5% Ru	market for platinum group metal concentrate[a]	Rorrer[31]
					95% C	market for graphite	

[a] activity adapted for Ru using mass allocation; [b] assumption

Supplementary Figures

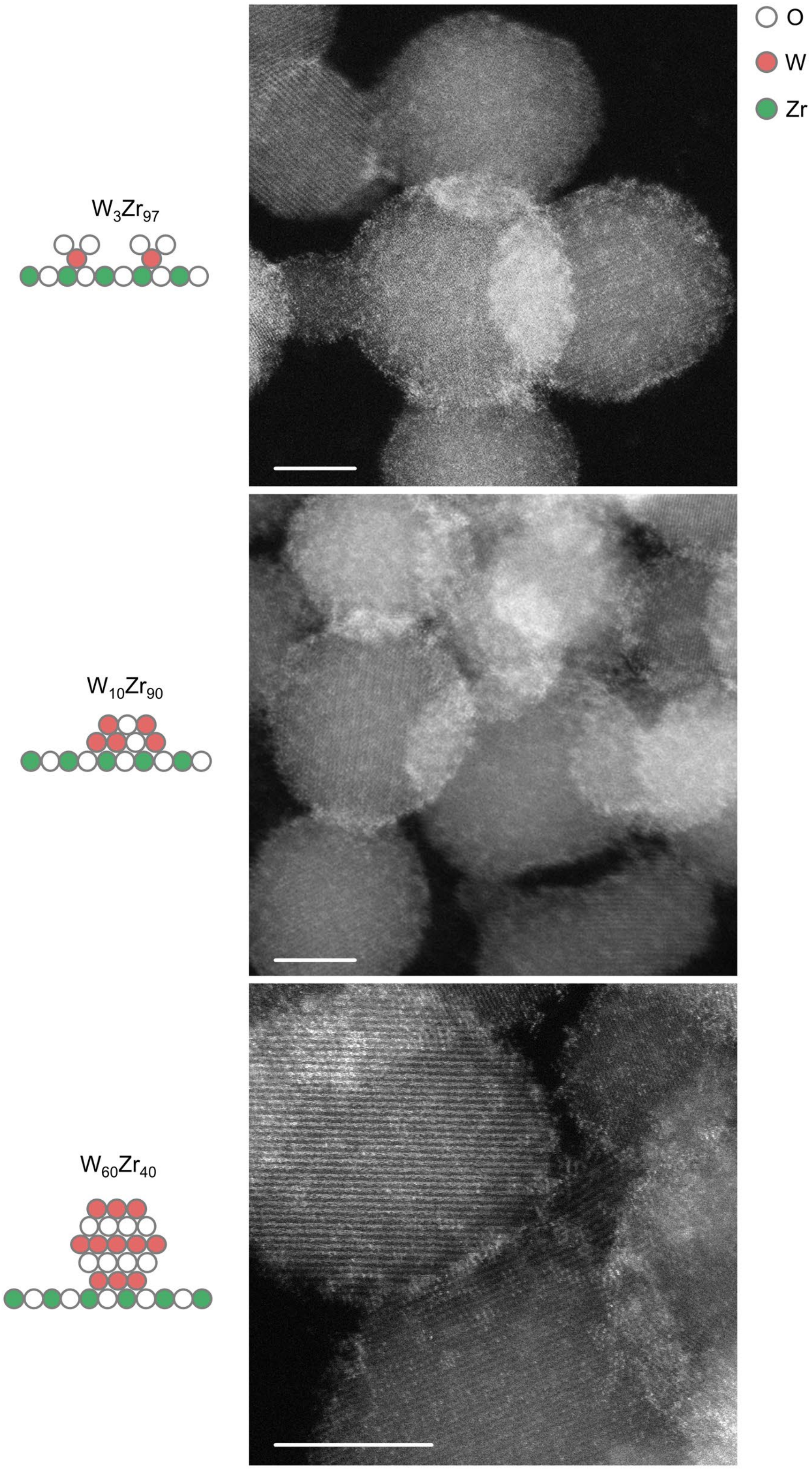

Supplementary Fig. 1 | Structural analysis of W_aZr_b catalysts. Representative general STEM-HAADF images of as-prepared W_3Zr_{97}, $W_{10}Zr_{90}$, and $W_{20}Zr_{80}$ catalysts showing the uniform size distribution of spherical zirconia particles. Scale bars represent 5 nm.

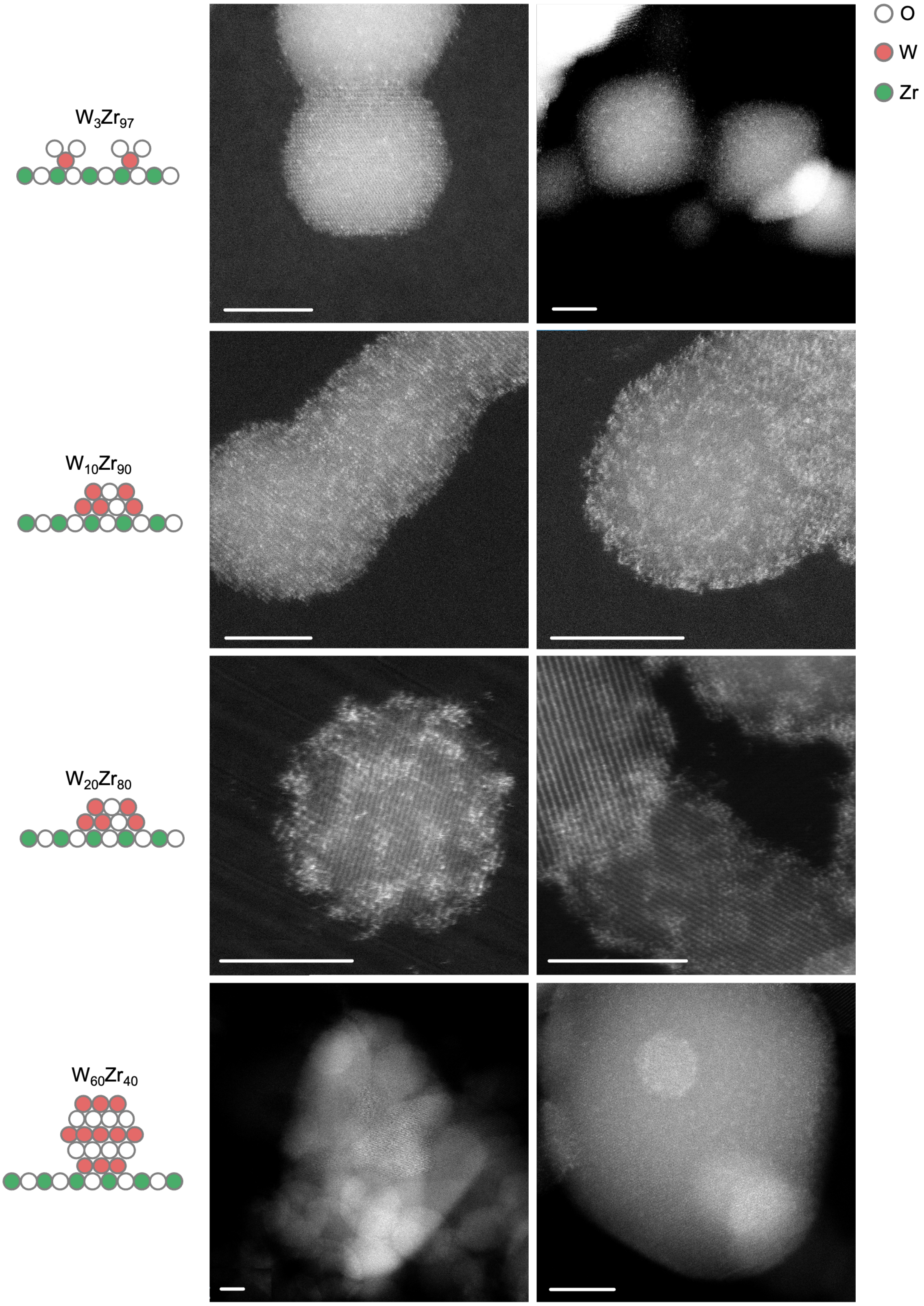

Supplementary Fig. 2 | Structural analysis of W_aZr_b catalysts. Additional STEM-HAADF images of as-prepared W_3Zr_{97}, $W_{10}Zr_{90}$, $W_{20}Zr_{80}$, and $W_{60}Zr_{40}$ catalysts showing distinct predominant tungsten species architectures. Scale bars represent 5 nm.

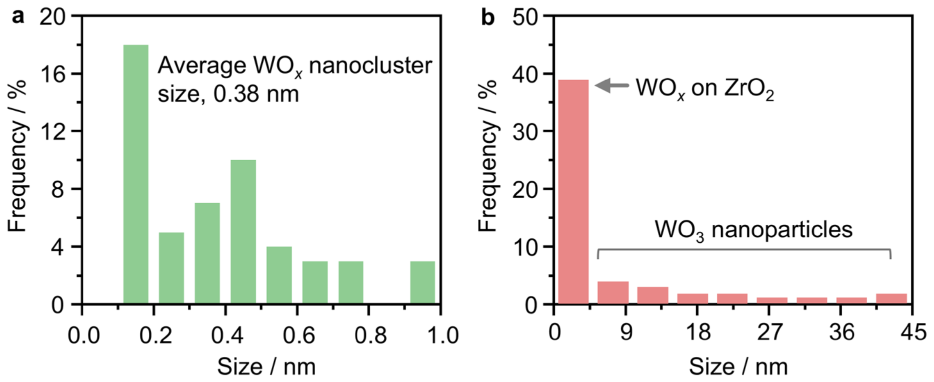

Supplementary Fig. 3 | Particle size analysis for W_aZr_b catalysts. Size distribution of tungsten species on bulk ZrO_2 for (**a**) $W_{10}Zr_{90}$ and (**b**) $W_{60}Zr_{40}$, measured from 50 particles in corresponding STEM-HAADF images.

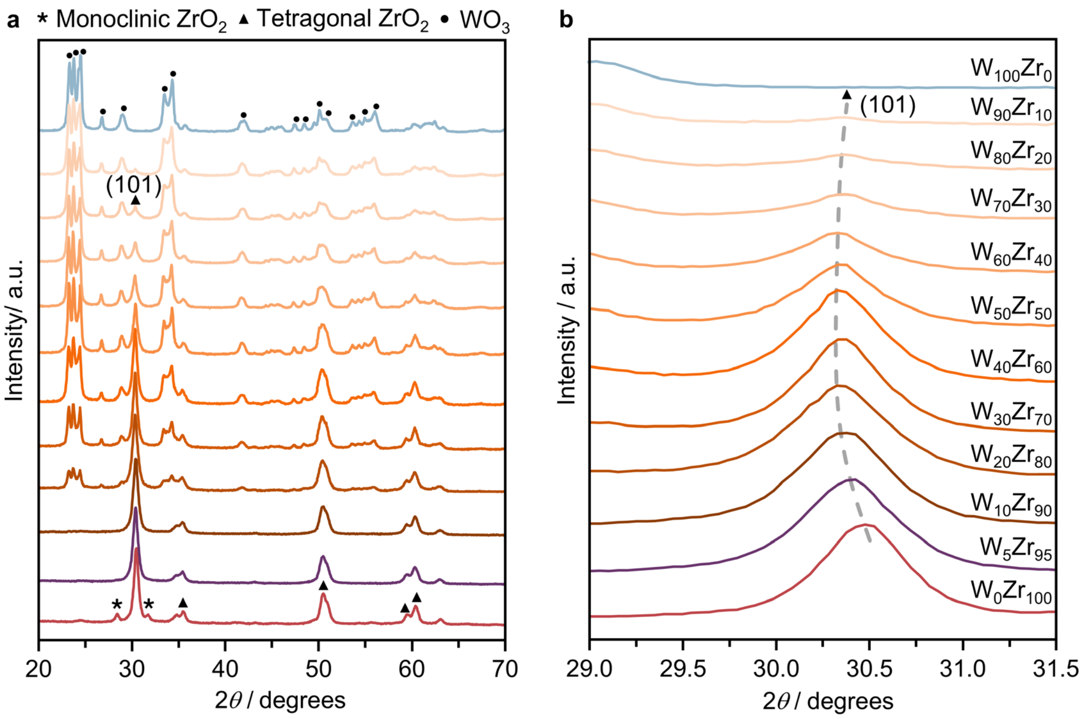

Supplementary Fig. 4 | Phase and crystallinity analysis of W_aZr_b catalysts. (**a**) XRD patterns of as-prepared W_aZr_b catalysts. (**b**) Enlarged XRD region around the reflection corresponding to tetragonal ZrO_2 (101).

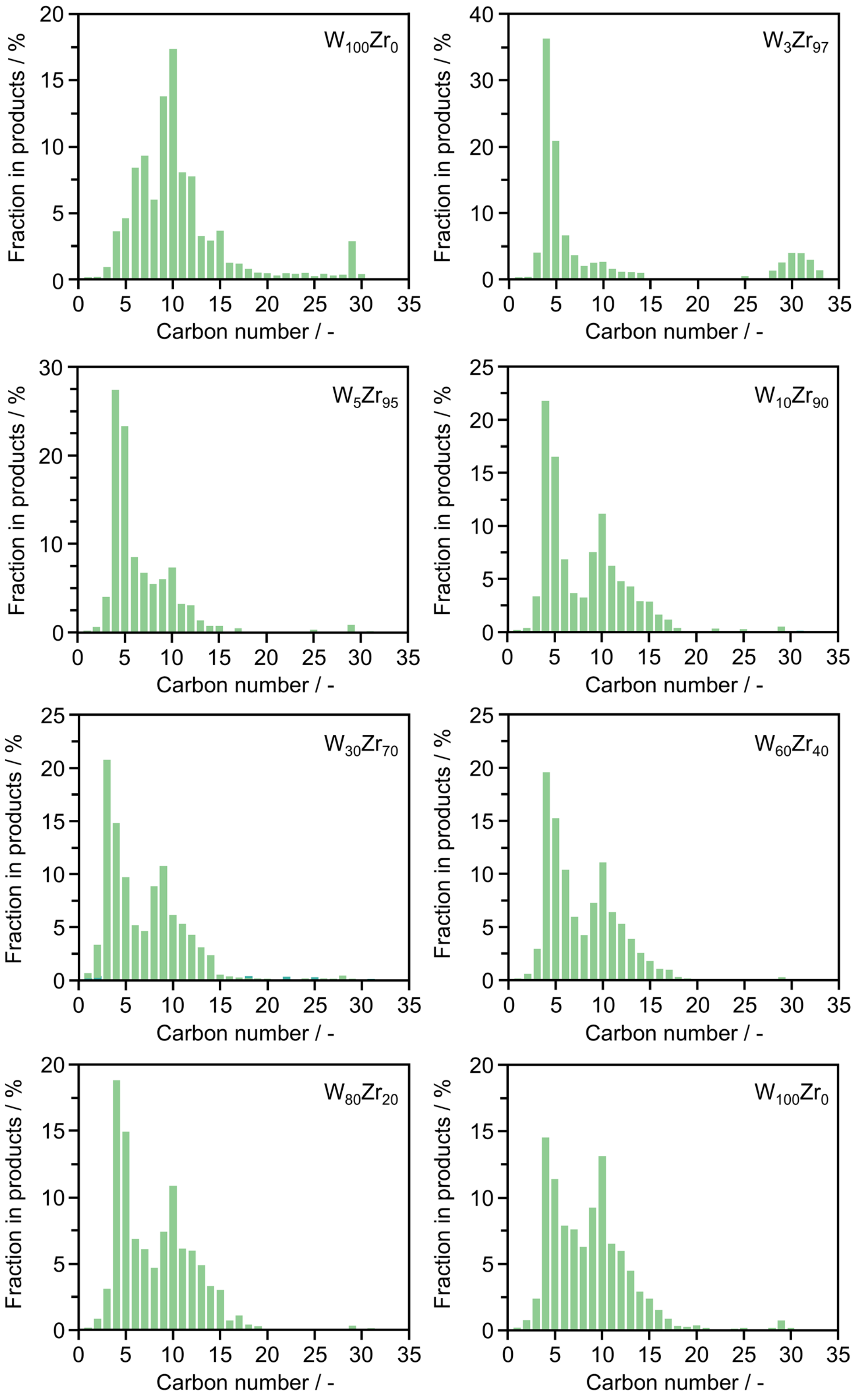

Supplementary Fig. 5 | Product distribution per chain length over W_aZr_b catalysts. Reaction conditions: 0.25 g catalyst, 0.5 g PP_{12k}, 4 bar N_2, 240 °C, 4 h, 750 rpm.

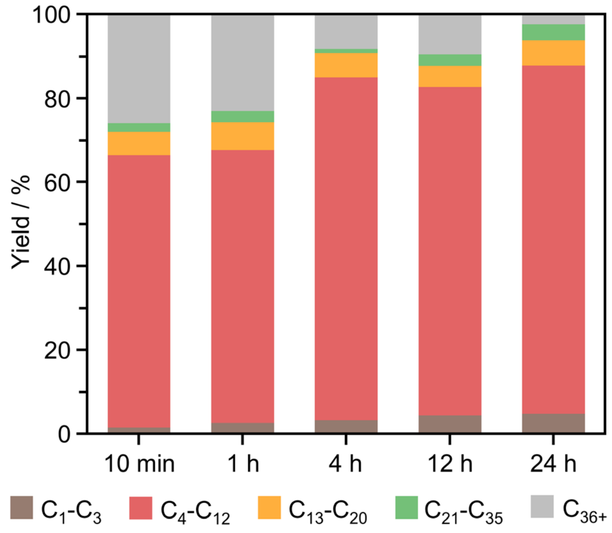

Supplementary Fig. 6 | Product distribution over $W_{10}Zr_{90}$ for different reaction times. Reaction conditions: 0.25 g catalyst, 0.5 g PP_{12k}, 4 bar N_2, 240 °C, 750 rpm.

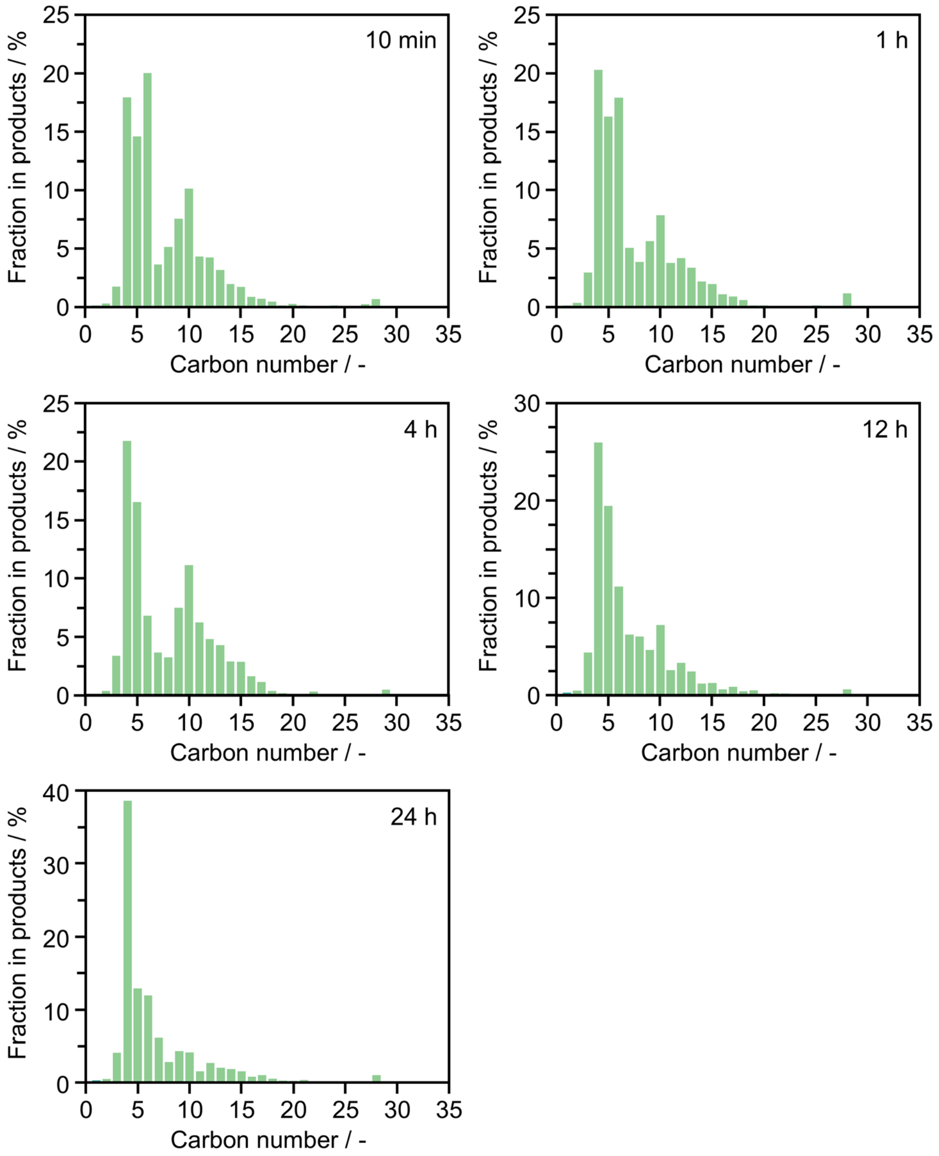

Supplementary Fig. 7 | Product distribution per chain length over $W_{10}Zr_{90}$ for different reaction times. Reaction conditions: 0.25 g catalyst, 0.5 g PP_{12k}, 4 bar N_2, 240 °C, 750 rpm.

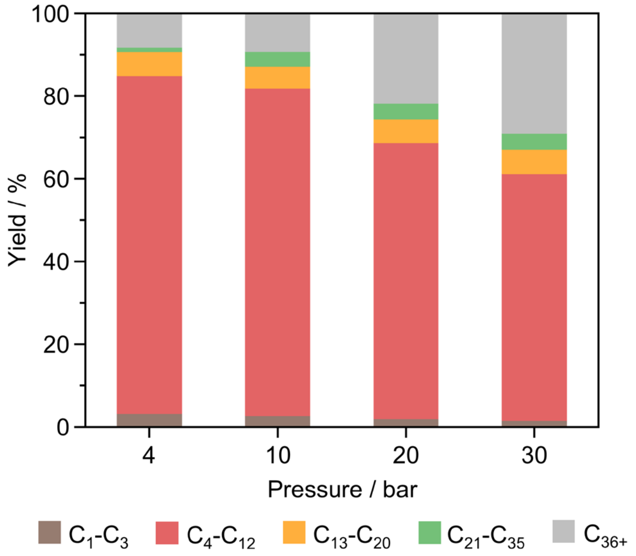

Supplementary Fig. 8 | Product distribution over $W_{10}Zr_{90}$ for different reaction pressures. Reaction conditions: 0.25 g catalyst, 0.5 g PP_{12k}, 240 °C, 4 750 rpm.

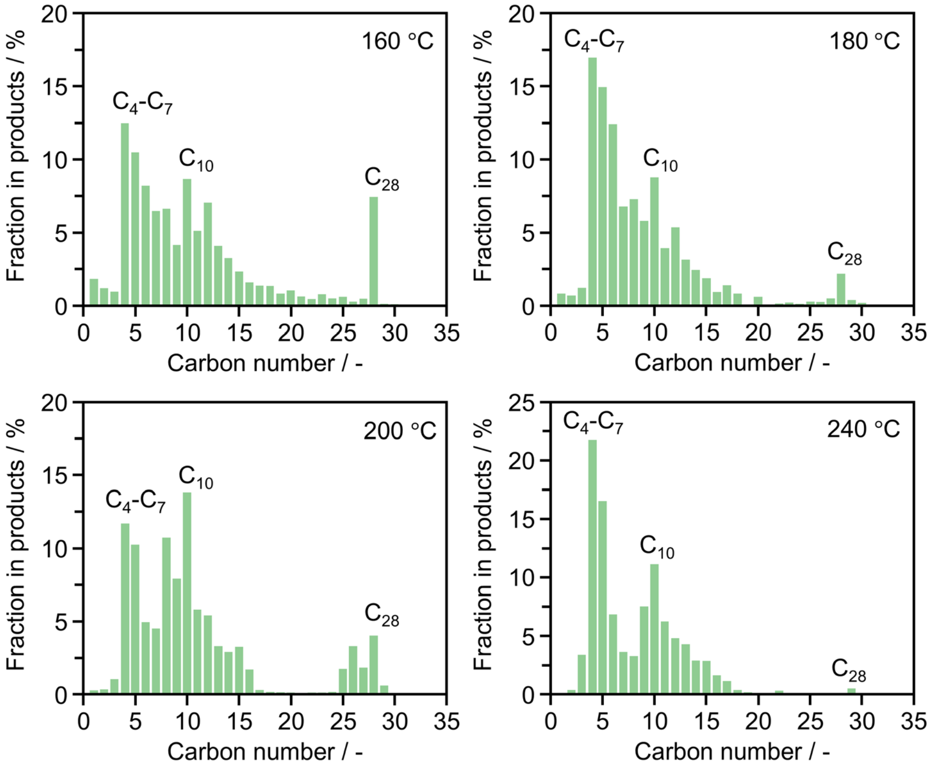

Supplementary Fig. 9 | Product distribution per chain length over $W_{10}Zr_{90}$ for different reaction temperatures. Reaction conditions: 0.25 g catalyst, 0.5 g PP_{12k}, 4 bar N_2, 4 h, 750 rpm.

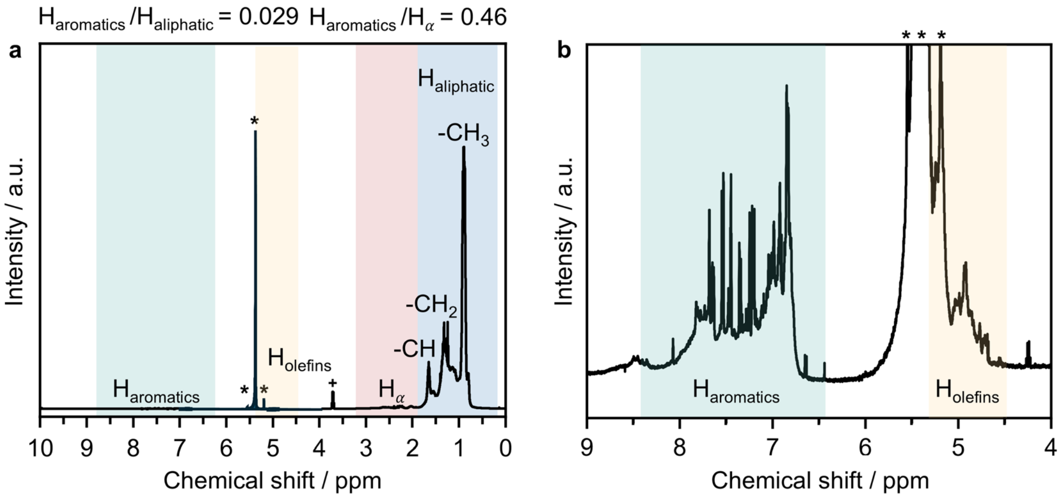

Supplementary Fig. 10 | Identification of families in liquid products. (**a**) ^{1}H NMR analysis of liquid products for $W_{10}Zr_{90}$. (**b**) Enlarged view around the aromatic and olefinic regions (chemical shift at 6.5-8.5 ppm and 4.5-5.4 ppm, respectively). *: dichloromethane solvent; +: water. Reaction conditions: 0.25 g catalyst, 0.5 g PP_{12k}, 4 bar N_2, 240 °C, 4 h, 750 rpm.

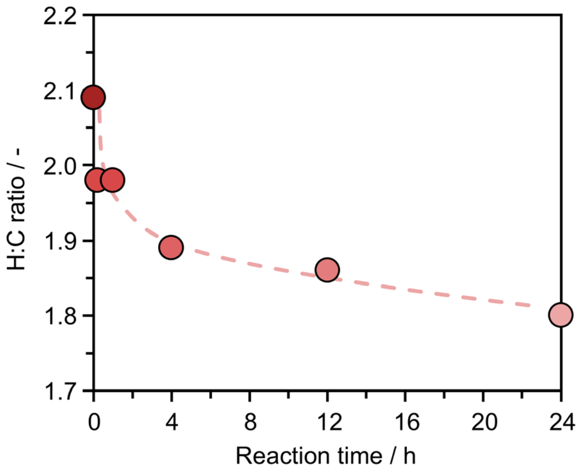

Supplementary Fig. 11 | Evolution of the H:C ratio of solid residues with reaction time for $W_{10}Zr_{90}$. Reaction conditions: 0.25 g catalyst, 0.5 g PP_{12k}, 4 bar N_2, 240 °C, 750 rpm.

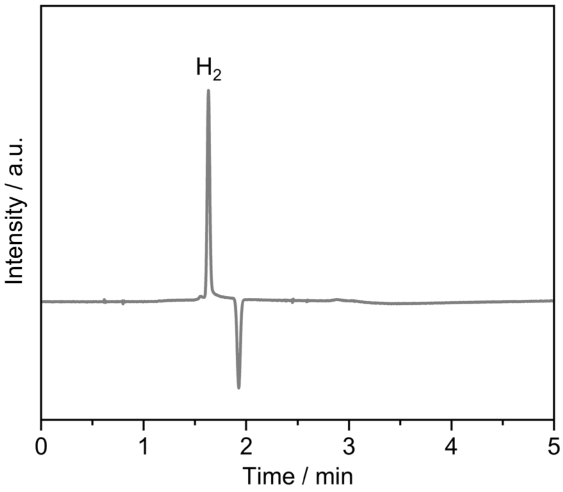

Supplementary Fig. 12 | GC-TCD signal for depolymerization of PP_{12k} over $W_{10}Zr_{90}$. Reaction conditions: 0.25 g catalyst, 0.5 g PP_{12k}, 4 bar N_2, 240 °C, 4 h, 750 rpm.

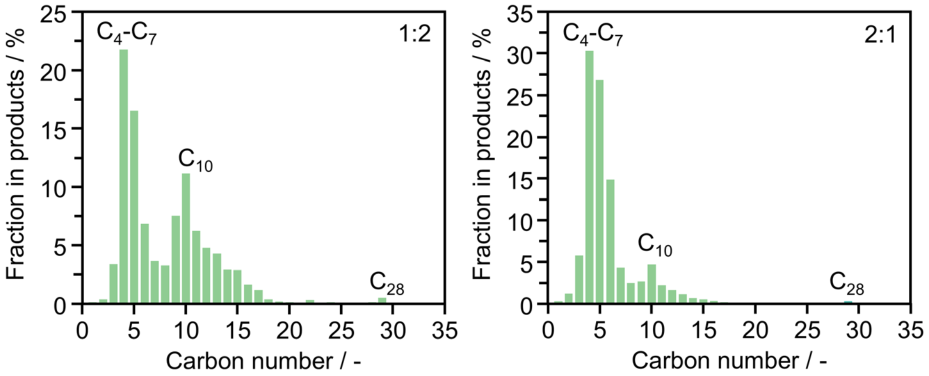

Supplementary Fig. 13 | Product distribution per chain length over $W_{10}Zr_{90}$ using different catalyst-to-plastic ratios. Reaction conditions: 4 bar N_2, 240 °C, 4 h, 750 rpm.

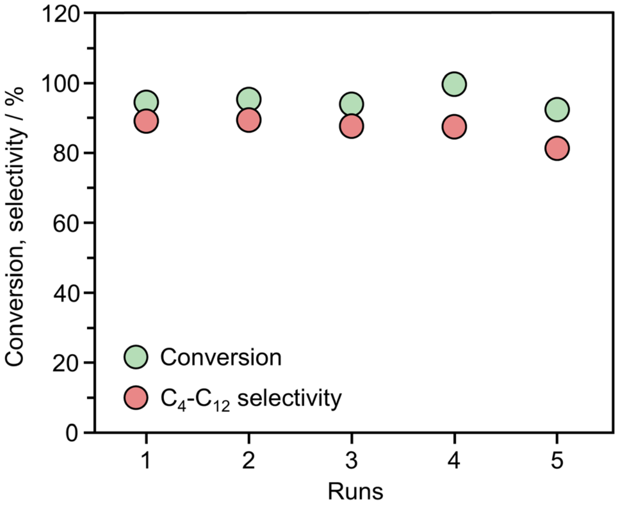

Supplementary Fig. 14 | Evolution of performance for depolymerization of PP_{12k} over $W_{10}Zr_{90}$ during consecutive catalytic tests. Reaction conditions: 0.25 g catalyst, 0.5 g PP_{12k}, 4 bar N_2, 240 °C, 4 h each, 750 rpm.

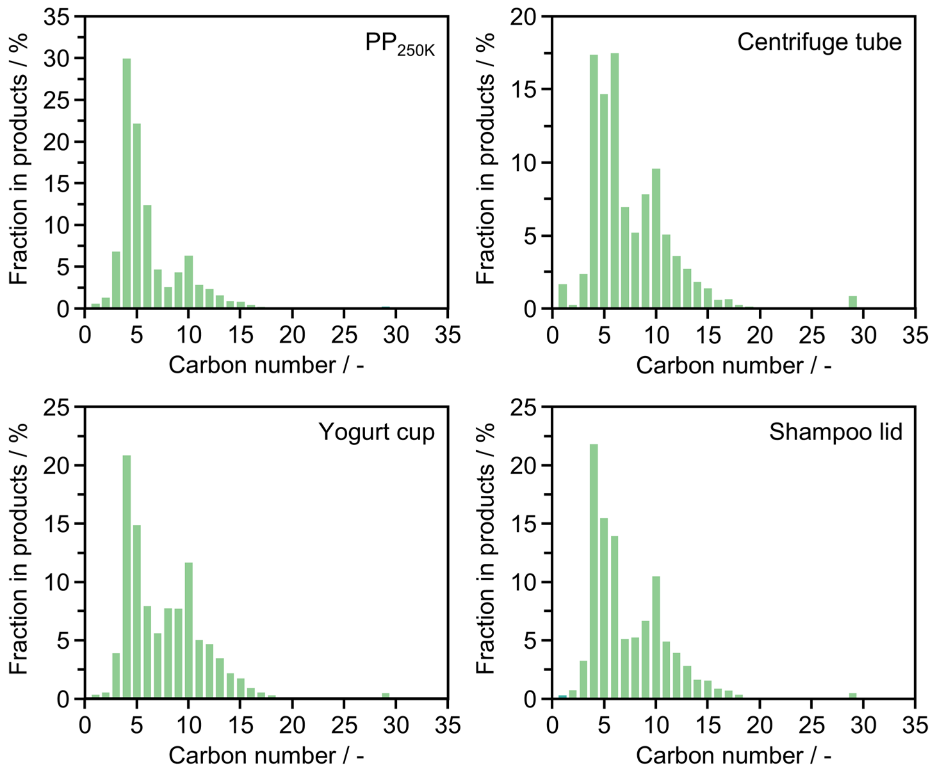

Supplementary Fig. 15 | Product distribution per chain length over $W_{10}Zr_{90}$ for the processing of different PP-based items. Reaction conditions for each case are provided in **Supplementary Table ~~7~~8**.

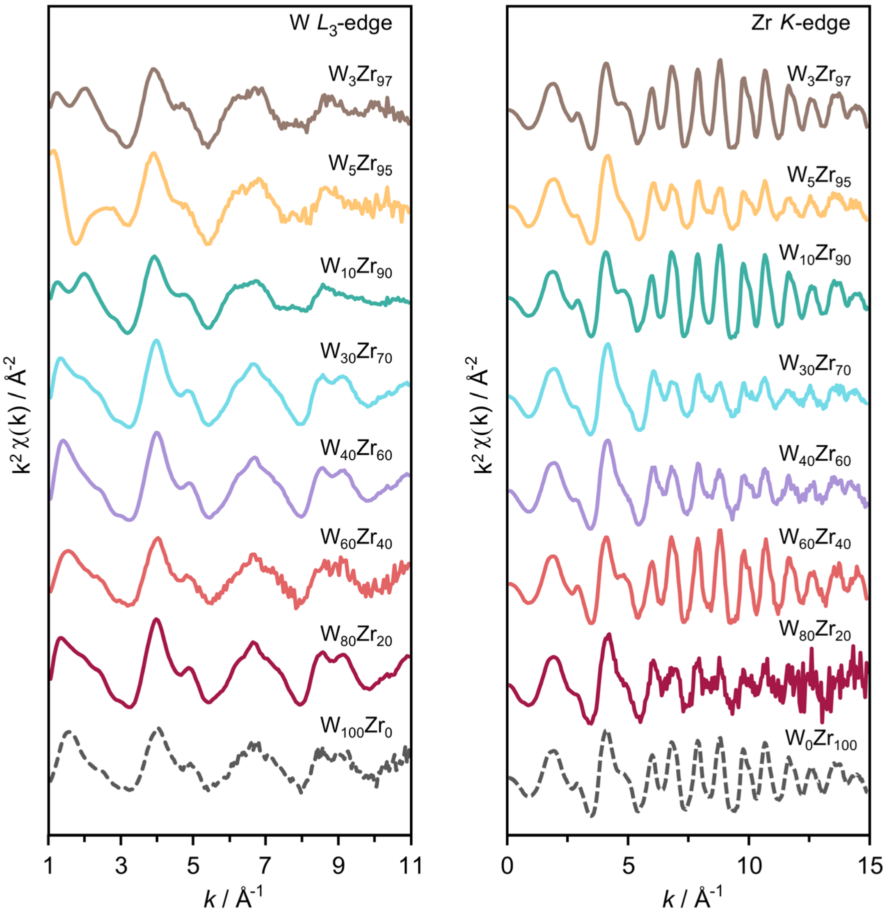

Supplementary Fig. 16 | W L_3-edge and Zr K-edge EXAFS spectra of representative W_aZr_b catalysts in k-space.

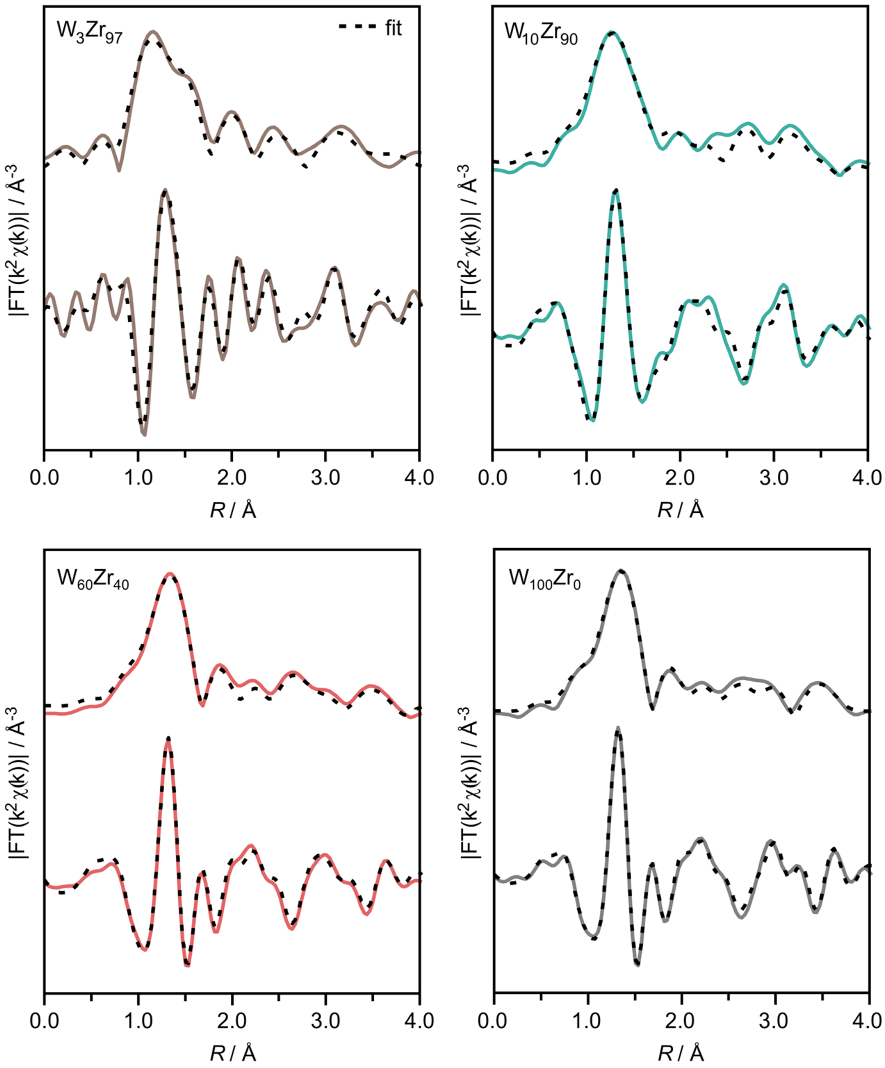

Supplementary Fig. 17 | W L_3-edge EXAFS fitting for representative W_aZr_b catalysts in *R*-space with magnitude and imaginary part.

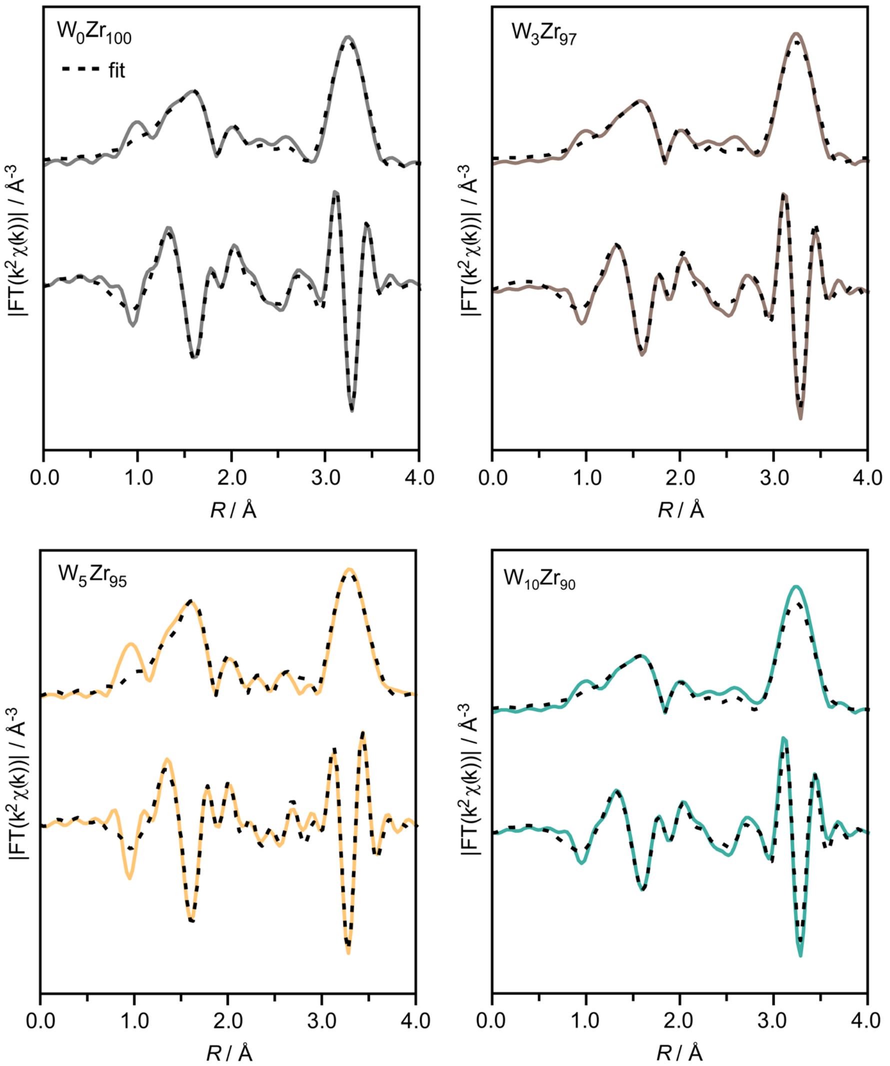

Supplementary Fig. 18 | Zr *K*-edge EXAFS fitting for representative W_aZr_b catalysts in *R*-space with magnitude and imaginary part.

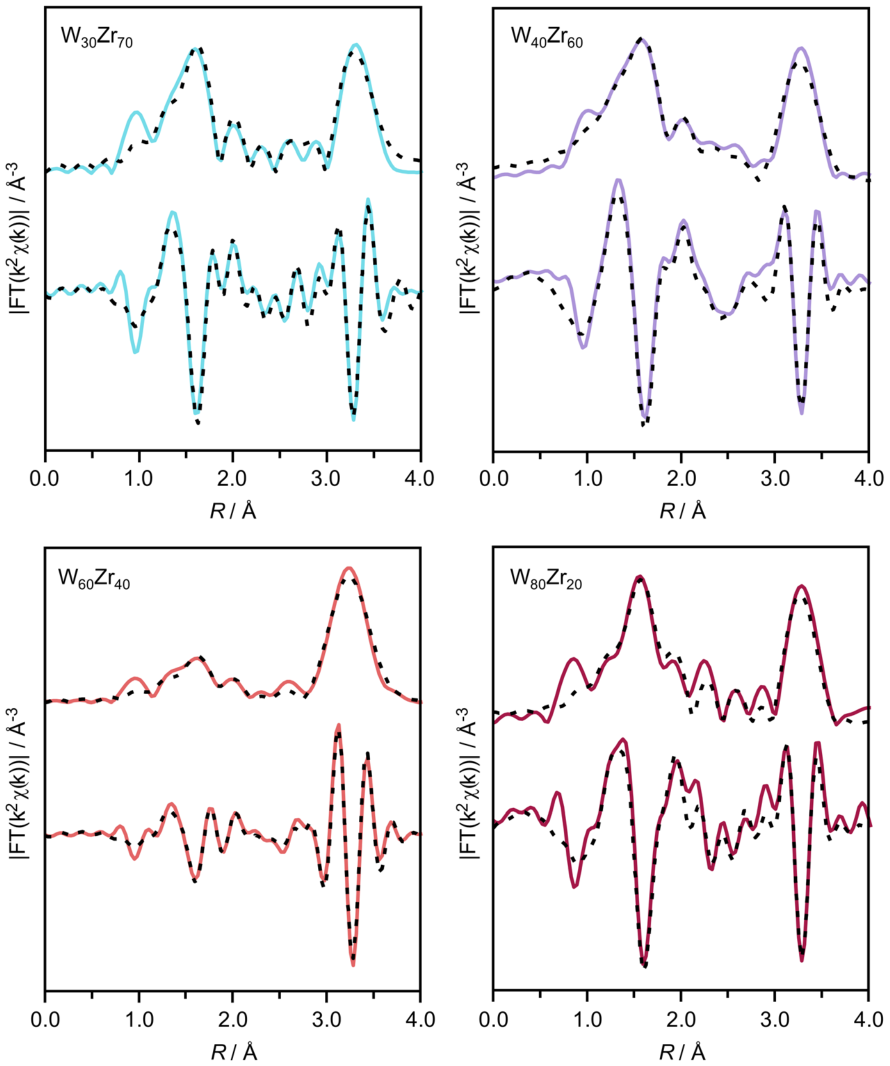

Supplementary Fig. 18 (continued).

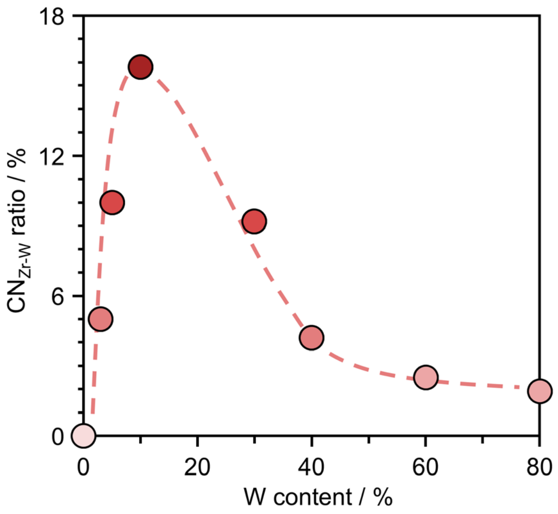

Supplementary Fig. 19 | Correlation between the $CN_{Zr\text{-}W}$ ratio and the W content in W_aZr_b catalysts.

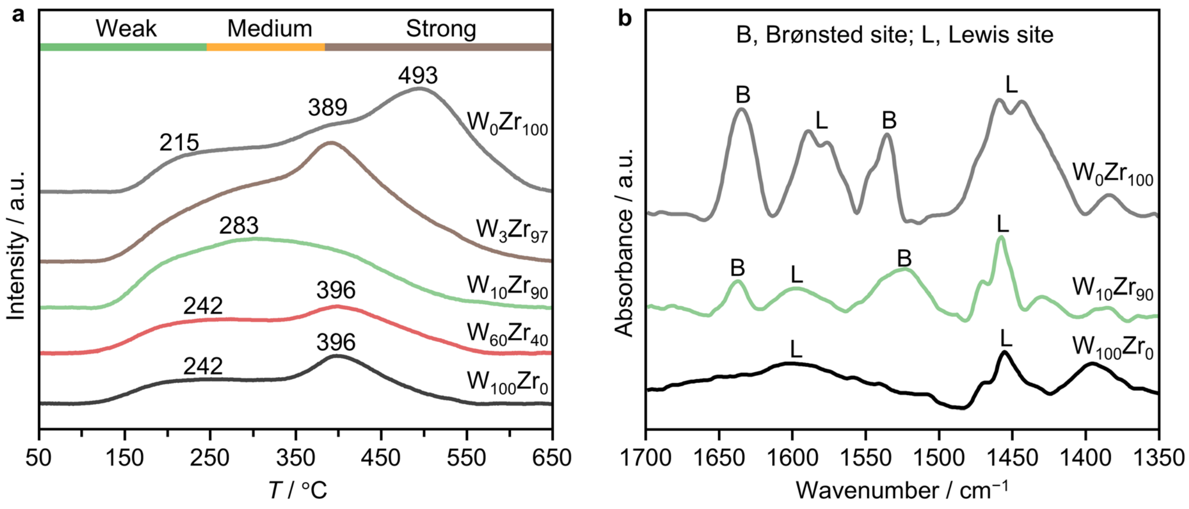

Supplementary Fig. 20 | Acidity analysis of as-prepared W_aZr_b catalysts. (**a**) NH_3-TPD profiles and (**b**) pyridine FTIR spectra.

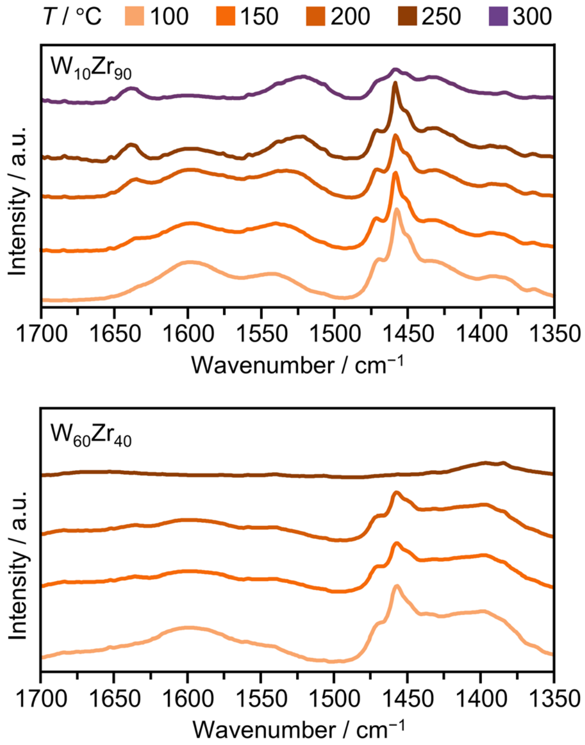

Supplementary Fig. 21 | Pyridine desorption FTIR analysis for selected catalysts at different temperatures.

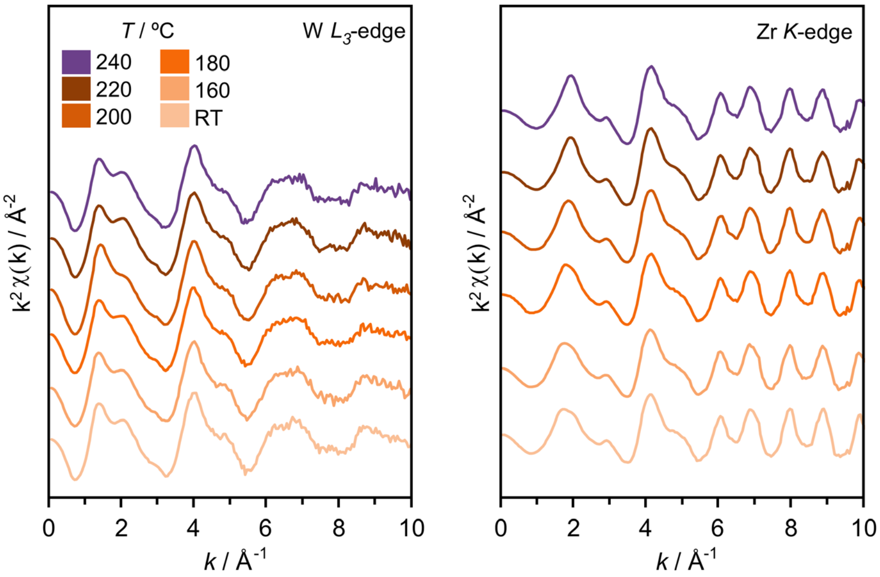

Supplementary Fig. 22 | *Operando* W L_3-edge and Zr *K*-edge EXAFS spectra of $W_{10}Zr_{90}$ catalysts in *k*-space.

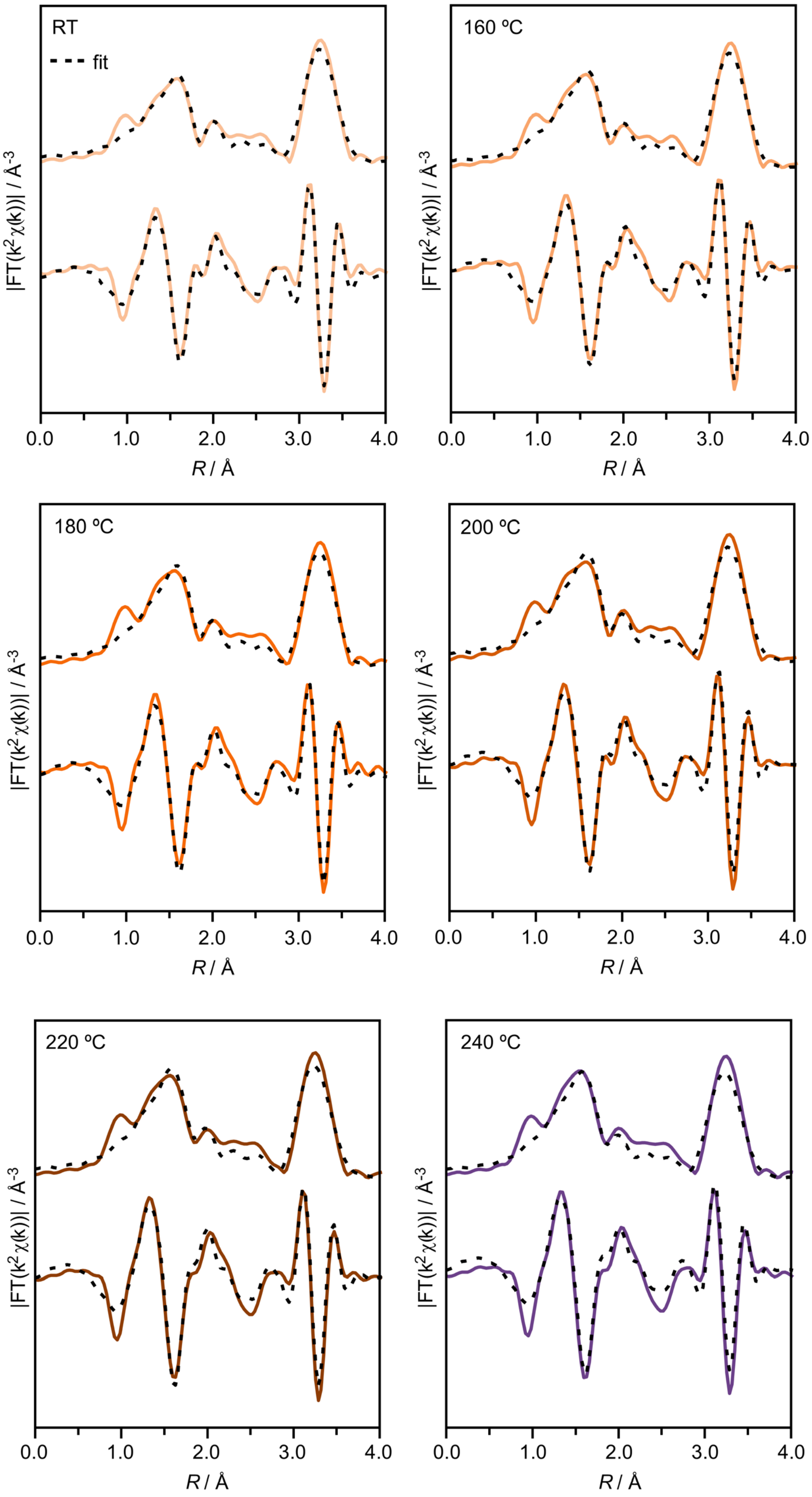

Supplementary Fig. 23 | *Operando* Zr *K*-edge EXAFS fitting for $W_{10}Zr_{90}$ catalysts in *R*-space with magnitude and imaginary part.

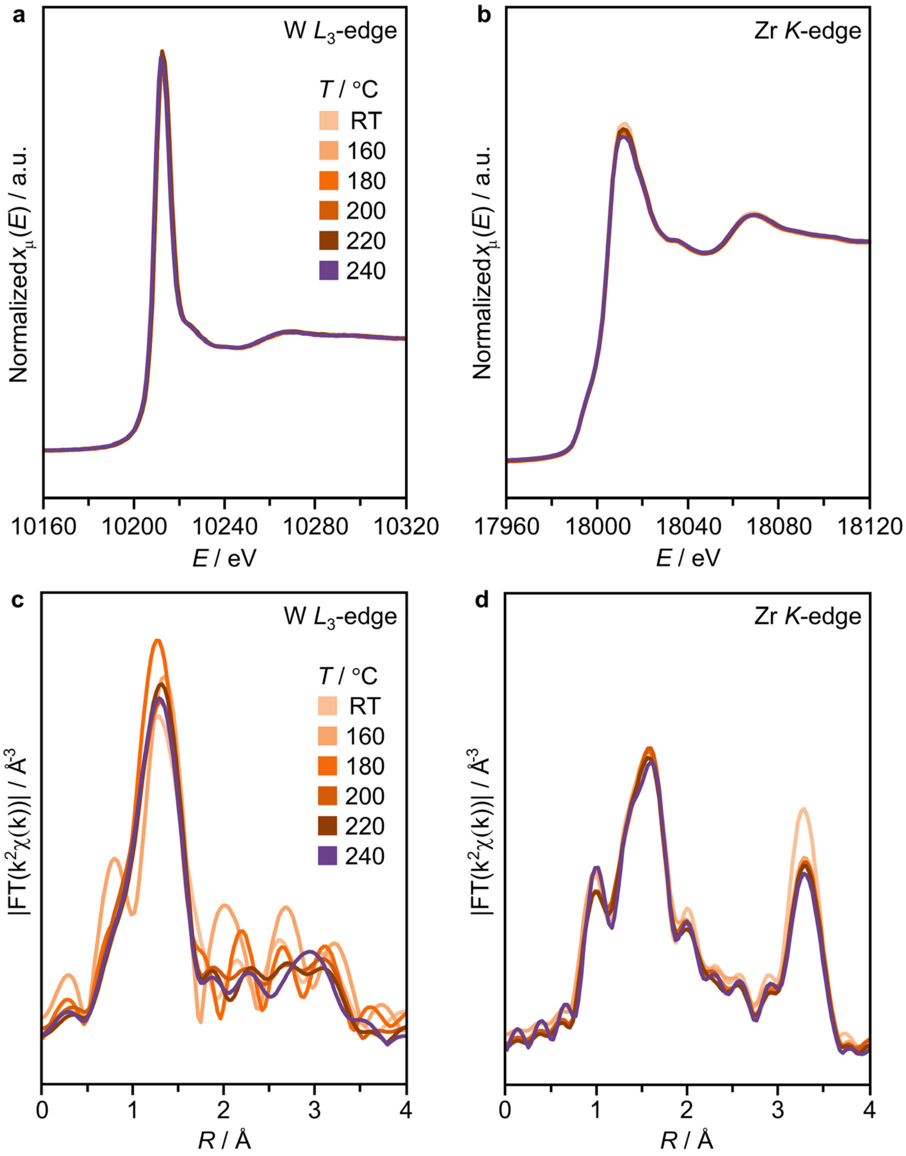

Supplementary Fig. 24 | *Operando* XAS analysis of W_3Zr_{97} under different temperatures. Evolution of the (**a**) W L_3-edge and (**b**) Zr *K*-edge XANES. (**c**) W L_3-edge and (**d**) Zr *K*-edge EXAFS. Reaction conditions: 0.25 g catalyst, 0.5 g PP_{12k}, 0.5 h each, 4 bar He, no stirring. RT stands for room temperature.

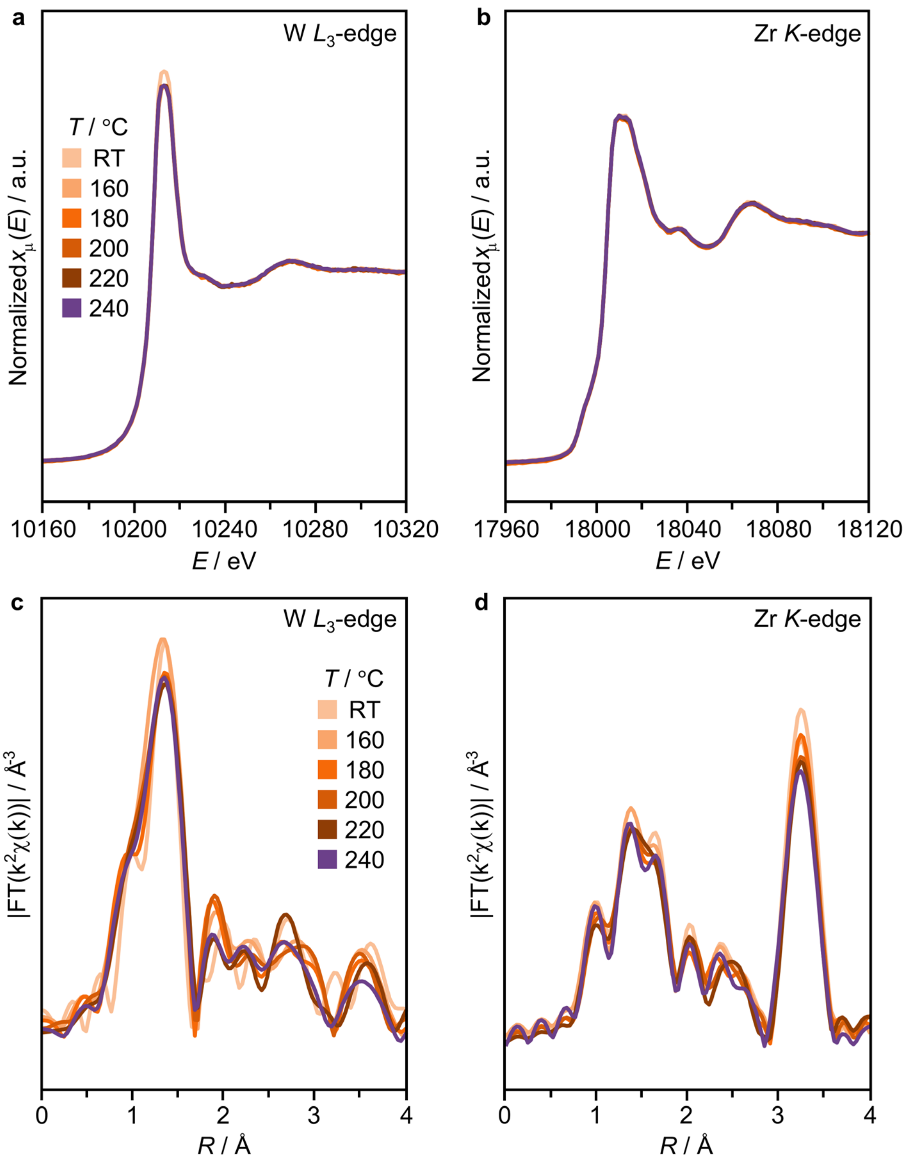

Supplementary Fig. 25 | *Operando* XAS analysis of $W_{60}Zr_{40}$ under different temperatures. Evolution of the (**a**) W L_3-edge and (**b**) Zr *K*-edge XANES. (**c**) W L_3-edge and (**d**) Zr *K*-edge EXAFS. Reaction conditions: 0.25 g catalyst, 0.5 g PP_{12k}, 0.5 h each, 4 bar He, no stirring. RT stands for room temperature.

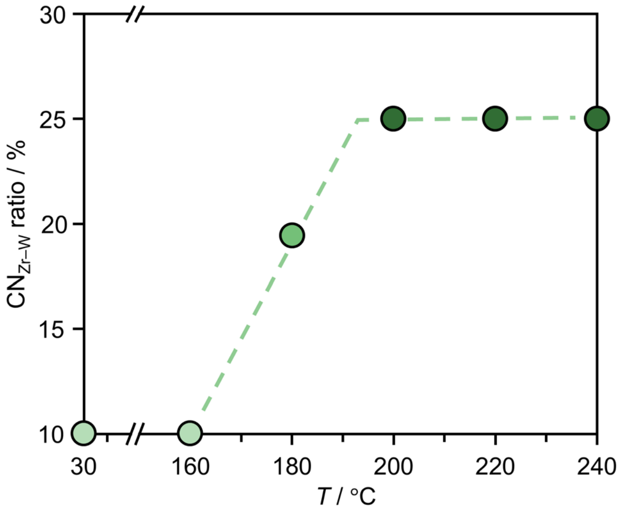

Supplementary Fig. 26 | Evolution of the $CN_{Zr–W}$ ratio with reaction temperature for $W_{10}Zr_{90}$.

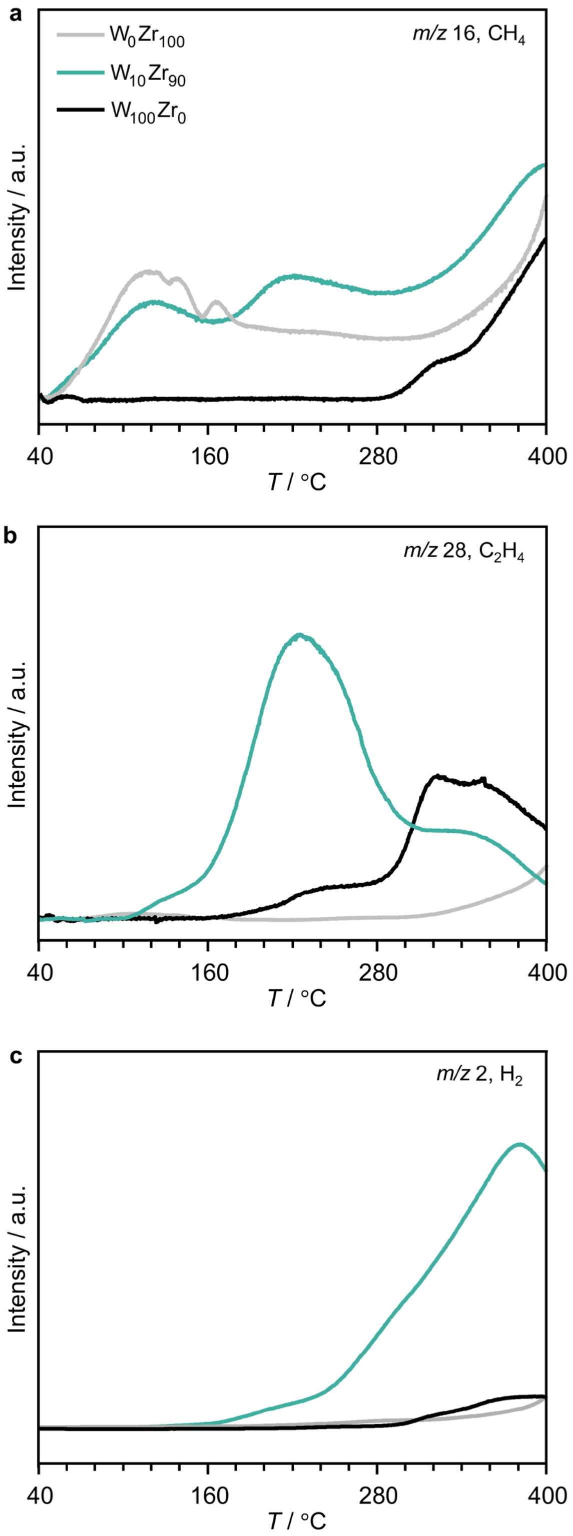

Supplementary Fig. 27 | TPSR-MS profiles from W_0Zr_{100}, $W_{10}Zr_{90}$, and $W_{100}Zr_0$ for depolymerization of PP_{12k} with signal evolution for (**a**) *m/z* 16 for methane, (**b**) *m/z* 28 for ethylene, (**c**) *m/z* 2 for hydrogen.

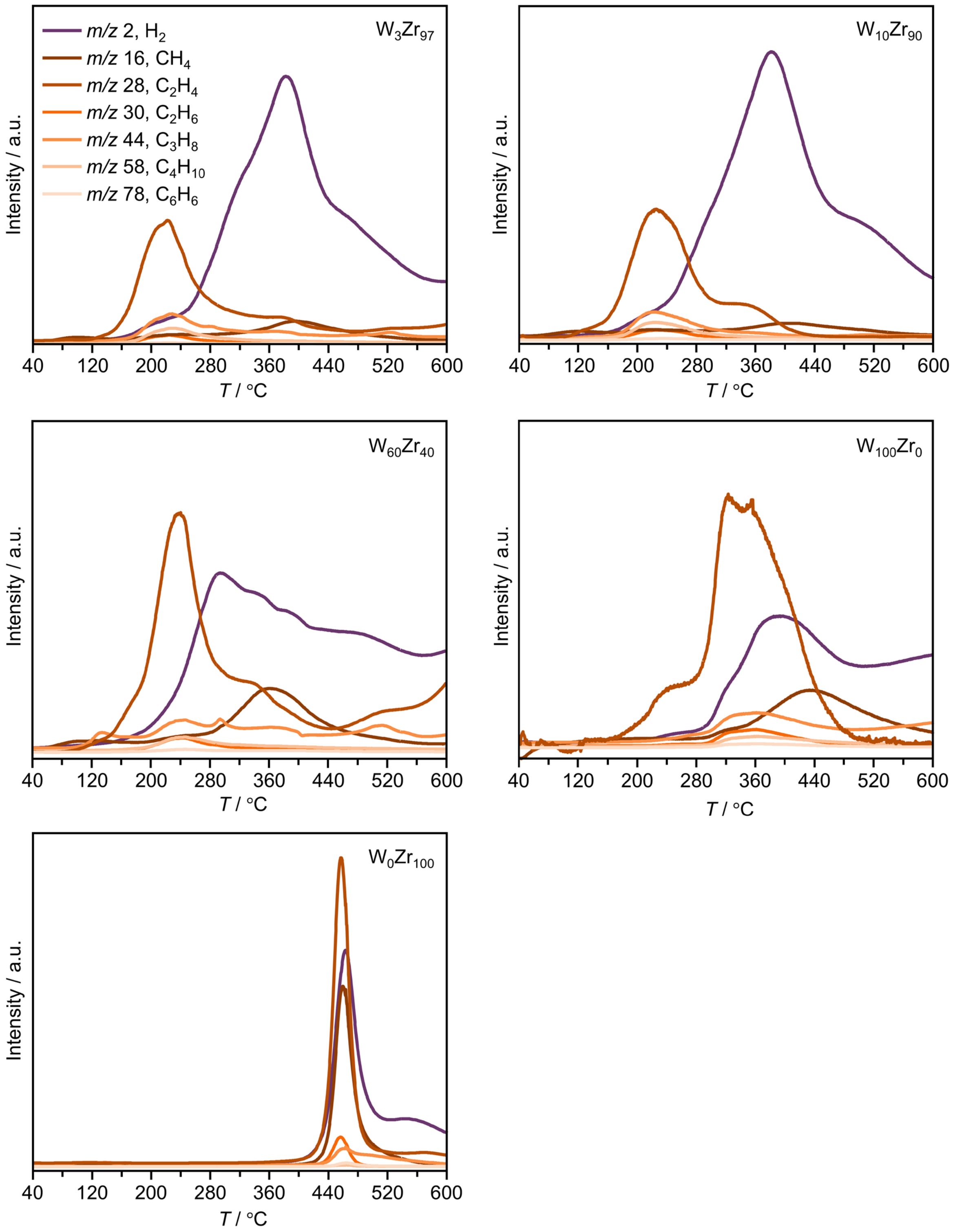

Supplementary Fig. 28 | TPSR-MS profiles from the depolymerization of PP_{12k} over W_aZr_b catalysts for selected products.

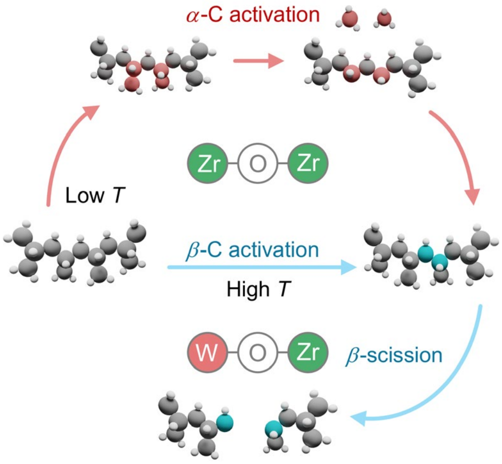

Supplementary Fig.29 | Schematic of the proposed C–C activation pathways for the depolymerization of PP over $W_{10}Zr_{90}$.

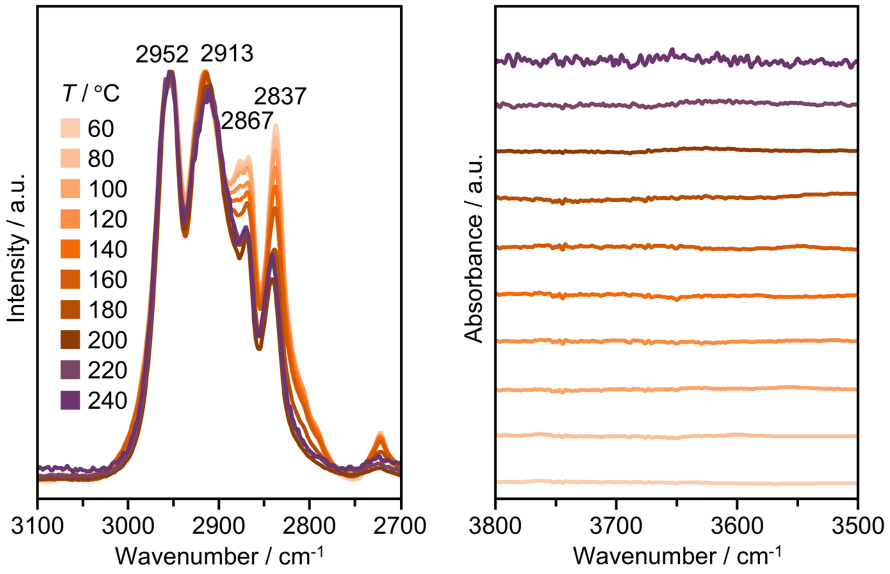

Supplementary Fig. 30 | *Operando* DRIFTS during the depolymerization of PP_{12k} over $W_{100}Zr_0$. Evolution of PP C–H stretching (2700-3100 cm^{-1}) and the OH stretching (3500-3800 cm^{-1}) bands with reaction temperature.

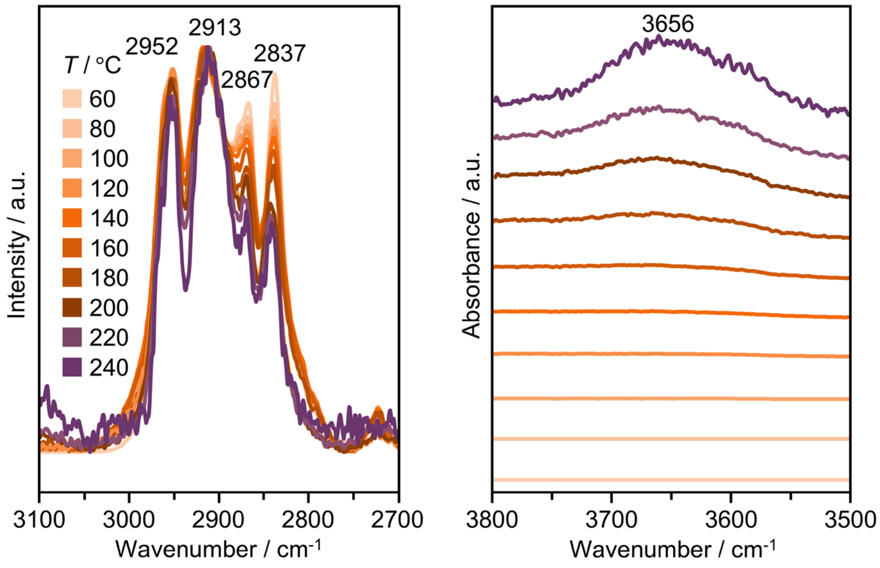

Supplementary Fig. 31 | *Operando* DRIFTS during the depolymerization of PP_{12k} over $W_{60}Zr_{40}$. Evolution of PP C–H stretching (2700-3100 cm^{-1}) and the OH stretching (3500-3800 cm^{-1}) bands with reaction temperature.

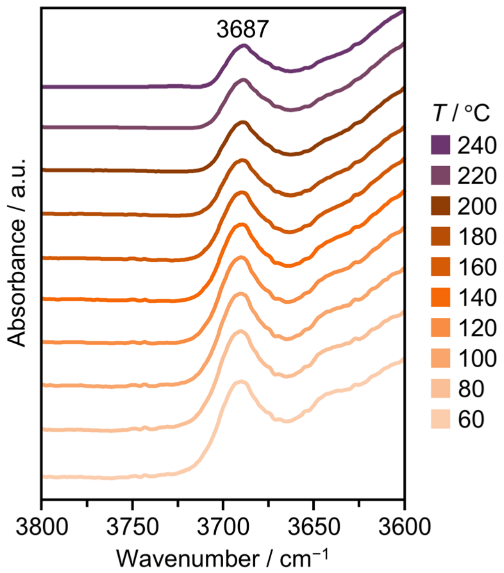

Supplementary Fig. 32 | *Operando* DRIFTS during the depolymerization of PP_{12k} over W_0Zr_{100}. Evolution of the OH stretching band with reaction temperature.

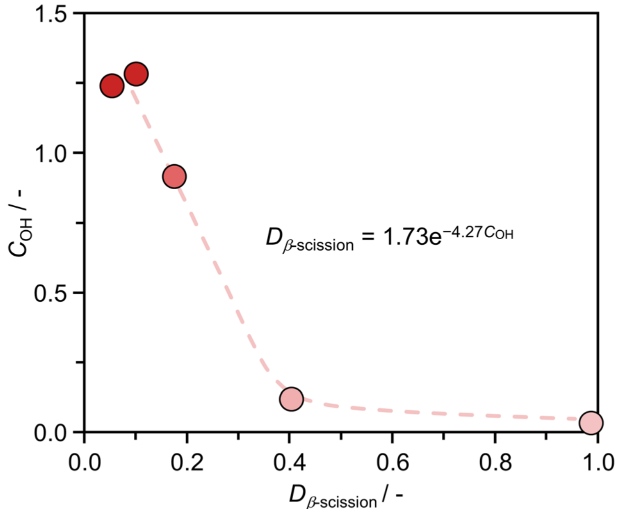

Supplementary Fig. 33 | Structure-performance correlation obtained for $W_{10}Zr_{90}$. Plot of OH concentration (C_{OH}, calculated as the integrated area in *operando* DRIFTS experiments, **Fig. 6b**) versus the β-scission degree ($D_{\beta\text{-scission}}$, calculated from the C_2H_4 normalized intensity in MS signal, **Fig. 6a**).

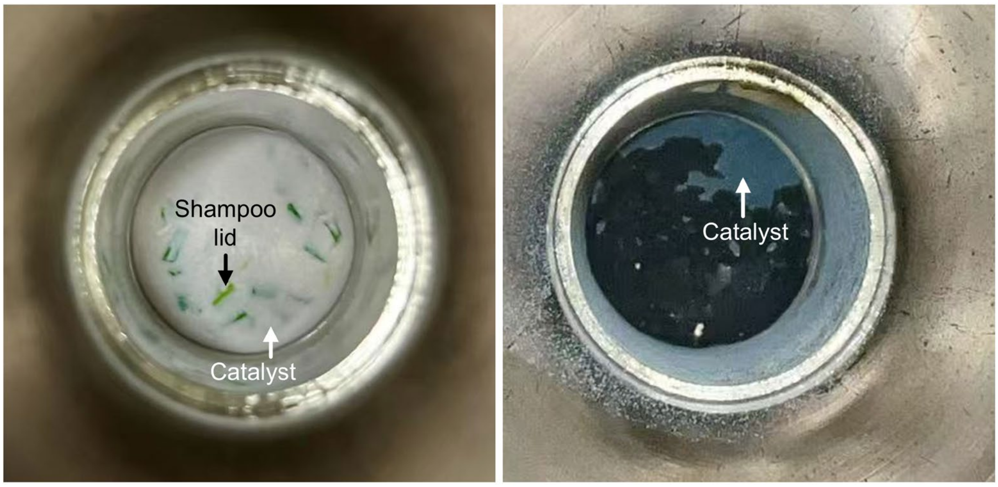

Supplementary Fig. 34 | Photographs of the interior of the reactor before and after reaction. The white phase before reaction and the blue phase after reaction correspond to the $W_{10}Zr_{90}$ catalyst. Reaction conditions: 0.5 g shampoo lid, 0.25 g catalyst, 240 ºC, 4 h, 4 bar N_2, 750 rpm.

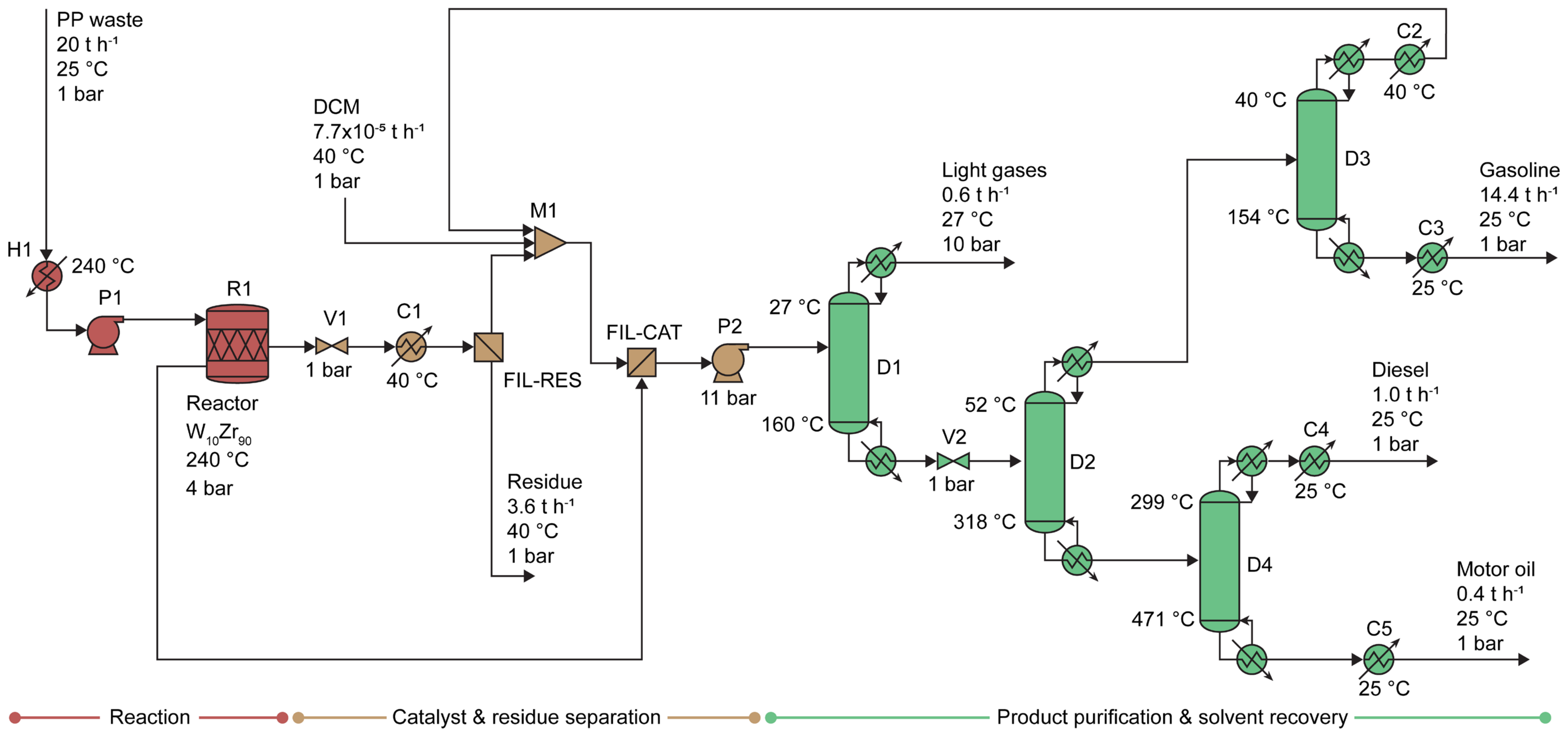

Supplementary Fig. 35 | Detailed process flowsheet for the hydrogen-free depolymerization of PP items over $W_{10}Zr_{90}$. The system is scaled to recycle 20 t_{PP} h^{-1}, including downstream catalyst recycle and product separation. The selectivity and conversion achieved in the reactor towards specific products depending on the plastic source are described in **Supplementary Table 8.** The values in the figure correspond to the shampoo lids scenario.

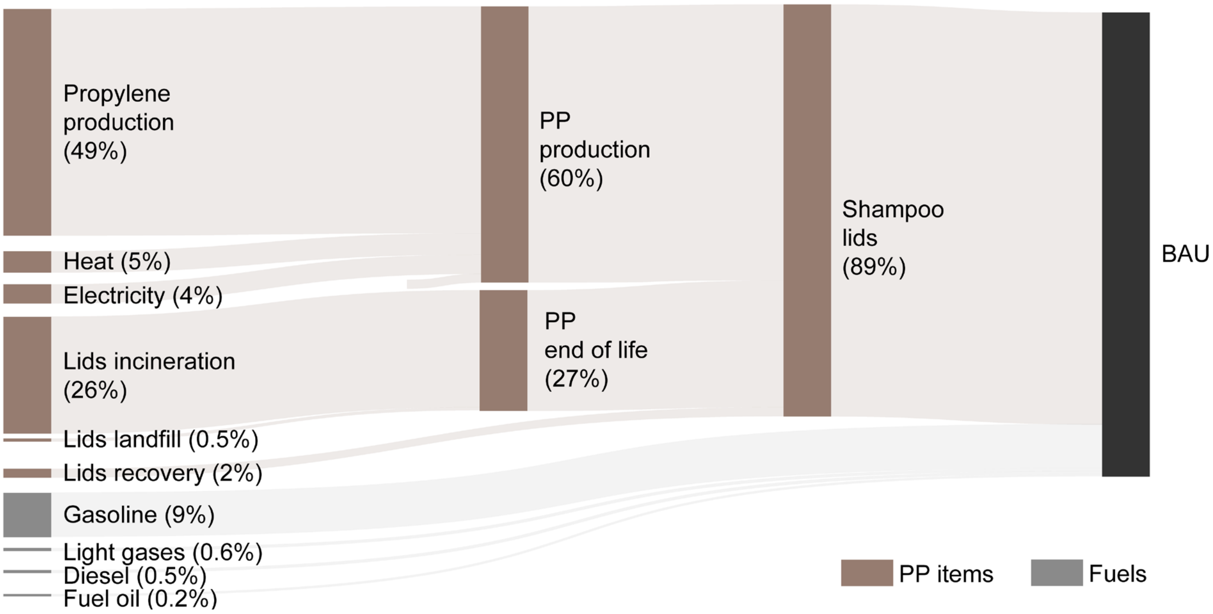

Supplementary Fig. 36 | Contributions to the GWP of the hydrogen-free chemical recycling of PP items over $W_{10}Zr_{90}$. The Sankey diagram shows the contributions to the GWP calculation for the representative shampoo lid scenario, including the production of PP and the supply of materials and energy required for the recycling of PP waste. Background data comes from Ecoinvent 3.10.[22]

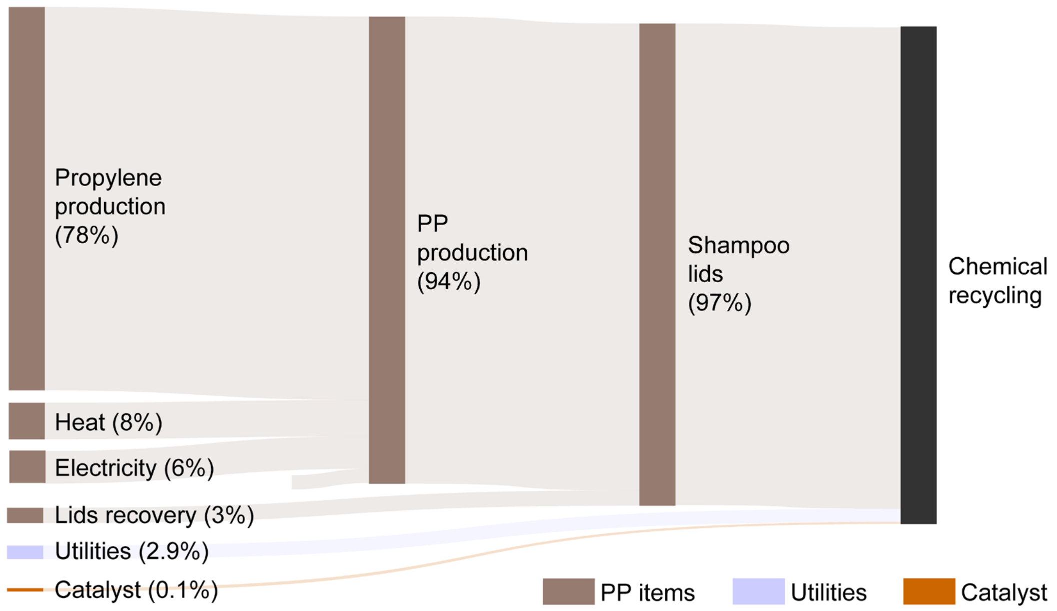

Supplementary Fig. 37 | Contributions to the GWP of the BAU case. The Sankey diagram shows the contributions to the GWP calculation of PP production and end-of-life treatment, together with the production of fuels from fossil resources equivalent to the portfolio produced from the representative case of hydrogen-free chemical recycling of shampoo lids over $W_{10}Zr_{90}$. Background data comes from Ecoinvent 3.10.[22]

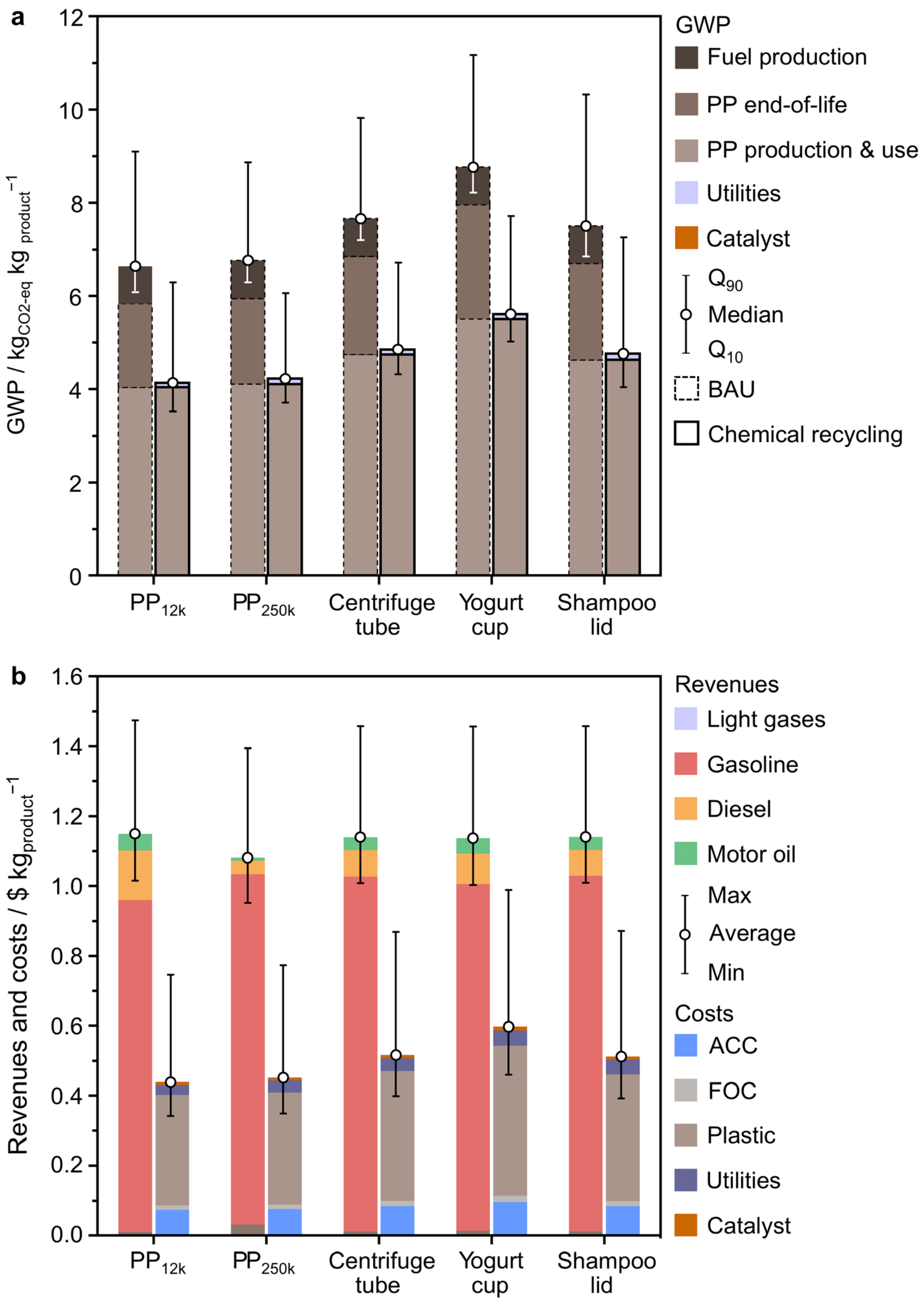

Supplementary Fig. 38 | Economic and environmental analysis of hydrogen-free chemical recycling of PP consumer goods over $W_{10}Zr_{90}$. (**a**) GWP versus equivalent BAU fossil production and (**b**) production cost of the chemical recycling process and revenues from product selling at market prices. Utilities group cooling, heating, and electricity demands, together with solvent make-up and residue disposal. Uncertainty for cost, revenues, and GWP is represented by error bars. FOC stands for fixed operating costs, ACC for annualized capital cost. Uncertainty methodology giving rise to the Min-Average-Max range is explained in the Methods section.

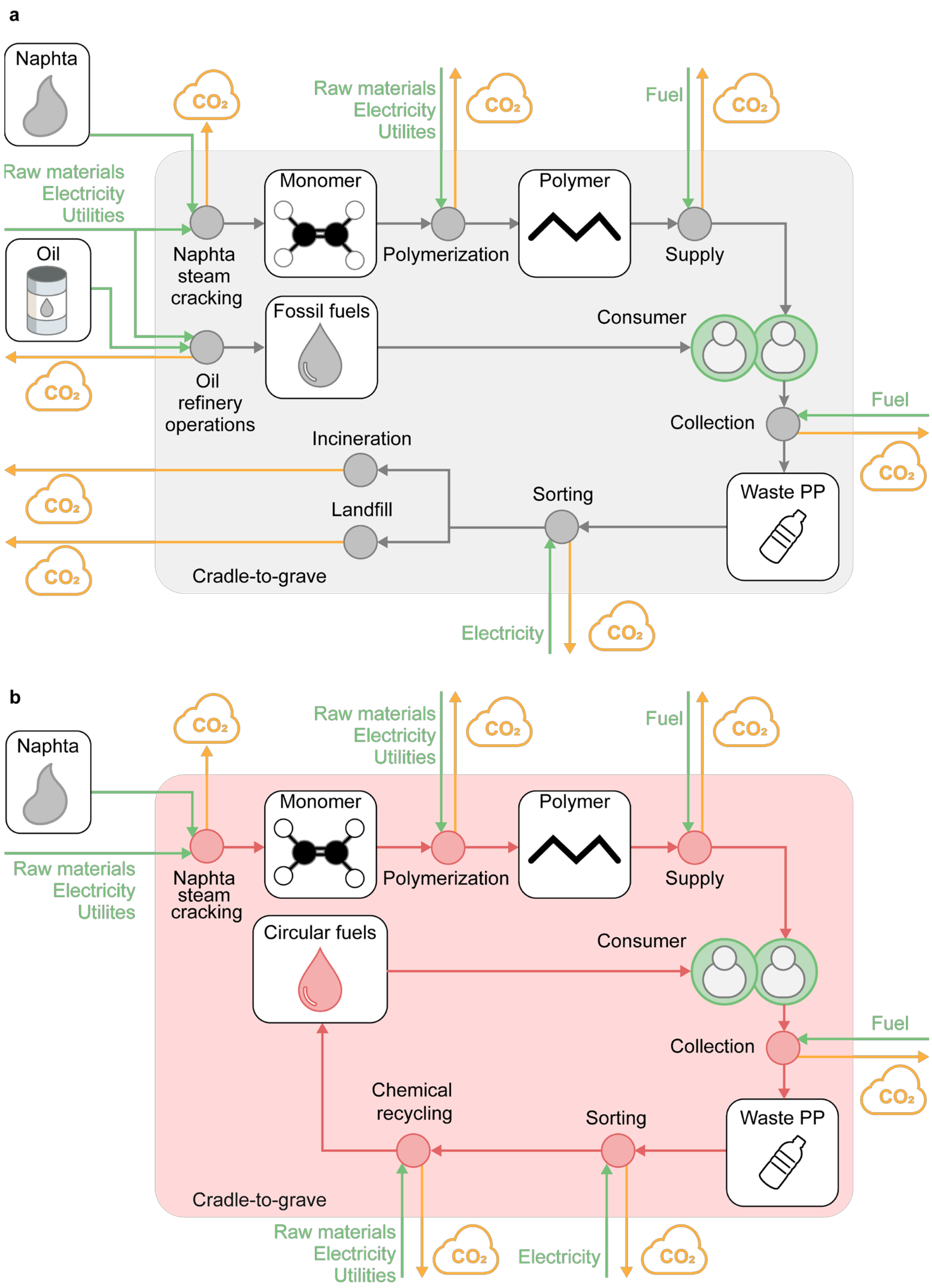

Supplementary Fig. 39 | System boundaries definition for the (**a**) business-as-usual (BAU) and (**b**) H_2-free scenarios. Both systems produce the same portfolio of fuels while handling waste PP by conventional end-of-life alternatives (BAU) or by chemical recycling (H_2-free). The colored boxes denote system boundaries, specifying the interface with the global market (background system) through which mass and energy flows are exchanged

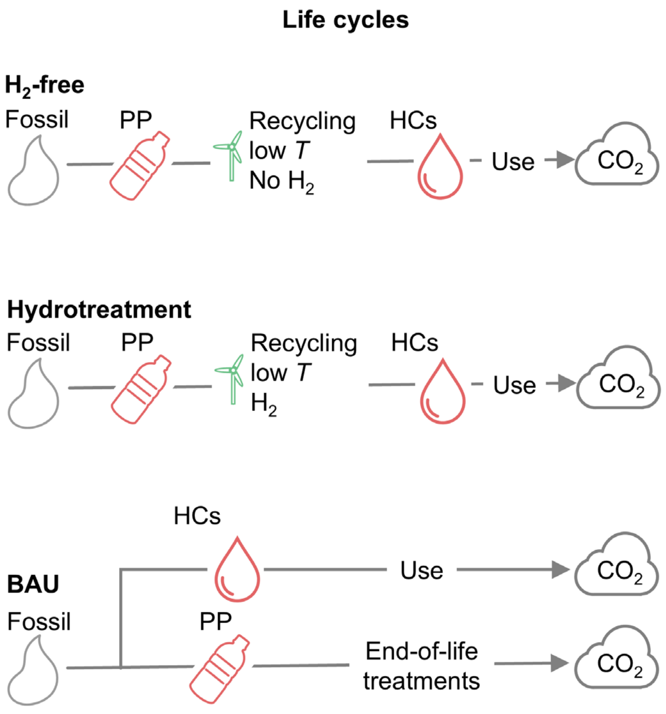

Supplementary Fig. 40 | Schemes of life cycles for the polyolefin recycling technologies considered in **Fig. 8** and for the BAU production of polyolefins and liquid hydrocarbons (HCs).

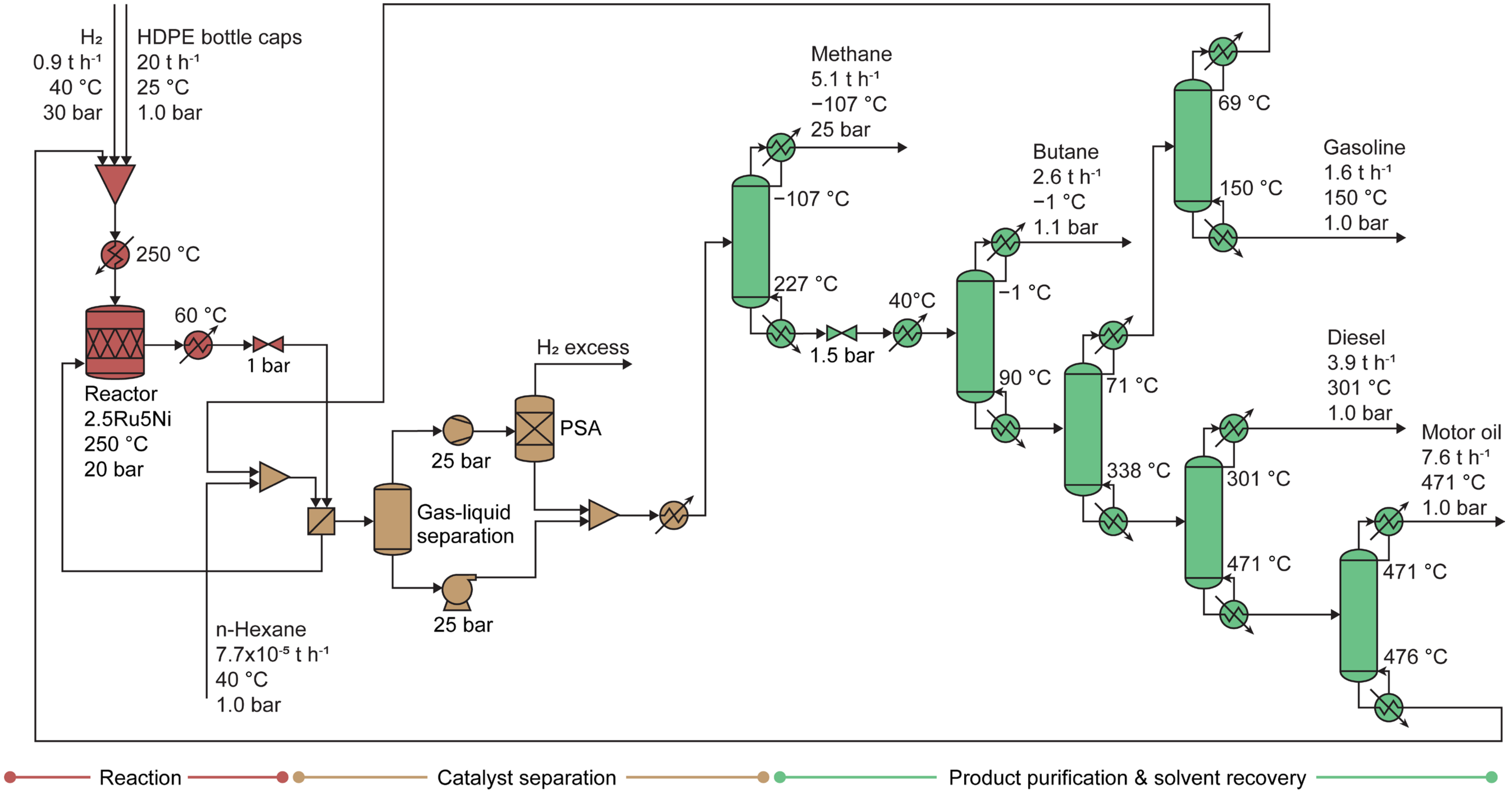

Supplementary Fig. 41 | Process flowsheet for the chemical recycling of polyolefins by hydrotreatment. The system is scaled to recycle 20 t h^{-1} of waste plastic. The selectivity and conversion achieved in the reactor towards specific products, depending on the plastic source and catalyst, are described in **Supplementary Table 21.** The values in the figure correspond to those reported by Nogueroles-Langa *et al.*[24] For simplicity, final product cooling is omitted.

Supplementary References

1. Ross-Medgaarden, E. I. & Wachs, I. E. Structural determination of bulk and surface tungsten oxides with UV−vis diffuse reflectance spectroscopy and Raman spectroscopy. *J. Phys. Chem. C* **111**, 15089–15099 (2007).
2. Ross-Medgaarden, E. I. *et al.* New insights into the nature of the acidic catalytic active sites present in ZrO_2-supported tungsten oxide catalysts. *J. Catal.* **256**, 108–125 (2008).
3. Han, W. *et al.* One-pot catalytic conversion of polyethylene wastes to gasoline through a dual-catalyst system. *Chem* **11**, 102340 (2025).
4. Hu, Q. *et al.* Polyethylene hydrogenolysis by dilute RuPt alloy to achieve H_2-pressure-independent low methane selectivity. *Nat. Commun.* **15**, 10573 (2024).
5. Vollmer, I. *et al.* Unravelling potential reaction intermediates during catalytic pyrolysis of polypropylene with microscopy and spectroscopy. *Catal. Sci. Technol.* **14**, 894–902 (2024).
6. Towler, G. & Sinnott, R. K. *Chemical engineering design*. (Butterworth-Heinemann, 2021).
7. Olmstead, I. Cost benefit analysis of dewatering abattoir sludge using three-way decanters. (2014). Available at: https://shorturl.at/2XiKy.
8. Salah, C., Cobo, S., Pérez-Ramírez, J. & Guillén-Gosálbez, G. Environmental sustainability assessment of hydrogen from waste polymers. *ACS Sustain. Chem. Eng.* **11**, 3238–3247 (2023).
9. Chemanalyst. Methylene dichloride price trend and forecast. (2025). Available at: https://www.chemanalyst.com/Pricing-data/methylene-dichloride-1092.
10. CEWEP. Landfill taxes and restrictions. (2025). Available at: https://www.cewep.eu/landfill-taxes-and-restrictions/.
11. Ioannou, I., Javaloyes-Antón, J., Caballero, J. A. & Guillén-Gosálbez, G. Economic and environmental performance of an integrated CO_2 refinery. *ACS Sustain. Chem. Eng.* **11**, 1949–1961 (2023).
12. World Bank. World Bank commodity price data. (2024). Available at: https://shorturl.at/v8ang.
13. GlobalPetrolPrice. Electricityprices for the ìndustry: world map. Available at: https://www.globalpetrolprices.com/map/electricity_industrial/.
14. Eurostat. Recycling – secondary material price indicator. (2025). Available at: https://shorturl.at/uJgdw.
15. Shangai Metal Market. Zirconium dioxide price charts. (2025). Available at: https://www.metal.com/en/prices/202302020006.
16. Shanghai Metal Market. Tungsten price charts. (2025). Available at: https://www.metal.com/Other-Minor-Metals/202302020006.
17. World Bank. Commodity Markets Outlook, April 2022: The Impact of the War in Ukraine on Commodity Markets. (2022). Available at: https://shorturl.at/bmMP8.
18. Trading Economics. Propane price chart – historical data. (2025). Available at: https://tradingeconomics.com/commodity/propane.
19. Business Analytiq. Gasoline price chart. (2025). Available at: https://businessanalytiq.com.
20. Business Analytiq. Diesel price chart. (2025). Available at: https://businessanalytiq.com/.

21. Business Analytiq. Fuel oil price chart. (2025). Available at: https://businessanalytiq.com.
22. Wernet, G. *et al.* The ecoinvent database version 3 (part I): overview and methodology. *Int. J. Life Cycle Assess.* **21**, 1218–1230 (2016).
23. Luyben, W. L. Estimating refrigeration costs at cryogenic temperatures. *Comput. Chem. Eng.* **103**, 144–150 (2017).
24. Nogueroles-Langa, I. *et al.* Polyethylene hydrogenolysis to liquid products over bimetallic catalysts with favorable environmental footprint and economics. *Nat. Commun.* **16**, 9791 (2025).
25. Jia, C. *et al.* Deconstruction of high-density polyethylene into liquid hydrocarbon fuels and lubricants by hydrogenolysis over Ru catalyst. *Chem Catal.* **1**, 437–455 (2021).
26. Qiu, Z. *et al.* A reusable, impurity-tolerant and noble metal–free catalyst for hydrocracking of waste polyolefins. *Sci. Adv.* **9**, eadg5332 (2025).
27. Liu, S., Kots, P. A., Vance, B. C., Danielson, A. & Vlachos, D. G. Plastic waste to fuels by hydrocracking at mild conditions. *Sci. Adv.* **7**, eabf8283 (2025).
28. Vance, B. C., Kots, P. A., Wang, C., Granite, J. E. & Vlachos, D. G. Ni/SiO_2 catalysts for polyolefin deconstruction via the divergent hydrogenolysis mechanism. *Appl. Catal. B Environ.* **322**, 122138 (2023).
29. Borkar, S. S., Helmer, R., Panicker, S. & Shetty, M. Investigation into the reaction pathways and catalyst deactivation for polyethylene hydrogenolysis over silica-supported cobalt catalysts. *ACS Sustain. Chem. Eng.* **11**, 10142–10157 (2023).
30. Wang, C. *et al.* Polyethylene hydrogenolysis at mild conditions over ruthenium on tungstated zirconia. *JACS Au* **1**, 1422–1434 (2021).
31. Rorrer, J. E., Troyano-Valls, C., Beckham, G. T. & Román-Leshkov, Y. Hydrogenolysis of polypropylene and mixed polyolefin plastic waste over Ru/C to produce liquid alkanes. *ACS Sustain. Chem. Eng.* **9**, 11661–11666 (2021).
