# Supplementary material for: Interfacial W–O–Zr ensembles in tungstated zirconia catalysts enable efficient hydrogen-free recycling of polypropylene waste
Source: Nat Commun. 2026 May 22;17:6708. doi: 10.1038/s41467-026-73420-6 (PMC13385888; doi:10.1038/s41467-026-73420-6)
Supplement: Supplementary file 2 — Transparent Peer Review file [file 41467_2026_73420_MOESM2_ESM.pdf]

nature portfolio

Peer Review File

Interfacial W–O–Zr ensembles in tungstated zirconia catalysts enable efficient hydrogen-free recycling of polypropylene waste

Corresponding Author: Professor Javier Pérez-Ramírez

This file contains all reviewer reports in order by version, followed by all author rebuttals in order by version.

Version 0:

Reviewer comments:

Reviewer #1

(Remarks to the Author)
This manuscript reports a hydrogen- and solvent-free strategy for converting PP into gasoline-range hydrocarbons under mild conditions (240 °C) using a flame-synthesized tungstated zirconia catalyst. The authors employed HAADF-STEM alongside operando XANES, EXAFS, and DRIFTS to identify W–O–Zr motifs as the active sites. The results demonstrate a temperature-driven restructuring that forms active center and elucidates the structure-performance relationship. In addition, a harmonized comparison across different chemical recycling technologies underscores the practical potential of this strategy for waste PP valorization. The study is well-executed, the characterizations are convincing and consistent, and the findings offer a high-performance solution for chemical recycling of waste PP. Therefore, I recommend publication once the following minor points are addressed:
1. The supplementary information provides the distribution of olefins and alkanes in the gaseous products, showing a ratio of approximately 2:1 (Supplementary Table 5). How is the distribution in the liquid phase? If the liquid products exhibit a similar ratio, it could imply that some olefins from β-scission PP are converted to alkanes. Please briefly discuss this process and clarify the role of catalyst in promoting such reactions.
2. The TPSR-MS was used to study the H_2 evolution during temperature-programmed surface reaction, but it is not clearly illustrated whether H_2 is generated in PP conversion in batch reactor. Have the authors performed any supplementary measurements (e.g., using a TCD) to confirm the presence or absence of H_2 in the gaseous products?
3. How is the carbon balance for PP conversion? Does the H/C ratio remain unchanged as reaction proceeds?
4. The article mentioned that after reaction for 10 min, 75% PP conversion with 88% gasoline selectivity was achieved. What was the temperature ramping rate? Was there enough time for the PP to melt and contact with catalytic sites?

Reviewer #2

(Remarks to the Author)
In this paper, the authors reported a series of zirconia supported tungsten oxide clusters for recycling of PP and evaluates their reaction mechanism and environmental/economic matrices. While the catalytic performance testing and the life cycle/techno-economic studies are thorough and represent an advanced contribution to the field, the characterization and analysis of the active sites are currently unconvincing. Significant evidence is required to address the following structural and mechanistic concerns before this manuscript can be considered for publication.

(1) The correlation between W-O-Zr sites and activity is not convincing. It is naturally expected for the density of interfacial W-O-Zr sites to peak at intermediate W loadings, as shown in Figure 1. at low loadings, the total amount of WO_x is insufficient to form a large interface, whereas at high loadings, cluster sintering reduces the specific interfacial area. Therefore, the W-O-Zr site percentage is inherently coupled to the loading and size of the WO_x species. The authors must clarify how they distinguish the unique effect of the interface from these overlapping factors and prove it is the key descriptor.

(2) The quantification of W-O-Zr sites relies almost exclusively on EXAFS analysis, which currently lacks rigor and contains internal inconsistencies.

(3) In Figure 4b (W L3-edge), the assignment of the feature at 3.15 Å to a W-Zr shell lacks direct evidence; a structural model and fitting results must be provided to justify this assignment.

(4) In Figure 4d, the label for the standard should be corrected to W0Zr100.

(5) The Zr K-edge spectra in Figure 4e are unusual and appear inconsistent with the room-temperature data provided for the same catalysts in Figure 5e and Figure S16-17. The peak assigned to W-Zr appears much larger than the Zr-O and Zr-Zr peaks, which should represent the dominant coordination shells in the WaZrb catalysts. The authors should provide EXAFS data for ZrO_2 standards fitted with a standard model for comparison and explicitly detail the structural model used for fitting the WaZrb catalysts.

(6) To ensure the reliability and quality of the spectroscopic data, the authors must provide the k-space data for all EXAFS measurements, including the W L3-edge and Zr K-edge. Additionally, the R-space imaginary components for the fitting should be provided for all datasets to allow for a proper assessment of the fit quality. The structural model used for the fitting must also be clearly presented to confirm its physical reasonableness.

(7) Regarding the CN of Zr-O (SI Table 9), the authors report the CN increases to 10 or even 12 with increasing temperature. This is physically unreasonable, and the authors must provide a detailed explanation and evidence for this. The model and fitting details should be carefully checked and provided.

(8) According to Figure S14, there are clear variations in the acidic properties across the catalyst series. These observations appear to contradict the authors' conclusion that a direct relationship between catalytic activity and acid type, density, or strength can be discounted. Currently, the role of acidity remains inadequately addressed in the discussion and should be discussed.

(9) The authors mentioned Zr-W-silica catalysts are known for converting PE. Then the authors should compare their catalysts with the Zr-W silica/zeolite material for PP conversion performance, and also discuss the differences in terms of mechanism.

(10) How is the catalysts' performance for PE upcycling? Especially whether the reaction mechanism they elucidated is general for polyolefins or specific for PP.

(11) The authors have demonstrated, via DRIFTS, a specific interaction between the W-O-Zr sites and the reactant. Could the authors rationalize, through electronic structure calculation or qualitative but intuitive explanation, why does the W-O-Zr site possess this activity, while the homonuclear W-O-W/Zr-O-Zr sites do not?

Reviewer #3

(Remarks to the Author)
The authors reported hydrogen- and solvent-free depolymerization of polypropylene at 240 °C, achieving >80% yield into gasoline hydrocarbons with the aid of Tungsten- zirconia catalysts pyrolysis. These acid sites formed during reaction mediate internal hydrogen transfer and selective backbone C–C scission. Life cycle and technoeconomic studies have been done and indicated competitive environmental and economic advantages. This work could be published in Nature Communications after major revision.
Comments:
1- The authors claim that the reported study is the first efficient depolymerization of polypropylene (PP) in the absence of hydrogen or any other reductant at mild temperatures. The sentence should be specific for Tungsten- zirconia system and the mentioned references should be updated.
2- In catalyst formulation, nanostructure of WaZrb catalysts, Fig 1b and 1c should be presented at the same nm scale, it seems that W10Zr90 showing WOx sub-nanoclusters and an isolated $[WO_4]^{2-}$ species as well. The discussion in this part should be explained based on the correction.
3- The authors should explain why low MW polypropylene was chosen for the prescreen process rather than the high MW one.
4- N_2 4 bar pressure is chosen as optimum pressure for pyrolysis condition, is there any investigation for the pressure effect on the recovered products
5- Give full explanation of why increasing temperature progressively narrowed the product distribution into the C4-C10 range?
6- Supplementary Table 2, showed that C4-C12 yield% dramatically improved from 4% to 59%, by changing the catalyst ratio from W1Zr99 to W3Zr97. Further detailed discussion is needed to explain that finding.
7- Proposed reaction pathway Fig 6e, showed alkane and alkene products. The authors should investigate the alkane/alkene ratios or explain why the final products are saturated alkane in the absence of H_2. (ACS Applied Polymer Materials 2024 6 (16), 9866-9874)
8- There is no any scale up production experiment.

Version 1:

Reviewer comments:

Reviewer #1

(Remarks to the Author)
The authors have addressed the comments and concerns of the reviewers. The revisions have improved the quality of the paper. I recommend that the manuscript be accepted for publication as is.

Reviewer #2

(Remarks to the Author)
The authors have substantially improved the manuscript，so I now recommend it for publication.

Reviewer #3

(Remarks to the Author)

Open Access This Peer Review File is licensed under a Creative Commons Attribution 4.0 International License, which permits use, sharing, adaptation, distribution and reproduction in any medium or format, as long as you give appropriate credit to the original author(s) and the source, provide a link to the Creative Commons license, and indicate if changes were made.
In cases where reviewers are anonymous, credit should be given to 'Anonymous Referee' and the source.
The images or other third party material in this Peer Review File are included in the article's Creative Commons license, unless indicated otherwise in a credit line to the material. If material is not included in the article's Creative Commons license and your intended use is not permitted by statutory regulation or exceeds the permitted use, you will need to obtain permission directly from the copyright holder.
To view a copy of this license, visit https://creativecommons.org/licenses/by/4.0/

Response to Reviewers for Manuscript NCOMMS-25-105549

Code: Comments in blue | Replies in black | Actions in **bold**

Note: Indicated page, line, figure, or reference numbers refer to the revised manuscript and/or supplementary information with changes highlighted.

Reviewer 1

This manuscript reports a hydrogen- and solvent-free strategy for converting PP into gasoline-range hydrocarbons under mild conditions (240 °C) using a flame-synthesized tungstated zirconia catalyst. The authors employed HAADF-STEM alongside operando XANES, EXAFS, and DRIFTS to identify W–O–Zr motifs as the active sites. The results demonstrate a temperature-driven restructuring that forms active center and elucidates the structure-performance relationship. In addition, a harmonized comparison across different chemical recycling technologies underscores the practical potential of this strategy for waste PP valorization. The study is well-executed, the characterizations are convincing and consistent, and the findings offer a high-performance solution for chemical recycling of waste PP. Therefore, I recommend publication once the following minor points are addressed:

We thank the reviewer for the careful and very positive assessment of our work. The revised version of the manuscript addresses all their concerns. In addition, the following elements strengthening catalyst characterization and scope have been incorporated: (i) a refined analysis of XAS spectra for previous and new samples, (ii) a study of the transferability of performance and mechanisms across polyolefins and catalyst structures, (iii) an deeper discussion about the nature of the bridging oxygen in W–O–Zr ensembles, and (iv) additional evidence of high performance under new operating conditions.

1.1. The supplementary information provides the distribution of olefins and alkanes in the gaseous products, showing a ratio of approximately 2:1 (Supplementary Table 5). How is the distribution in the liquid phase? If the liquid products exhibit a similar ratio, it could imply that some olefins from *b*-scission PP are converted to alkanes. Please briefly discuss this process and clarify the role of catalyst in promoting such reactions.

The detailed distribution of products was provided for the gas phase as their individual quantification is feasible. The variety of liquid products precludes this option. However, ^{1}H NMR spectra of liquid products in the Supplementary Fig. 10 reveals a small contribution from olefinic groups ($H_{olefins}/H_{aliphatic}$ = 0.06). Guided from an observation of Reviewer 3, the transformation of a fraction of alkenes to alkanes is plausible when considering that the H:C ratio of the solid residue decreases from 2.1 to 1.8 over the course of the reaction (Supplementary Fig. 11), in close alignment with the detection of H_2 in the products (see reply to comment 1.2). Olefins may thus be hydrogenated resulting in an increased fraction of alkanes. **The revised manuscript includes now this discussion in page 12, line 15.**

1.2. The TPSR-MS was used to study the H_2 evolution during temperature-programmed surface reaction, but it is not clearly illustrated whether H_2 is generated in PP conversion in batch reactor. Have the authors performed any supplementary measurements (e.g., using a TCD) to confirm the presence or absence of H_2 in the gaseous products?

H_2 is produced by in the batch reactor in small amounts or mostly react with alkenes to produce alkanes. The H_2 yield measured for the reference $W_{10}Zr_{90}$ catalyst under standard reaction

conditions was 0.2%. The corresponding GC-TCD signal is available in the **Supplementary Fig. 12**. **The revised version mentions this fact in page 5, line 27**.

1.3. How is the carbon balance for PP conversion? Does the H/C ratio remain unchanged as reaction proceeds?

The calculations for the different product fractions operate based on a close carbon balance. The mass of gaseous products is calculated by weighing the glass inset before and after reaction. The solid residue is then calculated after dissolution of liquid and solid products by weighing the glass inset again. The weight of liquid products is then calculated. The liquid and gas distributions were determined by liquid and gas GC analysis, respectively. **This aspect is better highlighted now in the Methods section (page 19, line 20).**

The evolution of the H:C ratio of the solid residue decreases from 2.1 to 1.8 after 24 h (Supplementary Fig. 10). This interesting feature suggests that a fraction of the feedstock acts as hydrogen donor, partly explaining the abundance of alkanes (see reply to comments 1.1 and 1.2) through a parallel mechanism requiring further investigations. **The revised version extends the description of this fact and elaborates on its significance based on the previous discussion (page 5, line 27).**

1.4. The article mentioned that after reaction for 10 min, 75% PP conversion with 88% gasoline selectivity was achieved. What was the temperature ramping rate? Was there enough time for the PP to melt and contact with catalytic sites?

We appreciate the opportunity to clarify a potentially unclear aspect about catalyst evaluation. Nominal conditions are reached by first pressurizing the reactor followed by a heating ramp controlled by the PID system to avoid overshooting, resulting in ca. 3 °C min^{-1} on average. Stirring then commences and reaction time starts counting for consistency across experiments. In the referred case of 10 min, the polymer was melted for around 15 min prior to the start of the reaction. **The Methods section now contains this information (page 19, line 2).**

Reviewer 2

In this paper, the authors reported a series of zirconia supported tungsten oxide clusters for recycling of PP and evaluates their reaction mechanism and environmental/economic matrices. While the catalytic performance testing and the life cycle/techno-economic studies are thorough and represent an advanced contribution to the field, the characterization and analysis of the active sites are currently unconvincing. Significant evidence is required to address the following structural and mechanistic concerns before this manuscript can be considered for publication.

We are grateful for a strong support to key areas. All concerns have been addressed, leading to strengthened conclusions. In brief, all previous XAS analyses have been carefully reassessed under refined models, for which all data is now provided together with a dedicated Supplementary Note. In this regard, new analysis of samples carrying intermediate tungsten contents confirmed the direct connection between catalytic activity and the density of W–O–Zr ensembles. Additionally, a transferability study across reference catalysts and polymers uncovers now mechanistic similarities and differences pointing to polyolefin-specific catalyst design needs.

Regarding the catalyst characterizations

2.1. The correlation between W-O-Zr sites and activity is not convincing. It is naturally expected for the density of interfacial W-O-Zr sites to peak at intermediate W loadings, as shown in Figure 1. at low loadings, the total amount of WO_x is insufficient to form a large interface, whereas at high loadings, cluster sintering reduces the specific interfacial area. Therefore, the W-O-Zr site percentage is inherently coupled to the loading and size of the WO_x species. The authors must clarify how they distinguish the unique effect of the interface from these overlapping factors and prove it is the key descriptor.

Fig. 2b shows how performance depends on the tungsten content, providing first evidence on the interfacial dependency. In parallel, catalytic performance relates linearly to the $CN_{Zr\text{-}W}$ ratio (Fig. 4f), to which new experimental points were added for catalysts carrying intermediate tungsten contents (W_5Zr_{95}, $W_{30}Zr_{70}$, $W_{40}Zr_{60}$, and $W_{80}Zr_{20}$). The larger number of experimental data revealed a volcano-type dependency, like the one predicted by the reviewer, between tungsten content and $CN_{Zr\text{-}W}$ ratio, unambiguously confirming the $CN_{Zr\text{-}W}$ ratio as proxy for the density of W–O–Zr sites and thus, considering all the above, their role as active sites. **This discussion is now available in the revised text (page 8, lines 6, 12) and is supported by the Supplementary Fig. 19, revised Supplementary Tables 2, 11 and the new points in Fig. 4f.**

2.2. The quantification of W–O–Zr sites relies almost exclusively on EXAFS analysis, which currently lacks rigor and contains internal inconsistencies.

We have thoroughly revised EXAFS data to assure the highest accuracy and consistency. Actions are explained in detail in the responses to comments 2.1, 2.3, 2.5, 2.6, and 2.7. In brief, (i) comprehensive information is now provided regarding the structural models, scattering paths, fitting strategy, and parameter constraints, including the addition of all k-space fitting data for all samples along with the R-spectra and a summary in the **Supplementary Note 3**; (ii) observed trends were confirmed by four additional catalysts with varying tungsten contents; (iii) the presence of W–O–Zr sites was reinforced by new fitting analyses at the W L_3-edge; (iv)

the assignment of the W–Zr feature was carefully re-examined; (v) a more refined assessment of the operando Zr K-edge EXAFS data resolved physically unlikely Zr–O coordination numbers.

2.3. In Figure 4b (W L_3-edge), the assignment of the feature at 3.15 Å to a W-Zr shell lacks direct evidence; a structural model and fitting results must be provided to justify this assignment.

We have performed W L_3-edge EXAFS fitting for the catalyst series using a $Zr(WO_4)_2$-derived model for interfacial W–Zr environments and a WO_3-derived model for W–W environments in condensed WO_x/WO_3 domains. The W–Zr coordination number decreases with increasing tungsten content, while the W–W coordination number increases. Meanwhile, the local W–O coordination evolves from a more distorted $Zr(WO_4)_2$-like environment toward a more ordered WO_3-like environment. This trend indicates a structural evolution from isolated species towards a more condensed WO_x domains and finally WO_3-rich structures, in agreement with the Zr K-edge analysis. Accordingly, **we state in the revised version that the 3.0–3.5 Å region contains overlapping W–Zr and W–W contributions supported by the W L_3-edge fitting results. Fitting results, together with major scattering paths and fitted spectra, are elaborated in the revised manuscript (page 7, line 15), supported by the revised Fig. 4, and provided in the Supplementary Table 10 and Supplementary Figs. 16, 17**.

2.4. In Figure 4d, the label for the standard should be corrected to W_0Zr_{100}.

We thank the reviewer for this observation. The label is now correct in the revised version.

2.5. The Zr K-edge spectra in Figure 4e are unusual and appear inconsistent with the room-temperature data provided for the same catalysts in Figure 5e and Figure S16-17. The peak assigned to W-Zr appears much larger than the Zr-O and Zr-Zr peaks, which should represent the dominant coordination shells in the W_aZr_b catalysts. The authors should provide EXAFS data for ZrO_2 standards fitted with a standard model for comparison and explicitly detail the structural model used for fitting the W_aZr_b catalysts.

The coloured shading in Fig. 4e highlighted that the second-shell region contains overlapping contributions from both Zr–Zr and Zr–W scattering paths. **We have revised the figure annotation to avoid misinterpretation**.

The inconsistency between spectra originated from an error during data export for graphical representation. Spectra in Fig. 4e were inadvertently plotted as k^3-weighted Fourier-transformed spectra rather than the k^2-weighted form used elsewhere, making the contribution of the second-shell region appear artificially enlarged. **The k^2-weighted spectra now displayed in Fig. 4e are consistent with Fig. 5e and the Supplementary Figs. 16, 18**.

Following the reviewer's suggestion, **we have added the EXAFS fitting results of the ZrO_2 standard using a tetragonal ZrO_2 model for comparison, with the structural model used to fit the series of W_aZr_b catalysts provided in Supplementary Table 11.** Briefly, the W_aZr_b fitting was constructed based on the Zr–O and Zr–Zr paths derived from tetragonal ZrO_2, with an additional Zr–W scattering path introduced to account for interfacial WO_x–ZrO_2 interactions.

2.6. To ensure the reliability and quality of the spectroscopic data, the authors must provide the k-space data for all EXAFS measurements, including the W L_3-edge and Zr K-edge. Additionally, the R-space imaginary components for the fitting should be provided for all datasets to allow for a proper assessment of the fit quality. The structural model used for the fitting must also be clearly presented to confirm its physical reasonableness.

The k-space data for all EXAFS measurements together with the R-space imaginary components of the fitting and structural models for all previous and new samples are now available in Supplementary Figs. 16-18, 22, 23 and Supplementary Tables 10-12.

2.7. Regarding the CN of Zr-O (SI Table 9), the authors report the CN increases to 10 or even 12 with increasing temperature. This is physically unreasonable, and the authors must provide a detailed explanation and evidence for this. The model and fitting details should be carefully checked and provided.

We have refitted the operando Zr *K*-edge EXAFS data using a tetragonal ZrO_2-based model, in which the first-shell Zr–O contribution was described by two subshells (Zr–O_1 and Zr–O_2 paths). The average total Zr–O coordination number stays approximately constant at 8.0 across temperatures, aligned with a tetragonal ZrO_2 local coordination environment. **We have revised the discussion in the main text (page 10, line 2) and updated Supplementary Tables 11, 12 accordingly. Fitting models and results are available in Supplementary Figs. 16, 17, 22, 23**.

2.8. According to Figure S14, there are clear variations in the acidic properties across the catalyst series. These observations appear to contradict the authors' conclusion that a direct relationship between catalytic activity and acid type, density, or strength can be discounted. Currently, the role of acidity remains inadequately addressed in the discussion and should be discussed.

First discussions on acidity features behind the excellent performance of $W_{10}Zr_{90}$ can be developed based on pyridine FTIR and NH_3-TPD results. Their ex-situ character nonetheless precludes reliably establishing causality.

Pyridine FTIR results (Supplementary Fig. 14b) show that the reference $W_{10}Zr_{90}$ catalyst and zirconia (W_0Zr_{100}) contain similar distributions of Lewis and Brønsted sites despite very different performance (Fig. 1). The comparison with tungsten oxide ($W_{100}Zr_0$) suggests that two different Lewis acid sites (1600 cm^{-1}, 1450 cm^{-1}) might have a catalytic role, but their presence in W_0Zr_{100} makes this hypothesis unlikely. The only distinctive feature for $W_{10}Zr_{90}$ is thus the lower magnitude of a band associated to Lewis acidity at ca. 1470 cm^{-1}.

Regarding NH_3-TPD profiles, it is possible to infer that strong acid sites, only present in W_0Zr_{100}, might be detrimental. $W_{10}Zr_{90}$ displays a broad beak covering from weak to medium strength sites. However, W_0Zr_{100}, $W_{60}Zr_{40}$, and $W_{100}Zr_0$ also possess abundant sites in this region while showing clearly different performance. The distinctive feature is thus the higher abundancy of sites originating peaks at ca. 283 °C in W_3Zr_{97} and $W_{10}Zr_{90}$.

In summary, effective depolymerization might demand certain type of medium-strength sites more apparent on W_3Zr_{97} and $W_{10}Zr_{90}$ that may relate to W–O–Zr structures. **A related discussion can now be found in the revised version (page 8, line 22)**.

2.9. The authors mentioned Zr-W-silica catalysts are known for converting PE. Then the authors should compare their catalysts with the Zr-W silica/zeolite material for PP conversion performance, and also discuss the differences in terms of mechanism.

The referred catalyst comprises an oxidic WZr phase hosted by KIT6 mesoporous silica and physically mixed with HZSM-5 (Chem **11**, 102340 (2025), reference 24). The transferability of performance across materials was studied by synthesizing and evaluating WZr-KIT6+HZSM-5 for PP depolymerization under the standard conditions used in our work (**Supplementary Table 9**). This catalyst yielded 20% lower conversion than $W_{10}Zr_{90}$, confirming the high activity of W-Zr ensembles and suggesting that efficient processing of PP may not need additional zeolite catalytic functions. **This discussion can be found in page 6, line 22, supported by the Supplementary Table 9. Catalyst synthesis for WZr-KIT6+HZSM-5 is available in the Methods section (page 15, line 20).**

2.10. How is the catalysts' performance for PE upcycling? Especially whether the reaction mechanism they elucidated is general for polyolefins or specific for PP.

The evaluation of $W_{10}Zr_{90}$ for HDPE depolymerization under the standard conditions used in our work (**Supplementary Table 13**) revealed stark differences across polyolefins illustrated by the contrasting conversions for HDPE (24%) and PP (92%). The existence of tertiary and primary carbons in the PP structure thus seems to be a key structural feature for the efficient operation of W–O–Zr ensembles. This effect requires further investigations beyond the scope of this work. However, the need for polyolefin-targeted catalyst design now emerges as a result of this study. **We have added this discussion to the revised version (page 12, line 27) supported by the Supplementary Table 13**.

2.11. The authors have demonstrated, via DRIFTS, a specific interaction between the W-O-Zr sites and the reactant. Could the authors rationalize, through electronic structure calculation or qualitative but intuitive explanation, why does the W-O-Zr site possess this activity, while the homonuclear W-O-W/Zr-O-Zr sites do not?

A main conclusion of the article is the unique acidic properties of the bridging oxygen in interfacial W–O–Zr ensembles. The difference in electronegativity between tungsten (1.7) and zirconium (1.4) is expected to favor displacement of the oxygen electronic density toward the tungsten center, leaving the bridging oxygen with a less negative charge. This will reflect in milder acidity than the corresponding site in homonuclear materials, in alignment with the prevalence of acid sites with medium strength in $W_{10}Zr_{90}$ (Supplementary Fig. 20). **This discussion was added to page 10, line 22**.

Reviewer 3

The authors reported hydrogen- and solvent-free depolymerization of polypropylene at 240 °C, achieving >80% yield into gasoline hydrocarbons with the aid of Tungsten- zirconia catalysts pyrolysis. These acid sites formed during reaction mediate internal hydrogen transfer and selective backbone C–C scission. Life cycle and technoeconomic studies have been done and indicated competitive environmental and economic advantages. This work could be published in Nature Communications after major revision.

We thank the reviewer for their appreciation of our work and for the positive recommendation. All their concerns have been addressed. In addition, the revised manuscript now includes: (i) a refined interpretation of the XAS data including new samples with intermediate tungsten contents confirming the direct link between catalyst performance and the density of W–O–Zr ensembles, (ii) an evaluation of the transferability of catalytic performance across different polyolefins and catalyst architectures, and (iii) an expanded discussion addressing the role and characteristics of the bridging oxygen species in W–O–Zr.

3.1. The authors claim that the reported study is the first efficient depolymerization of polypropylene (PP) in the absence of hydrogen or any other reductant at mild temperatures. The sentence should be specific for Tungsten- zirconia system and the mentioned references should be updated.

After an extensive analysis of the recent literature, including a specific review on catalytic H_2-free routes for polyolefin chemical recycling (Korean J. Chem. Eng. **43**, 109–146 (2026)), we could not find reports aiming at the design of catalysts for PP depolymerization at mild temperatures. We nonetheless added the following references for other processes where mild conditions were used to further clarify the context of this investigation. The first work showed that a USY zeolite was active to unreported products in thermogravimetric experiments (Cell Reports Phys. Sci. **6**, 12 (2025)). A second report (provided by the reviewer in comment 3.7) contains a non-catalytic depolymerization of PP using H_2 absorbers (ACS App. Polym. Mater. **6**, 9866-9874 (2024)). A third report used mechanochemical means to transform PP using zeolites (Catal. Sci. Technol. **15**, 7525–7538 (2025)). **We have added these references (32-35) and clarified that our work is the first one targeting catalyst design for PP at mild conditions in the absence of auxiliary means (page 4, line 2)**.

3.2. In catalyst formulation, nanostructure of W_aZr_b catalysts, Fig 1b and 1c should be presented at the same nm scale, it seems that $W_{10}Zr_{90}$ showing WO_x sub-nanoclusters and an isolated $[WO_4]^{2-}$ species as well. The discussion in this part should be explained based on the correction.

Referred panels are now shown at the same scale. Isolated W centers is the predominant speciation in W_3Zr_{97}, while WO_x sub-nanoclusters predominate in $W_{10}Zr_{90}$. This does not preclude the existence of both speciations across materials. **This fact is further clarified in the text (page 4, lines 20, 22) with the support of additional micrographs in Supplementary Fig. 2, 3**.

3.3. The authors should explain why low M_W polypropylene was chosen for the prescreen process rather than the high M_W one.

A PP with a molecular weight found in consumer goods (PP_{12k}, M_w = 12k kDa) was chosen after early experiments showing preserved activity compared to higher molecular weights

(Fig. 1b) due to its lower melt viscosity amenable to techniques such as FTIR and TPSR. **We added a note on this aspect to the Methods section (page 18, line 21)**.

3.4. N_2 4 bar pressure is chosen as optimum pressure for pyrolysis condition, is there any investigation for the pressure effect on the recovered products

The revised manuscript now includes catalytic evaluations showing a slight inverse correlation between conversion and pressure (**Supplementary Fig. 8**) with almost no effect on the product distribution. This may be linked to the high fraction of C_4-C_6 products (Supplementary Tables 2-6). Increasing the total pressure may hinder the removal of these compounds from the melt, increasing their local concentration and, in turn, decreasing their formation rate. **The revised version contains a brief discussion in page 5, line 10 on this trend based on the Supplementary Fig. 8 and Supplementary Table 12.**

3.5. Give full explanation of why increasing temperature progressively narrowed the product distribution into the C_4-C_{10} range?

As discussed in the original version of the manuscript, the most plausible reason for the abundance of C_4-C_6 products is that tertiary carbenium ions are much more stable than smaller ones (Nat. Nanotechnol. **18**, 772–779 (2023)), while the smallest fragment originating from PP able to accommodate them is iso-C_4. Depolymerization at mild temperatures may thus stall around this chain length. It follows that higher conversions attained at increasing reaction temperatures preferentially leads to a higher C_4-C_{10} selectivity. **The revised version contains now a more detailed explanation of this observation (page 6, line 7)**.

3.6. Supplementary Table 2, showed that C_4-C_{12} yield % dramatically improved from 4% to 59%, by changing the catalyst ratio from W_1Zr_{99} to W_3Zr_{97}. Further detailed discussion is needed to explain that finding.

We have repeated catalytic evaluations for these two materials and confirmed that an error in the product quantification of the original measurements led to a distorted result. The newly quantified C_4-C_{12} yield is 27% for W_1Zr_{99} and 56% for W_3Zr_{97}, making the transition from W_0Zr_{100} (18%) smoother. **The correct values can be found in Supplementary Table 2**.

3.7. Proposed reaction pathway Fig 6e, showed alkane and alkene products. The authors should investigate the alkane/alkene ratios or explain why the final products are saturated alkane in the absence of H_2. (ACS Applied Polymer Materials 2024 6 (16), 9866-9874)

The alkane:alkene ratio is ca. 2:1 in the C_1-C_5 range (Supplementary Table 5). As the proposed mechanism predicts a 1:1 ratio (Fig. 6e), a secondary mechanism must be at play generating a surplus of alkanes. As discussed in the original version and illustrated in the Supplementary Fig. 11, the H:C ratio of the residue decreases from 2.1 (characteristic of PP) to ca. 1.8 over course of a 24 h reaction. In line with the detection of H_2 in batch experiments (0.2 % yield, see **Supplementary Fig. 12**), it is highly plausible that a fraction of PP undergoes dehydrogenation, creating the conditions for alkenes to be hydrogenated. This is aligned with the suggested mechanism proposed for non-catalytic pyrolysis in the provided reference. **We added the corresponding discussion in page 12, line 15 supported by the suggested work (reference 34)**.

3.8. There is no any scale up production experiment.

We have performed a new set of experiments using the reference $W_{10}Zr_{90}$ catalyst for the processing of 3 g of PP (practical limit in our lab-scale reactor setup). We observed a high conversion (72%) with unmodified gasoline selectivity compared to the nominal case of 0.5 g. **The results are provided in Supplementary Table 7 and mentioned in the text in page 6, line 14.**
